# Synthesis and characterisation of new silicon–perfluoropropenyl compounds†

**DOI:** 10.1039/d3ra01353g

**Published:** 2023-05-03

**Authors:** Lulu Alluhaibi, Alan Brisdon, Sylwia Klejna, Abeer Muneer

**Affiliations:** a Academic Centre for Materials and Nanotechnology, AGH University of Science and Technology ul. Kawiory 30 30-055 Kraków Poland lulu.alluhaibi@gmail.com; b School of Chemistry, The University of Manchester Manchester M13 9PL UK

## Abstract

Novel, stable silicon–pentafluoropropane compounds have been synthesised from the direct reaction of hydrofluorocarbons *Z*-CFH=CFCF_3_ (*Z*-HFC-1225ye) with ^*n*^BuLi, followed by appropriate silicon-halide. The compounds have been characterized by multinuclear NMR studies (^19^F, ^1^H, ^29^Si and ^13^C), DFT studies and structural confirmation was obtained by X-ray diffraction. Based on the outcome of treating synthetic silicon–pentafluoropropene compounds with different nucleophilic sources (^*n*^BuLi, ^*t*^BuLi, MeLi, and PhLi) and computed for this reaction DFT energetics, it is clear that the C–F_*trans*_ bond is more active than C–F_*gem*_ (F_*gem*_ and F_*trans*_ are labelled with respect to Si). This provides a route for efficient modification of pentafluoropropene group, that can be a crucial step in developing pharmaceuticals that include propenyl or vinyl groups, addressing the demand for medicines based on long carbonic chains.

## Abstract 2

Novel, stable silicon–pentafluoropropane compounds have been synthesised from the direct reaction of hydrofluorocarbons *Z*-CFH=CFCF_3_ (*Z*-HFC-1225ye) with ^*n*^BuLi, followed by appropriate silicon-halide.

## Graphical abstract

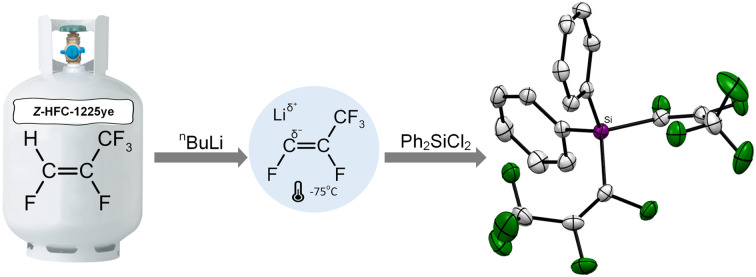

## Introduction

Fluorine plays an important role in the medical field, particularly in pharmacological developments ranging from perfluorinated fluids used as artificial blood and fluoropolymers used in grafts, through applications in drug delivery and in improving the metabolic stability of new medications.^1^ It has been found that at least one fluorine moiety is present in 37% of all active small molecule pharmaceutical ingredients that have been approved by the FDA in 2020. Furthermore, between 2011 and 2020, a 26% increase in fluorine-containing pharmaceuticals in all pharmaceuticals approved by the FDA has been noted.^2^

**Fig. 1:**
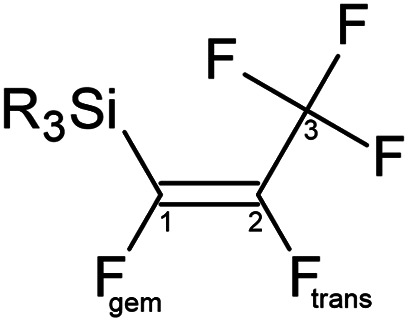
Skeleton of general structure of R_3_Si(*E*-CF=CFCF_3_).

Due to the importance of fluorocarbon fragments in pharmaceuticals, a number of studies covered the methods of attaching the fluorocarbon fragment into organic compounds^3^ or transition-metal complexes,^4^ as well as C–F bond activation have been reported.^5^ Unfortunately, there is a lack of studies of pentafluoropropene group (CF=CFCF_3_) comparing to analogues perfluorocarbon groups, such as trifluoromethyl CF_3_ and trifluoroethene (CF=CF_2_).^6^ Therefore, this paper focuses on new silicon-based perfluoropropenyl compounds, which would be suitable for transferring that fluorocarbon fragment *via* a Hiyama cross coupling reaction into suitable organic substrates. Although these transfers have already been done for tin-containing compounds,^7^ the silicon analogues would be preferred because the majority of silicon compounds are non-toxic and commercially available.^8^ We synthesized a series of silicon–pentafluoropropene compounds in *E* configuration with the general formula presented in Fig. 1 and Table 1. The obtained compounds have been fully characterized by multinuclear NMR studies (^19^F, ^1^H, ^29^Si and ^13^C). The second part of this paper focuses on the investigation of the C–F bond activation through treating synthetic silicon–pentafluoropropene compounds with different nucleophilic sources (^*n*^BuLi, ^*t*^BuLi, MeLi, and PhLi). The DFT energetics have been also computed for this reaction.

**Table 1:** Summary of successfully synthesised 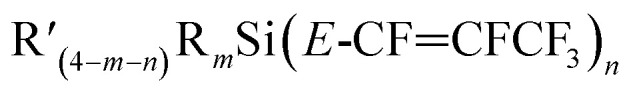 compounds with ^19^F{^1^H} NMR data (376 MHz, CDCl_3_, 291 K), (F_*gem*_ and F_*trans*_ are labelled with respect to Si)

Compound	*δ* CF_3_	*δ* F_*gem*_	*δ* F_*trans*_
(Et)_3_Si(*E*-CF=CFCF_3_) (a.1)	−68.13 ppm (d.d)	−137.16 ppm (q.d)	−141.20 ppm (q.d)
	^3^ *J* CF_3_ F_*trans*_ = 13.8 Hz	^4^ *J* F_*gem*_ CF_3_ = 6.5 Hz	^3^ *J* F_*trans*_ CF_3_ = 13.8 Hz
	^4^ *J* CF_3_ F_*gem*_ = 6.5 Hz	^3^ *J* F_*gem*_ F_*trans*_ = 13.0 Hz	^3^ *J* F_*trans*_ F_*gem*_ = 13.5 Hz
(Bu)_3_Si(*E*-CF=CFCF_3_) (a.2)	−67.91 ppm (d.d)	−136.57 ppm (q.d)	−141.33 ppm (q.d)
	^3^ *J* CF_3_ F_*trans*_ = 13.7 Hz	^4^ *J* F_*gem*_ CF_3_ = 6.2 Hz	^3^ *J* F_*trans*_ CF_3_ = 13.8 Hz
	^4^ *J* CF_3_ F_*gem*_ = 6.4 Hz	^3^ *J* F_*gem*_ F_*trans*_ = 11.9 Hz	^3^ *J* F_*trans*_ F_*gem*_ = 11.8 Hz
ClCH_2_(Me)_2_Si(*E*-CF=CFCF_3_) (a.3)	−67.83 ppm (d.d)	−139.02 ppm (m)	−139.14 ppm (m)
	^3^ *J* CF_3_ F_*trans*_ = 13.3 Hz		
	^4^ *J* CF_3_ F_*gem*_ = 6.2 Hz		
^*n*^Bu(Me)_2_Si(*E*-CF=CFCF_3_) (a.4)	−67.62 ppm (d.d)	−137.26 ppm (q.d)	−141.89 ppm (q.d)
	^3^ *J* CF_3_ F_*trans*_ = 13.7 Hz	^4^ *J* F_*gem*_ CF_3_ = 6.8 Hz	^3^ *J* F_*trans*_ CF_3_ = 13.2 Hz
	^4^ *J* CF_3_ F_*gem*_ = 6.5 Hz	^3^ *J* F_*gem*_ F_*trans*_ = 12.2 Hz	^3^ *J* F_*trans*_ F_*gem*_ = 12.8 Hz
Ph(Me)_2_Si(*E*-CF=CFCF_3_) (a.5)	−67.36 ppm (d.d)	−136.30 ppm (q.d)	−140.89 ppm (q.d)
	^3^ *J* CF_3_ F_*trans*_ = 13.2 Hz	^4^ *J* F_*gem*_ CF_3_ = 6.2 Hz	^3^ *J* F_*trans*_ CF_3_ = 13.2 Hz
	^4^ *J* CF_3_ F_*gem*_ = 6.3 Hz	^3^ *J* F_*gem*_ F_*trans*_ = 12.9 Hz	^3^ *J* F_*trans*_ F_*gem*_ = 12.9 Hz
Me(Ph)_2_Si(*E*-CF=CFCF_3_) (a.6)	−67.06 ppm (d.d)	−134.12 ppm (q.d)	−138.22 ppm (q.d)
	^3^ *J* CF_3_ F_*trans*_ = 13.7 Hz	^4^ *J* F_*gem*_ CF_3_ = 6.1 Hz	^3^ *J* F_*trans*_ CF_3_ = 13.0 Hz
	^4^ *J* CF_3_ F_*gem*_ = 6.2 Hz	^3^ *J* F_*gem*_ F_*trans*_ = 12.2 Hz	^3^ *J* F_*trans*_ F_*gem*_ = 12.5 Hz
(Me)_2_Si(*E*-CF=CFCF_3_)_2_ (a.7)	−68.83 ppm (d.d)	−137.75 ppm (q.d)	−140.36 ppm (m)
	^3^ *J* CF_3_ F_*trans*_ = 13.2 Hz	^4^ *J* F_*gem*_ CF_3_ = 5.8 Hz	
	^4^ *J* CF_3_ F_*gem*_ = 5.4 Hz	^3^ *J* F_*gem*_ F_*trans*_ = 12.7 Hz	
(^i^Pr)_2_Si(*E*-CF=CFCF_3_)_2_ (a.8)	−68.97 ppm (d.d)	−136.95 ppm (q.d)	−138.54 ppm (m)
	^3^ *J* CF_3_ F_*trans*_ = 14.1 Hz	^4^ *J* F_*gem*_ CF_3_ = 4.8 Hz	
	^4^ *J* CF_3_ F_*gem*_ = 5.8 Hz	^3^ *J* F_*gem*_ F_*trans*_ = 12.7 Hz	
(Ph)_2_Si(*E*-CF=CFCF_3_)_2_ (a.9)	−67.95 ppm (d.d.d)	−133.95 ppm (q.d)	−136.94 ppm (m)
	^3^ *J* CF_3_ F_*trans*_ = 13.0 Hz	^4^ *J* F_*gem*_ CF_3_ = 5.5 Hz	
	^4^ *J* CF_3_ F_*gem*_ = 4.5 Hz	^3^ *J* F_*gem*_ F_*trans*_ = 13.1 Hz	
	*J* CF_3_ F_external_ = 3.9 Hz		
PhSi(*E*-CF=CFCF_3_)_3_ (a.10)	−69.31 ppm (broad d)	−131.04 ppm (q.d)	−141.08 ppm (m)
	^3^ *J* CF_3_ F_*trans*_ = 13.4 Hz	^4^ *J* F_*gem*_ CF_3_ = 5.9 Hz	
		^3^ *J* F_*gem*_ F_*trans*_ = 13.5 Hz	
Si(*E*-CF=CFCF_3_)_4_ (a.11)	−70.05 ppm (d.m)	−127.32 ppm (q.d)	−145.45 ppm (m)
	^3^ *J* CF_3_ F_*trans*_ = 16.1 Hz	^4^ *J* F_*gem*_ CF_3_ = 6.5 Hz	
		^3^ *J* F_*trans*_ F_*gem*_ = 13.0 Hz	

## Results and discussion

### Synthesis of silicon–perfluoropropenyl compounds

**Scheme 1:**
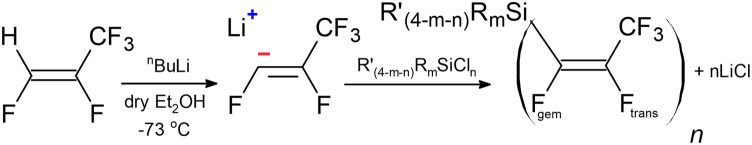
The general synthesis of silicon–perfluoropropenyl compounds 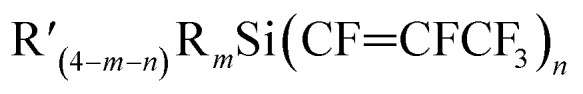, (*n* = 1, 2, 3, 4; *m* = 1, 2, 3) and R = Me, Et, Bu, ^i^Pr, Ph; R′ = ClCH_2_, ^*n*^Bu, Me, Ph.

Based on the previously published method by Brisdon *et al.*^9,10^ 1,1,3,3,3-pentafluoropropene (CF_3_CF=CFH) – known commercially as *Z*-HFC-1225ye – was used as a starting material to generate the intermediate *Z*-perfluoropropenyl lithium (CF_3_CF=CFLi), followed by reaction with 
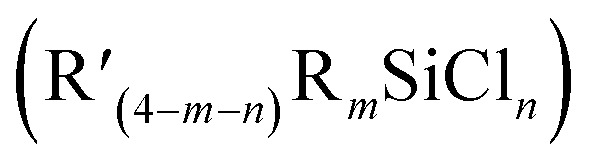
 to prepare 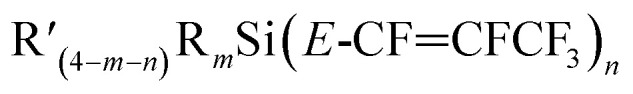, as outlined in Scheme 1.

The ^19^F{^1^H} spectra of all of the silicon–perfluoropropenyl compounds produced the anticipated results: 3 signals with a relative intensity ratio of 3 : 1 : 1, and correlated with the expectations of the perfluoropropenyl fragment. Similarly to the published main-group^9^ and transition-metal perfluoropropenyl complexes,^10^ the CF_3_ signal appeared between −65 ppm to −70 ppm with a higher intensity than F_*gem*_ and F_*trans*_ making its assignment straightforward.

In addition, the signal produced, on average, coupling constants of around 13 Hz between CF_3_ and F_*trans*_ and around 6 Hz between CF_3_ and F_*gem*_. The signals for F_*gem*_ and F_*trans*_ were observed around (−127 to −137) ppm and (−136 to −145) ppm, respectively, and displayed mutual coupling with the CF_3_ nuclei and each other. Interestingly, in the di-, tri- and tetra-perfluoropropenyl substituted compounds (Table 1), the CF_3_ signal was observed sometime as a doublet of doublets of doublets. The presence of an additional coupling to the CF_3_ group is thought to have occurred from a fluorine atom through space, or through the bonds, in addition to the coupling from F_*gem*_ and F_*trans*_. This was similar to the coupling patterns that had been observed for [(COD)Pt(*E*-CF=CFCF_3_)_2_].^10^ The ^19^F{^1^H} shows an instance of additional coupling in Si(*E*-CF=CFCF_3_)_4_ (a.11), wherein the CF_3_ signal was observed as a doublet of multiplets instead of a doublet of doublets as expected if coupling only occurred to F_*gem*_ and F_*trans*_.

The ^13^C{^1^H} NMR spectra of the perfluoropropenyl part of the compounds were as expected, (see ESI†) wherein the C_3_ signal was observed as a quartet of doublets of doublets due to the coupling to three equivalent F nuclei of the CF_3_ group through one bond, coupling to F_*trans*_ through two bonds and to F_*gem*_ through three bonds and the coupling constants were found to be *ca.* 270, 37, and 10 Hz respectively. The C_2_ signal appeared as a doublet of quartet of doublets due to the coupling to F_*trans*_, the three equivalent CF_3_ fluorines and F_*gem*_, and the coupling constants were found to be *ca.* 270, 39, and 20 Hz respectively. The C_1_ signal also appeared as a doublet of quartet of doublets, the coupling constants were found to be *ca.* 287, 4.7, and 1.7 Hz respectively.

The ^29^Si{^1^H} NMR spectra of the mono-perfluoropropenyl substituted compounds exhibited a significant coupling with F_*gem*_ nucleus, resulting in doublet splitting patterns, with coupling constant of between 20 to 30 Hz, which agrees with the coupling constant of the Si satellites in the ^19^F{H} spectrum of F_*gem*_. For comparison, di-, tri- and tetra-substituted-perfluoropropenyl compounds exhibited multiplet splitting patterns as expected.

**Fig. 2:**
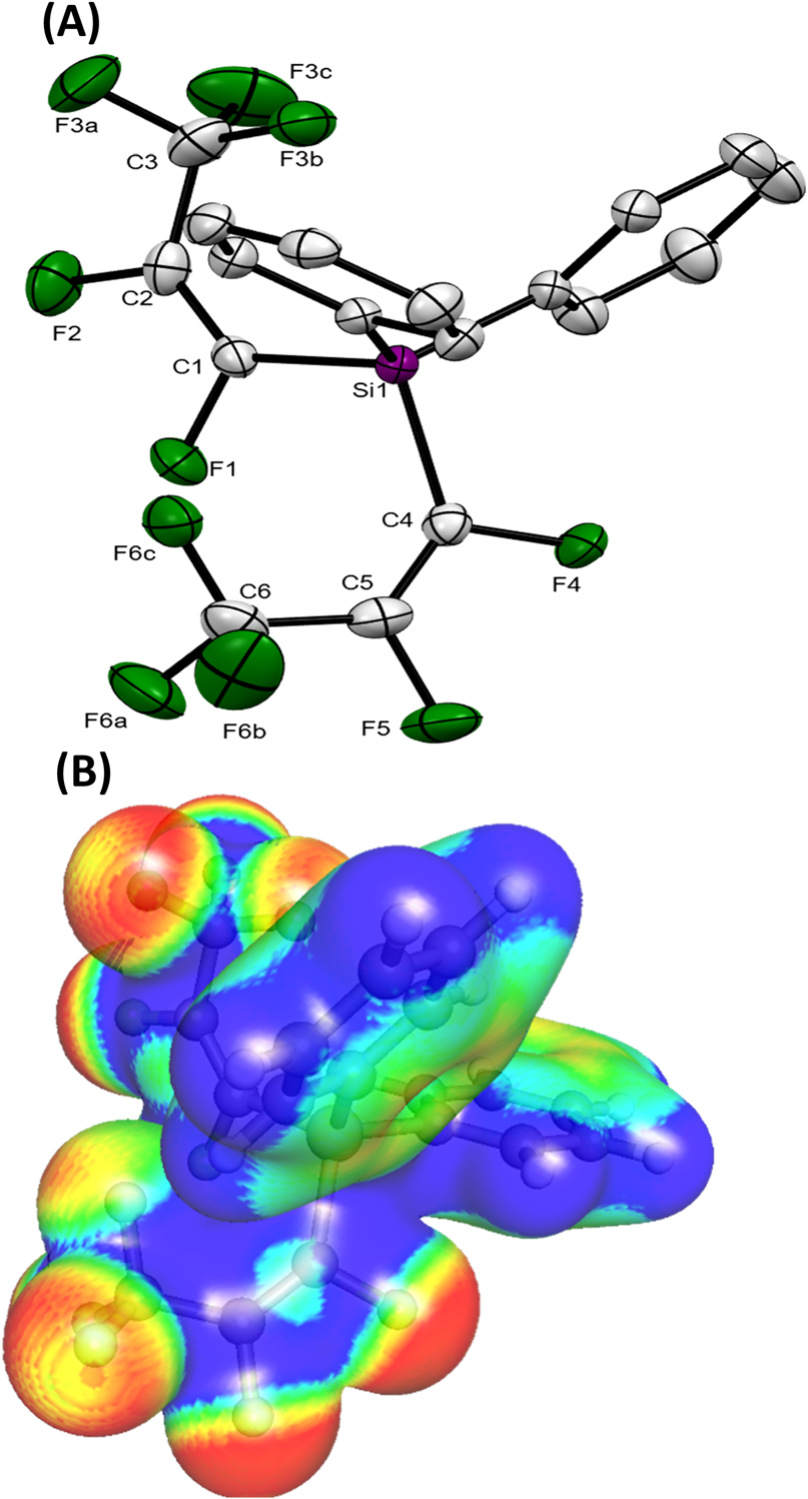
(A) ORTEP representation of the structure of (Ph)_2_Si(*E*-CF=CFCF_3_)_2_ (a.9), (hydrogen atoms omitted for clarity), thermal ellipsoids are shown at 50% probability level. (B) Electrostatic potential map colour coded on the charge density (isovalue 0.01) showing electron rich/deficient (red/blue) regions.

The majority of the silicon–perfluoropropenyl compounds were liquids, which limited the ability for structural characterisation by single crystal X-ray diffraction. However, (Ph)_2_Si(*E*-CF=CFCF_3_)_2_ (a.9) was a solid and attempts to grow single crystals were successful. The crystallographic data for the obtained crystal presented in Fig. 2 (see ESI, Tables S11 and S12†) confirmed that (Ph)_2_Si(*E*-CF=CFCF_3_)_2_ (a.9) was di-substituted, which correlated with the findings from the multinuclear NMR studies (Table 1). The bond lengths and angles for the perfluoropropenyl part of the compound showed similar data to the reported crystallographic values for the transition-metals perfluoropropenyl compounds.^9,10^

### X-ray crystallography and DFT studies

**Fig. 3:**
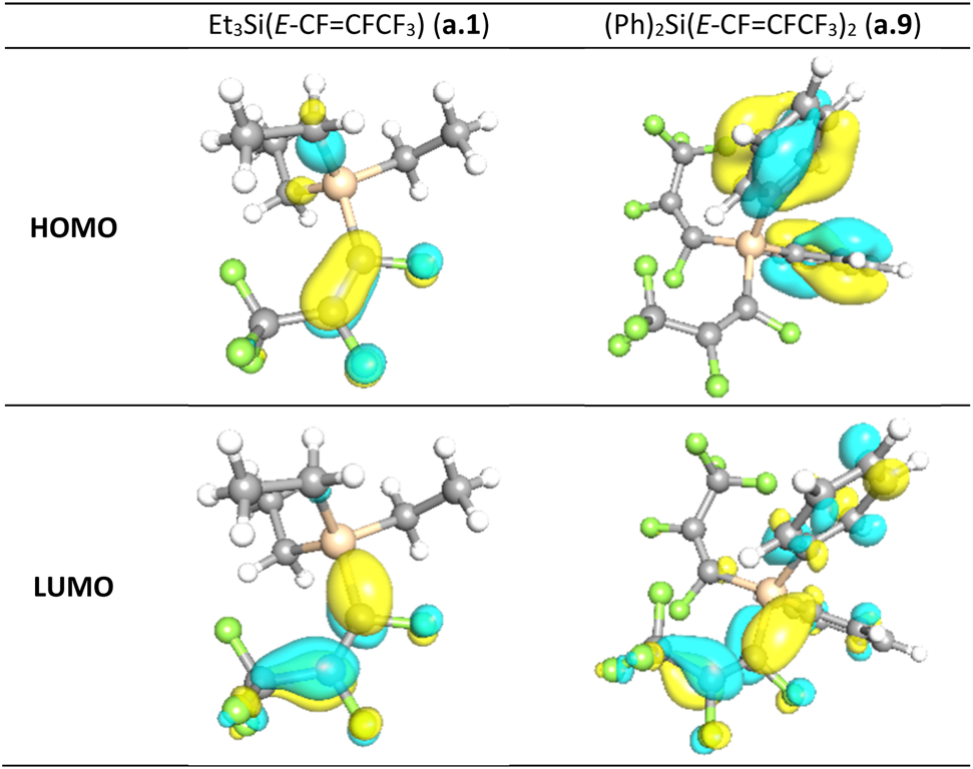
Visualization of HOMO and LUMO orbitals computed for Et_3_Si(*E*-CF=CFCF_3_) (a.1) and (Ph)_2_Si(*E*-CF=CFCF_3_)_2_ (a.9). Ball and stick representation of the structure: Si – orange, C – grey, F – green, H – white.

For all compounds, the geometry optimization and electronic structure calculations were performed with DFT. For Ph_2_Si(*E*-CF=CFCF_3_)_2_ (a.9), the reproduction of the observed solid-state distances by calculations of gas phase geometry using parameters that excluded diffuse functions from the basis set was imperfect, it was pleasing to see that many trends of the observed geometry were reproduced. As illustrated in Table 2, the differences between the observed bond lengths and calculated values were *ca.* 0.02 Å. The HOMO and LUMO (highest occupied molecular orbital and lowest unoccupied molecular orbital) orbitals are localized mainly on the carbons of the perfluoropropenyl group of the molecule in all cases except compounds with Ph group present, in which these orbitals are more spatially separated (see Fig. 3 and ESI-Table S10†). Thus, in the case of (Me)_2_PhSi(*E*-CF=CFCF_3_) (a.5), (Ph)_2_MeSi(*E*-CF=CFCF_3_) (a.6) and (Ph)_2_Si(*E*-CF=CFCF_3_)_2_ (a.9) HOMO is localized on the Ph group, whereas the LUMO mainly on the CF=CFCF_3_ part. The localization of the LUMO on the perfluoropropenyl group in all studied compounds indicates the electrophilic character of this group. Further, the calculations of the relative charges on the carbons of the perfluoropropenyl group were also performed. The positive charges on C_2_ were found to be higher than those on C_1_, (see Table 3). This suggested the likelihood for preferential nucleophilic attack at the C_2_ of the perfluoropropenyl group. This charge distribution appears to be consistent irrespective of the other groups coordinated to the silicon centre. For all of the compounds for which calculations were performed, as illustrated in Table 3, Me_2_Si(*E*-CF=CFCF_3_)_2_ (a.7), Ph_2_Si(*E*-CF=CFCF_3_)_2_ (a.9) and PhSi(*E*-CF=CFCF_3_)_3_ (a.10) the C_2_ centre is more positive than C_1_. The electrostatic potential maps computed for (a.1), (a.2), (a.3), (a.5), (a.6), (a.7), (a.8), and (a.9) confirm this trend (see ESI Table S13†).

**Table 2:** Selected bond lengths (Å) for (Ph)_2_Si(*E*-CF=CFCF_3_)_2_ (a.9) from the crystallographic data (solid phase) with estimated standard deviations in parentheses; and the calculated with DFT bond lengths (Å) for (Ph)_2_Si(*E*-CF=CFCF_3_)_2_ (a.9) in the gas phase

Solid phase		Gas phase	
Atoms	Bond length Å	Atoms	Bond length Å
Si_1_–C_1_	1.895(2)	Si_3_–C_26_	1.921
C_1_–C_2_	1.323(3)	C_26_–C_33_	1.317
C_2_–C_3_	1.491(4)	C_33_–C_35_	1.499
F_1_–C_1_	1.362(2)	F_34_–C_26_	1.341
F_2_–C_2_	1.342(3)	F_36_–C_33_	1.324
F_3a_—C_3_	1.330(3)	F_37_–C_35_	1.320
F_3b_—C_3_	1.326(3)	F_38_–C_35_	1.316
F_3c_—C_3_	1.319(3)	F_39_–C_35_	1.319
Si_1_–C_4_	1.906(2)	Si_3_–C_23_	1.925
C_4_–C_5_	1.319(3)	C_23_–C_24_	1.317
C_5_–C_6_	1.484(4)	C_24_–C_28_	1.497
F_4_–C_4_	1.365(3)	F_25_–C_23_	1.340
F_5_–C_5_	1.349(3)	F_27_–C_24_	1.323
F_6a_—C_6_	1.334(3)	F_29_–C_28_	1.320
F_6b_—C_6_	1.314(3)	F_31_–C_28_	1.316
F_6c_—C_6_	1.329(3)	F_30_–C_28_	1.319

**Table 3:** Calculated Mulliken charges for selected atoms in Me_2_Si(*E*-CF=CFCF_3_)_2_ (a.7), Ph_2_Si(*E*-CF=CFCF_3_)_2_ (a.9), and PhSi(*E*-CF=CFCF_3_)_3_ (a.10)

Atoms	(a.10)	(a.9)	(a.7)	
Si	0.707970	0.768787	0.452303	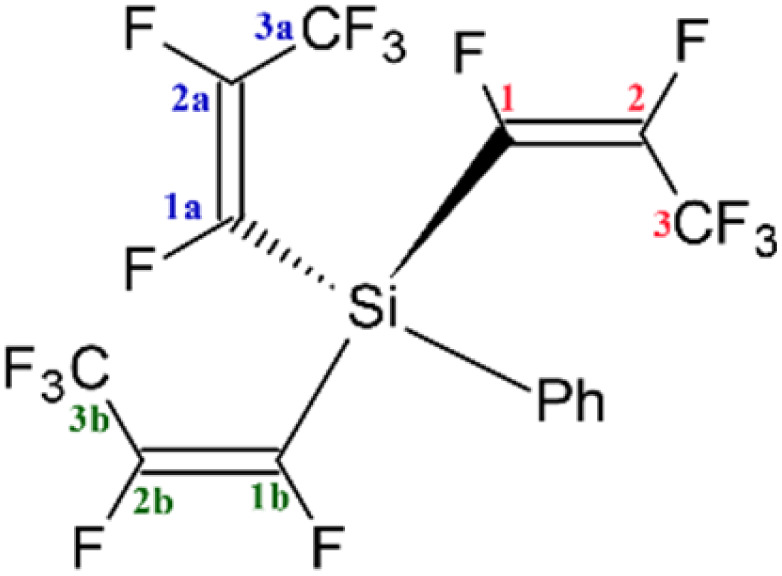
C_1_	0.157803	0.156063	0.157768	
C_2_	0.225322	0.253306	0.223625	
C_1a_	0.154999	0.157344	0.156894	
C_2a_	0.275160	0.259685	0.207578	
C_1b_	0.152841			
C_2b_	0.287702			

**Scheme 2:**
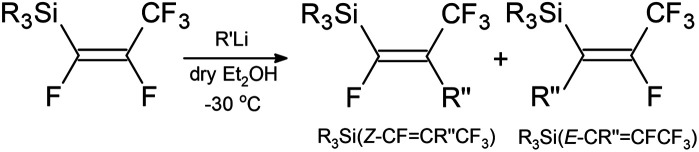
The general reaction of silicon–perfluoropropenyl compounds with R′′Li (R = Me, Et, ^*n*^Bu, ^i^Pr, Ph; R′′ = ^*n*^Bu, ^*t*^Bu, Me, Ph).

Next, calculations of thermodynamic reaction energies between 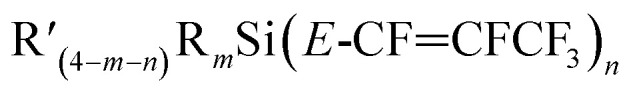 compounds and nucleophilic reagent R′′Li, according to reaction summarized in Scheme 2, were conducted. As an example, DFT reaction energetics for Et_3_Si(*E*-CF=CFCF_3_) (a.1) are presented in Table 4. For the energetics of the rest of the studied compounds in reaction with R′′Li see ESI-Table S10.† These calculations revealed that the nucleophilic attack at C_2_ position giving *Z*-isomer as a product is energetically more favourable in all studied cases. Generally, the preference of R′′ to attack the C_2_ position over C_1_ position increases in the order of: Ph < Me < ^*n*^Bu < ^*t*^Bu. Furthermore, the reactivity of nucleophiles in the reaction producing *Z*-isomer increases in the following order: Ph < ^*t*^Bu ≤ Me < ^*n*^Bu, except for (Me)_2_Si(*E*-CF=CFCF_3_)_2_ (a.7), where using ^*t*^BuLi is energetically the most favourable.

**Table 4:** DFT energetics [kcal mol^−1^] computed for the nucleophilic attack of R′Li at C_2_/C_1_ position of Et_3_Si(*E*-CF=CFCF_3_) (a.1) giving *Z*-/*E*-isomer, respectively

Nucleophile R′Li	Δ*E* [kcal mol^−1^] for product	
	R_3_Si(*Z*-CF=CR′CF_3_)_*n*_	R_3_Si(*E*-CR′=CFCF_3_)_*n*_
^*n*^BuLi	−76.35	−70.27
MeLi	−73.55	−67.31
^*t*^BuLi	−72.86	−59.34
PhLi	−69.99	−66.70

### C–F activation *via* nucleophilic attack

The outcome from DFT calculation suggests that C–F_*trans*_ bond has a higher tendency to be replaced than C–F_*gem*_ bond. This theory has been tested by treating the silicon–perfluoropropenyl compounds with different nucleophilic sources (^*n*^BuLi, ^*t*^BuLi, MeLi, and PhLi), as shown in Scheme 2. In most of the reactions studied (see Table 6), ^19^F{^1^H} spectra showed four peaks, and the *J* values confirmed the existence of a mixture of two compounds for the reaction between R_3_Si(*E*-CF=CFCF_3_) and the organolithium reagents. For example, the reaction of Et_3_Si(*E*-CF=CFCF_3_) (a.1) and ^*n*^BuLi, the ^19^F{^1^H} NMR spectrum showed a total of four peaks, two in the CF_3_ region and two others. Based on their integration values they could be divided into two sets of peaks with relative intensities of 3 : 1. For the first pair of peaks (*Z*-isomer in Scheme 2), a mutual *J* coupling of approximately 8 Hz was measured, while for the *E*-isomer, the mutual *J* coupling was slightly larger (around 10 Hz). In both cases the ^19^F and ^19^F{^1^H} NMR spectra exhibited the same splitting patterns, which excluded the probability of couplings to H. The ^29^Si{^1^H} NMR spectrum showed two peaks with similar intensity ratios as those found in the ^19^F{^1^H} spectra. Each signal was a doublet with *J* values of approximately 30 Hz and 9 Hz.

The elemental analysis data for the resulting mixture from treatment of Et_3_Si(*E*-CF=CFCF_3_) (a.1) with ^*n*^BuLi was C: 54.55% and H: 8.23%. These values are close to the values calculated for (a.1) in which one of the fluorines has been replaced by a ^*n*^Bu group, which are C: 54.92% and H: 8.51% (see Table 5). This suggests that the two compounds observed are isomers, rather than two different products.

**Table 5:** Elemental analysis of (a.1) and the outcome of reaction with RLi

Compound	Molecular formula	% weight (theory)	% weight (found)
(a.1)	C_9_H_15_F_5_Si	C, 43.89; H, 6.14	C, 43.20; H, 5.95
(a.1) + ^*n*^BuLi	C_13_H_24_F_4_Si	C, 54.90; H, 8.51	C, 54.55; H, 8.23
(a.1) + ^*t*^BuLi	C_13_H_24_F_4_Si	C, 54.90; H, 8.51	C, 54.89; H, 8.03
(a.1) + MeLi	C_10_H_18_F_4_Si	C, 49.56; H, 7.49	C, 49.73; H, 7.12
(a.1) + PhLi	C_15_H_20_F_4_Si	C, 59.19; H, 6.63	C, 59.63; H, 6.91

For some reactions the ^19^F{^1^H} and ^29^Si{^1^H} spectra confirmed that only one compound had been generated, as was the case for the reaction between Et_3_Si(*E*-CF=CFCF_3_) (a.1) and ^*t*^BuLi. In this case, the mutual F–F coupling in the ^19^F{^1^H} spectrum was around 7 Hz, while the Si–F coupling observed in the ^29^Si{^1^H} spectrum was 32.9 Hz. These values are similar to those observed for the more intense of the two sets of signals in the mixture that resulted from the reaction between Et_3_Si(*E*-CF=CFCF_3_) (a.1) and ^*n*^BuLi.

**Fig. 4:**
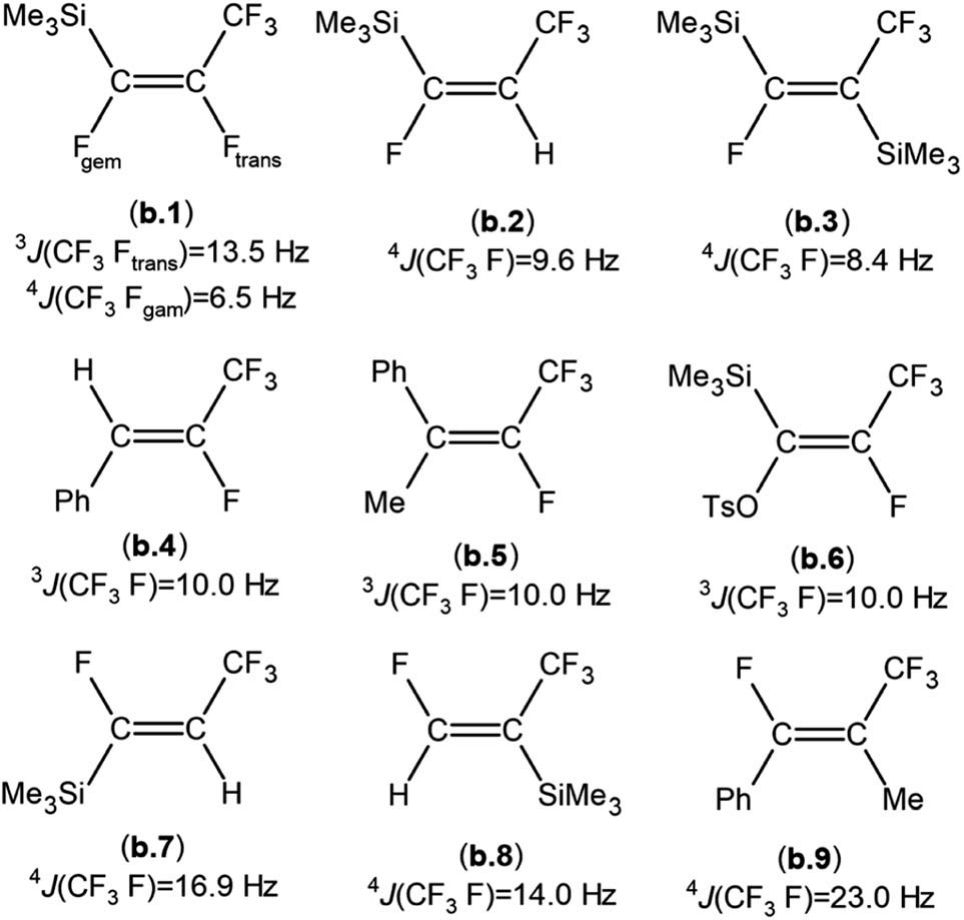
Some examples of published compounds formally derived from substitution of a fluorine atom from a sp^2^ hybridised carbon atom of a perfluoropropenyl group: (b.1),^1^ (b.2),^11^ (b.3),^11^ (b.4),^5^ (b.5),^6^ (b.6),^8^ (b.7),^11^ (b.8),^11^ (b.9),^5^ (TsO = CH_3_C_6_H_4_SO_2_).

**Fig. 5:**
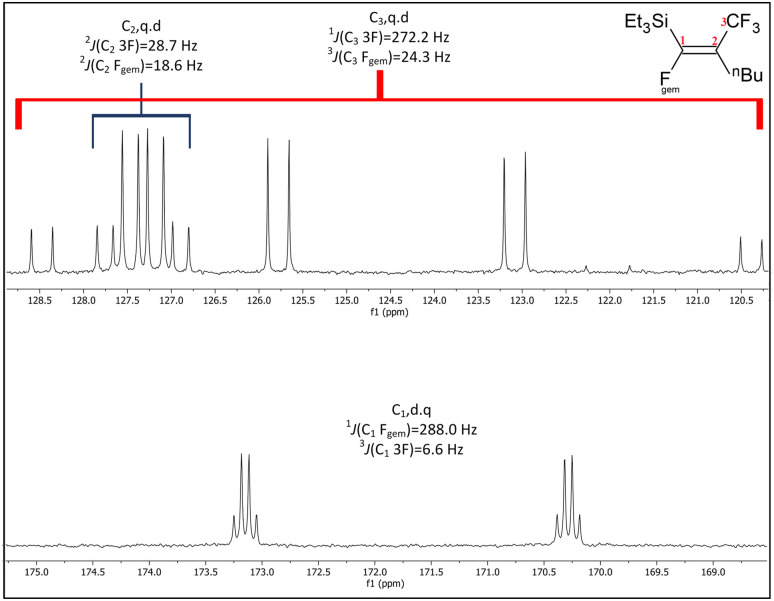
Expansions of C_1_ to C_3_ signals in the ^13^C{^1^H} NMR spectrum for the major product from the reaction of Et_3_Si(*E*-CF=CFCF_3_) (a.1) with ^*n*^BuLi, (100 MHz, CDCl_3_, 298 K).

**Fig. 6:**
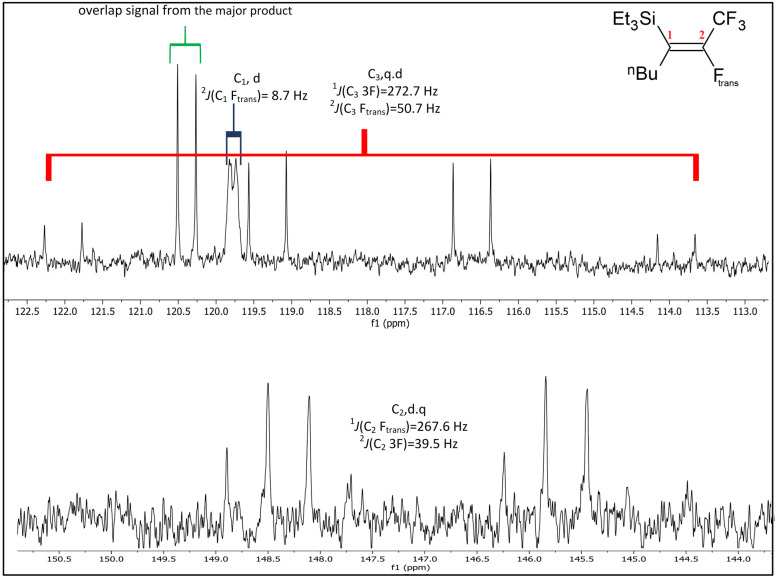
Expansions of C_1_ to C_3_ signals in the ^13^C{^1^H} NMR spectrum for the minor product from the reaction of Et_3_Si(*E*-CF=CFCF_3_) (a.1) with ^*n*^BuLi, (100 MHz, CDCl_3_, 298 K).

When compared to similar systems (see Fig. 4), a range of coupling constants between CF_3_ and F are observed, but generally the CF_3_–F_*trans*_ coupling constants are bigger (>10 Hz) than the coupling constants between CF_3_ and F_*gem*_ (<10 Hz). The ^4^*J*(CF_3_–F) coupling between substituents on the same side of the double bond are between 15–20 Hz. The ^19^F{^1^H} NMR data for the major product formed in the reaction of ^*n*^BuLi with Et_3_Si(*E*-CF=CFCF_3_) (a.1) and the only product formed when ^*t*^BuLi was used, had F–F coupling constants of *ca.* 8.0 and 7.0 Hz respectively. This suggests that the first compound in the mixture occurred as a result of the F_*trans*_ substitution by the ^*n*^Bu group leaving F_*gem*_ to couple with the CF_3_ signal. This substitution produced Et_3_Si(*Z*-CF=C^*n*^BuCF_3_) (12*Z*), see Scheme 2 and Table 6, which correlated with the ^29^Si{^1^H} NMR data, where the coupling between Si and F was approximately 30 Hz. This is similar to the coupling between Si and F_*gem*_ in Et_3_Si(*E*-CF=CFCF_3_) (a.1). On the other hand, the low abundance product was Et_3_Si(*E*-C^*n*^Bu=CFCF_3_) (12*E*) which likely results from substitution of F_*gem*_ with the ^*n*^Bu group. This is consistent with both the coupling between CF_3_ and F, which is *ca.* 11.0 Hz, in agreement with b.4,^5^b.5 ^6^ and b.6 ^8^ (see Fig. 4 and Table 6) and the smaller Si–F coupling since the fluorine is now further from the silicon centre. The fluorine NMR data also indicated that it was unlikely for the second compound to be formed with the *cis* geometry. The results of the ^13^C{^1^H} NMR spectra also correlate well with the suggested interpretation of the other NMR data. For the major product of the reaction involving ^*n*^BuLi, expansions of the signals for the perfluoropropenyl carbons are shown in Fig. 5, while the corresponding signals for the minor product are shown in Fig. 6. For the major species, the carbon of the CF_3_ couples to the other fluorine with *J* = 24.3 Hz, whereas in the minor product the coupling between the carbon nucleus of the CF_3_ and the other F is 50.7 Hz, which indicates that the CF_3_ is separated from the F by fewer bonds in the minor species (*E*-isomer) compared with the major product (*Z*-isomer). Similarly, the coupling between the carbon directly bonded to the unique fluorine atom exhibits a larger quartet coupling in *E*-isomer (39.5 Hz) than in *Z*-isomer (6.6 Hz).

**Table 6:** Summary of the results of reactions between 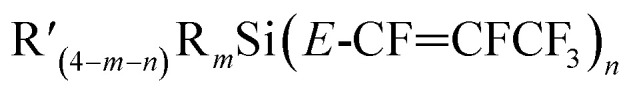 and nucleophilic sources with product ratio (given as %), and ^19^F{^1^H}, ^29^Si{^1^H} NMR data (CDCl_3_)

Reactant 1	Reactant 2	Ratio	Result	*δ* CF_3_	*δ* F	*δ* Si
Et_3_Si(*E*-CF=CFCF_3_) (a.1)	^*n*^BuLi	88	Et_3_Si(*Z*-CF=C^*n*^BuCF_3_) (12*Z*)	−60.28 ppm, d, ^4^*J* = 8.6 Hz	−99.31 ppm, q, ^4^*J* = 8.2 Hz	6.20 ppm, d, ^2^*J* = 31.2 Hz
		12	Et_3_Si(*E*-C^*n*^Bu=CFCF_3_) (12*E*)	−67.34 ppm, d, ^3^*J* = 10.6 Hz	−106.75 ppm, ^3^*J* = 10.9 Hz	4.96 ppm, d, ^3^*J* = 9.4 Hz
	^*t*^BuLi	100	Et_3_Si(*Z*-CF=C^*t*^BuCF_3_) (13*Z*)	−55.85 ppm, d, ^4^*J* = 7.4 Hz	−87.06 ppm, q, ^4^*J* = 7.1 Hz	8.70 ppm, d, ^2^*J* = 32.8 Hz
	MeLi	79	Et_3_Si(*Z*-CF=CMeCF_3_) (14*Z*)	−62.50 ppm, d, ^4^*J* = 8.6 Hz	−98.47 ppm, q, ^4^*J* = 8.7 Hz	6.15 ppm, d, ^2^*J* = 30.7 Hz
		21	Et_3_Si(*E*-CMe=CFCF_3_) (14*E*)	−67.07 ppm, d, ^3^*J* = 10.3 Hz	−105.47 ppm, q, ^3^*J* = 10.3 Hz	8.95 ppm, d, ^3^*J* = 6.8 Hz
	PhLi	77	Et_3_Si(*Z*-CF=CPhCF_3_) (15*Z*)	−58.61 ppm, d, ^4^*J* = 9.0 Hz	−93.33 ppm, q, ^4^*J* = 9.2 Hz	7.90 ppm, d, ^2^*J* = 30.3 Hz
		23	Et_3_Si(*E*-CPh=CFCF_3_) (15*E*)	−67.77 ppm, d, ^3^*J* = 10.6 Hz	−100.27 ppm, q, ^3^*J* = 10.5 Hz	5.73 ppm, d, ^3^*J* = 5.7 Hz
^*n*^Bu_3_Si(*E*-CF=CFCF_3_)(a.2)	^*n*^BuLi	88	^*n*^Bu_3_Si(*Z*-CF=C^*n*^BuCF_3_) (16*Z*)	−60.18 ppm, d, ^4^*J* = 8.4 Hz	−98.95 ppm, q, ^4^*J* = 8.5 Hz	2.1 ppm, d, ^2^*J* = 31.7 Hz
		12	^*n*^Bu_3_Si(*E*-C^*n*^Bu=CFCF_3_) (16*E*)	−67.22 ppm, d, ^3^*J* = 10.6 Hz	−106.90 ppm, q, ^3^*J* = 10.9 Hz	0.93 ppm, d, ^3^*J* = 9.3 Hz
	^*t*^BuLi	100	^*n*^Bu_3_Si(*Z*-CF=C^*t*^BuCF_3_) (17*Z*)	−55.55 ppm, d, ^4^*J* = 7.1 Hz	−86.66 ppm, q, ^3^*J* = 7.2 Hz	2.15 ppm, d, ^2^*J* = 31.1 Hz
	MeLi	83	^*n*^Bu_3_Si(*Z*-CF=CMeCF_3_) (18*Z*)	−62.42 ppm, d, ^4^*J* = 8.4 Hz	−98.11 ppm, q, ^4^*J* = 8.5 Hz	2.0 ppm, d, ^2^*J* = 31.0 Hz
		17	^*n*^Bu_3_Si(*E*-CMe=CFCF_3_) (18*E*)	−67.06 ppm, d, ^3^*J* = 10.6 Hz	−105.64 ppm, q, ^3^*J* = 10.5 Hz	0.90 ppm, d, ^3^*J* = 8.7 Hz
	PhLi	69	^*n*^Bu_3_Si(*Z*-CF=CPhCF_3_) (19*Z*)	−58.51 ppm, d, ^4^*J* = 9.1 Hz	−93.02 ppm, q, ^4^*J* = 9.0 Hz	3.71 ppm, d, ^2^*J* = 30.6 Hz
		31	^*n*^Bu_3_Si(*E*-CPh=CFCF_3_) (19*E*)	−67.67 ppm, d, ^3^*J* = 10.8 Hz	−100.34 ppm, q, ^3^*J* = 10.8 Hz	−0.01 ppm, d, ^3^*J* = 5.4 Hz
^*n*^BuMe_2_Si(*E*-CF=CFCF_3_) (a.4)	^*n*^BuLi	57	^*n*^BuMe_2_Si(*Z*-CF=C^*n*^BuCF_3_) (20*Z*)	−59.74 ppm, d, ^4^*J* = 8.5 Hz	−100.36 ppm, q, ^4^*J* = 8.6 Hz	3.71 ppm, d, ^2^*J* = 30.6 Hz
		43	^*n*^BuMe_2_Si(*E*-C^*n*^Bu=CFCF_3_) (20*E*)	−66.88 ppm, d, ^3^*J* = 10.5 Hz	−108.49 ppm, q, ^3^*J* = 10.4 Hz	−0.01 ppm, d, ^3^*J* = 5.4 Hz
	^*t*^BuLi	100	^*n*^BuMe_2_Si(*Z*-CF=C^*t*^BuCF_3_) (21*Z*)	−55.20 ppm, d, ^4^*J* = 7.3 Hz	−88.24 ppm, q, ^4^*J* = 7.1 Hz	−1.85 ppm, d, ^2^*J* = 37.2 Hz
	MeLi	40	^*n*^BuMe_2_Si(*Z*-CF=CMeCF_3_) (22*Z*)	−61.59 ppm, d, ^4^*J* = 8.3 Hz	−99.39 ppm, q, ^4^*J* = 8.5 Hz	−21.95 ppm, d, ^2^*J* = 37.0 Hz
		60	^*n*^BuMe_2_Si(*E*-CMe=CFCF_3_) (22*E*)	−66.65 ppm, d, ^3^*J* = 10.2 Hz	−107.12 ppm, q, ^3^*J* = 10.5 Hz	7.25 ppm, d, ^3^*J* = 5.6 Hz
Me_2_PhSi(*E*-CF=CFCF_3_) (a.5)	^*n*^BuLi	55	Me_2_PhSi(*Z*-CF=C^*n*^BuCF_3_) (23*Z*)	−59.38 ppm, d, ^4^*J* = 8.2 Hz	−99.54 ppm, q, ^4^*J* = 8.6 Hz	−7.7 ppm, d, ^2^*J* = 37.8 Hz
		45	Me_2_PhSi(*E*-C^*n*^Bu=CFCF_3_) (23*E*)	−66.48 ppm, d, ^3^*J* = 10.0 Hz	−107.00 ppm, q, ^3^*J* = 10.2 Hz	−6.22 ppm, d, ^3^*J* = 11.5 Hz
	^*t*^BuLi	62	Me_2_PhSi(*Z*-CF=C^*t*^BuCF_3_) (24*Z*)	−59.17 ppm, d, ^4^*J* = 8.2 Hz	−99.55 ppm, q, ^4^*J* = 9.0 Hz	−2.45 ppm, d, ^2^*J* = 38.0 Hz
		38	Me_2_PhSi(*E*-C^*t*^Bu=CFCF_3_) (24*E*)	−66.27 ppm, d, ^3^*J* = 10.3 Hz	−106.72 ppm, q, ^3^*J* = 10.9 Hz	−1.14 ppm, d, ^3^*J* = 6.6 Hz
	MeLi	72	Me_2_PhSi(*Z*-CF=CMeCF_3_) (25*Z*)	−60.17 ppm, d, ^4^*J* = 8.2 Hz	−98.95 ppm, q, ^4^*J* = 8.7 Hz	6.10 ppm, d, ^2^*J* = 37.4 Hz
		28	Me_2_PhSi(*E*-CMe=CFCF_3_) (25*E*)	−67.20 ppm, d, ^3^*J* = 10.5 Hz	−106.91 ppm, q, ^3^*J* = 10.9 Hz	1.88 ppm, d, ^3^*J* = 5.6 Hz
Ph_2_MeSi(*E*-CF=CFCF_3_) (a.6)	^*n*^BuLi	70	Ph_2_MeSi(*Z*-CF=C^*n*^BuCF_3_) (26*Z*)	−59.31 ppm, d, ^4^*J* = 8.3 Hz	−97.03 ppm, q, ^4^*J* = 8.5 Hz	−12.60 ppm, d, ^2^*J* = 38.0 Hz
		30	Ph_2_MeSi(*E*-C^*n*^Bu=CFCF_3_) (26*E*)	−66.65 ppm, d, ^3^*J* = 10.4 Hz	−105.47 ppm, q, ^3^*J* = 10.3 Hz	−9.34 ppm, d, ^3^*J* = 12.9 Hz
	^*t*^BuLi	100	Ph_2_MeSi(*Z*-CF=C^*t*^BuCF_3_) (27*Z*)	−54.77 ppm, d, ^4^*J* = 6.7 Hz	−84.52 ppm, q, ^4^*J* = 6.7 Hz	−10.03 ppm, d, ^2^*J* = 37.5 Hz
	MeLi	52	Ph_2_MeSi(*Z*-CF=CMeCF_3_) (28*Z*)	−61.46 ppm, d, ^4^*J* = 7.9 Hz	−96.13 ppm, q, ^4^*J* = 7.7 Hz	−21.91 ppm, d, ^2^*J* = 37.3 Hz
		48	Ph_2_MeSi(*E*-CMe=CFCF_3_) (28*E*)	−66.37 ppm, d, ^3^*J* = 10.4 Hz	−103.41 ppm, q, ^3^*J* = 10.7 Hz	−10.99 ppm, d, ^3^*J* = 5.3 Hz
	PhLi	100	Ph_2_MeSi(*Z*-CF=CPhCF_3_) (29*Z*)	−61.13 ppm, d, ^4^*J* = 9.6 Hz	−79.91 ppm, q, ^4^*J* = 9.5 Hz	−22.29 ppm, d, ^2^*J* = 36.3 Hz
Me_2_Si(*E*-CF=CFCF_3_)_2_ (a.7)	^*n*^BuLi	50	Me_2_Si(*Z*-CF=C^*n*^BuCF_3_)_2_ (30*Z*)	−59.71 ppm, d, ^4^*J* = 8.4 Hz	−100.32 ppm, q, ^3^*J* = 8.5 Hz	−21.93 ppm, m
		50	Me_2_Si(*E*-C^*n*^Bu=CFCF_3_)_2_ (30*E*)	−66.79 ppm, d, ^3^*J* = 10.5 Hz	−108.51 ppm, q, ^4^*J* = 10.0 Hz	−0.57 ppm, m
	^*t*^BuLi	100	Me_2_Si(*Z*-C^*t*^Bu=CFCF_3_)_2_ (31*Z*)	−55.18 ppm, d, ^4^*J* = 7.2 Hz	−88.23 ppm, q, ^4^*J* = 7.5 Hz	1.80 ppm, m
	MeLi	40	Me_2_Si(*Z*-CMe=CFCF_3_)_2_ (32*Z*)	−62.05 ppm, d, ^4^*J* = 8.6 Hz	−99.44 ppm, q, ^4^*J* = 8.5 Hz	−22.76 ppm, m
		60	Me_2_Si(*E*-CF=CMeCF_3_)_2_ (32*E*)	−66.75 ppm, d, ^3^*J* = 10.3 Hz	−107.19 ppm, q, ^3^*J* = 10.4 Hz	−21.94 ppm, m
^i^Pr_2_Si(*E*-CF=CFCF_3_)_2_ (a.8)	^*n*^BuLi	100	^i^Pr_2_Si(*Z*-CF=C^*n*^BuCF_3_)_2_ (33*Z*)	−61.98 ppm, d, ^4^*J* = 8.6 Hz	−101.26 ppm, q, ^4^*J* = 8.4 Hz	−3.27 ppm, m
	^*t*^BuLi	100	^i^Pr_2_Si(*Z*-CF=C^*t*^BuCF_3_)_2_ (34*Z*)	−56.61 ppm, d, ^4^*J* = 6.5 Hz	−89.0 ppm, q, ^4^*J* = 5.9 Hz	−12.80 ppm, m
	MeLi	100	^i^Pr_2_Si(*Z*-CF=CMeCF_3_)_2_ (35*Z*)	−67.53 ppm, m	−139.77 ppm, m	−13.16 ppm, m
Ph_2_Si(*E*-CF=CFCF_3_)_2_ (a.9)	^*n*^BuLi	77	Ph_2_Si(*Z*-CF=C^*n*^BuCF_3_)_2_ (36*Z*)	−59.93 ppm, d, ^4^*J* = 7.7 Hz	−97.61 ppm, q, ^4^*J* = 8.0 Hz	−11.95 ppm, m
		23	Ph_2_Si(*E*-C^*n*^Bu=CFCF_3_)_2_ (36*E*)	−67.02 ppm, d, ^3^*J* = 10.5 Hz	−105.09 ppm, q, ^3^*J* = 10.8 Hz	−9.25 ppm, m
	PhLi	100	Ph_2_Si(*Z*-CF=CPhCF_3_)_2_ (37*Z*)	−57.84 ppm, d, ^4^*J* = 8.4 Hz	−90.35 ppm, q, ^4^*J* = 8.8 Hz	−21.80 ppm, m

**Fig. 7:**
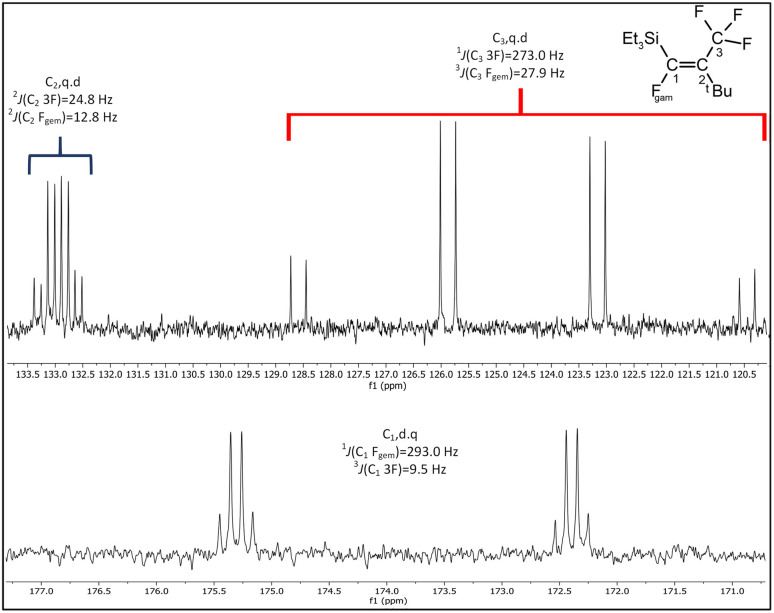
Expansions of C_1_ to C_3_ signals in the ^13^C{^1^H} NMR spectrum for the product from the reaction of Et_3_Si(*E*-CF=CFCF_3_) (a.1) with ^*t*^BuLi, (100 MHz, CDCl_3_, 298 K).

The ^13^C{^1^H} NMR spectrum for the single product formed in the reaction with ^*t*^BuLi, shown in Fig. 7, is very similar to that observed for *Z*-isomer, both in terms of the *J* coupling and splitting patterns. This suggests that the only product formed when using ^*t*^BuLi is (Et)_3_Si(*Z*-CF=C^*t*^BuCF_3_) (13*Z*). According to the DFT study of Ph_2_Si(*E*-CF=CFCF_3_)_2_, of the carbons in the perfluoropropenyl group C_2_ is energetically the most likely site for attack by the incoming nucleophile. Therefore, substitution of F_*trans*_ is the most likely result of nucleophilic attack, while attack at C_1_ to give the F_*gem*_ substituted compound is less favoured. This is consistent with the observation of a small amount of (Et)_3_Si(*E*-C(^*n*^Bu)=CFCF_3_) (13*E*). However, in case of bigger group such as ^*t*^Bu,^10^ the steric hindrance prevents any attack on C_1_.

In Table 6, a summary of all successful attempts to substitute one fluorine atom with an organic group, by reaction with organolithium compounds is listed. PhSi(*E*-CF=CFCF_3_)_3_ (a.10) and Si(*E*-CF=CFCF_3_)_4_ (a.11) were found not to result in substitution and therefore characterization was not possible. In these cases treatment with ^*n*^BuLi and ^*t*^BuLi resulted in ^19^F{^1^H} NMR spectra that showed a number of signals in the CF_3_ area, which were no longer present after work up of the reaction. Reactions with MeLi and PhLi resulted in ^19^F{^1^H} NMR spectra which showed that there was no reaction. The reason that these two substrates reacted differently could be based on steric or electronic factors, or both. For example the large size of the ^*t*^Bu group could affect the ability to add to the presumably already sterically crowded PhSi(*E*-CF=CFCF_3_)_3_ (a.10) and Si(*E*-CF=CFCF_3_)_4_ (a.11) molecules, while MeLi and PhLi are considered to be less reactive compared to ^*n*^BuLi and ^*t*^BuLi, consistent with DFT calculations. Moreover, the presence of a large number of sterically demanding perfluoropropenyl groups attached to the silicon centre make these substitution reactions less likely, instead allowing for alternative reactions, that result in breaking the Si perfluoropropenyl bond and producing small volatile fluorocarbon molecules which could be easily removed on work up.

#### Reaction with ^*n*^BuLi

The substitute of one fluorine atom of the perfluoropropenyl-containing silicon compounds with the ^*n*^Bu group using ^*n*^BuLi resulted in a mixture of products 
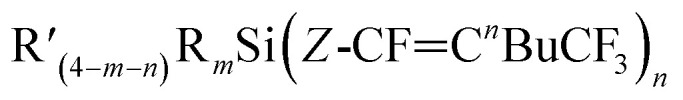
 and 
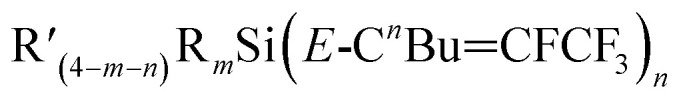
 in varying proportions. Most substrates resulted in the major products being the *Z*-isomers, such as was the case for (Et)_3_Si(*E*-CF=CFCF_3_) (a.1), (^*n*^Bu)_3_Si(*E*-CF=CFCF_3_) (a.2), (Ph)_2_MeSi(*E*-CF=CFCF_3_) (a.6) and (Ph)_2_Si(*E*-CF=CFCF_3_)_2_ (a.9). However, for ^*n*^Bu(Me)_2_Si(*E*-CF=CFCF_3_) (a.4) and (Me)_2_Si(*E*-CF=CFCF_3_)_2_ (a.7) similar proportions of the *E*- and *Z*-isomeric products were formed. By contrast, (^i^Pr)_2_Si(*E*-CF=CFCF_3_)_2_ (a.8) gave exclusively the *Z* isomer (^i^Pr)_2_Si(*Z*-CFC^*n*^BuCF_3_)_2_ (33*Z*). This variance in the proportion of *E* and *Z* isomers formed could be due to steric hindrance due to the sizes of the R groups attached to Si. For example, in the case of the most sterically demanding substituent, ^i^Pr, it was found that (^i^Pr)_2_Si(*E*-CF=CFCF_3_)_2_ (a.8) gave only the isomer (^i^Pr)_2_Si(*Z*-CF=C^*n*^BuCF_3_)_2_ (33*Z*). As the groups become smaller the proportion of *Z* isomer decreases and the *E*-isomeric product increases. For example: (^i^Pr)_2_Si(*E*-CF=CFCF_3_)_2_ (a.8) gave 100% of the *Z*-isomeric product, (Ph)_2_Si(*E*-CF=CFCF_3_)_2_ (a.9) gave 77% *Z*-isomer and (Me)_2_Si(*E*-CF=CFCF_3_)_2_ (a.7) 50% *Z*-isomer.

#### Reaction with ^*t*^BuLi

Given the above argument it would be anticipated that increasing the size of the incoming nucleophile is most likely to result in more production of the *Z*-isomer since the size of the ^*t*^Bu group limits the possibility of generating the *E*-isomer by replacing F_*gem*_ on C_1_. In the cases of the reactions of ^*t*^BuLi with (Et)_3_Si(*E*-CF=CFCF_3_) (a.1), (^*n*^Bu)_3_Si(*E*-CF=CFCF_3_) (a.2), (^*n*^Bu)Me_2_Si(*E*-CF=CFCF_3_) (a.4) (Ph)_2_MeSi(*E*-CF=CFCF_3_) (a.6), (Me)_2_Si(*E*-CF=CFCF_3_)_2_ (a.7), and (^i^Pr)_2_Si(*E*-CF=CFCF_3_)_2_ (a.8) all reactions resulted in exclusive formation of the *Z*-isomeric product. However, based on the NMR data reaction with (Me)_2_PhSi(*E*-CF=CFCF_3_) (a.5) gave a mixture of both (Me)_2_PhSi(*Z*-CF=C^*t*^BuCF_3_) (24*Z*) and (Me)_2_PhSi(*E*-C^*t*^Bu=CFCF_3_) (24*E*) in the ratio 62 : 38. In the case of the reaction of ^*t*^BuLi with (Ph)_2_Si(*E*-CF=CFCF_3_)_2_ (a.9) the ^19^F{^1^H} NMR data suggested fragmentation, due to the observation of many signals around the CF_3_ region in the ^19^F{^1^H} NMR spectrum of the crude reaction sample. However, these signals disappeared after the reaction had been worked up and it is suggested that they are therefore small volatile fluorocarbon species.

#### Reaction with MeLi

When the nucleophilic substitution reactions were performed with a much smaller nucleophile, such as MeLi, a mixture of *E*- and *Z*-isomeric products was always formed. Similar to reaction with ^*n*^BuLi, the reactions involving (Et)_3_Si(*E*-CF=CFCF_3_) (a.1) and (^*n*^Bu)_3_Si(*E*-CF=CFCF_3_) (a.2) gave a mixture of both products with a high proportion of *Z*-isomers. Smaller differences in the proportions of *E*- and *Z*-isomers were found in the mixtures that came from reacting MeLi with ^*n*^Bu(Me)_2_Si(*E*-CF=CFCF_3_) (a.4), (Ph)_2_MeSi(*E*-CF=CFCF_3_) (a.6) and (Me)_2_Si(*E*-CF=CFCF_3_)_2_ (a.7). However, by analysis of the ^19^F{^1^H} NMR spectrum, only for (^i^Pr)_2_Si(*E*-CF=CFCF_3_)_2_ (a.8) was 100% of the *Z* product obtained, (^i^Pr)_2_Si(*Z*-CF=CMeCF_3_)_2_ (25*Z*). Finally, (Ph)_2_Si(*E*-CF=CFCF_3_)_2_ (a.9) did not show any reaction, even after extending the reaction time to 5 days and the amount of MeLi added has been increased.

#### Reaction with PhLi

When using phenyllithium, like with ^*n*^BuLi and MeLi, (Et)_3_Si(*E*-CF=CFCF_3_) (a.1) and (^*n*^Bu)_3_Si(*E*-CF=CFCF_3_) (a.2) gave a mixture of the two isomeric products, with a high proportion of the *Z*-isomers. The reactions with (Ph)_2_MeSi(*E*-CF=CFCF_3_) (a.6) and (Ph)_2_Si(*E*-CF=CFCF_3_)_2_ (a.9) gave the single *Z*-isomer exclusively. Unfortunately, the substitution of F with the Ph group was unsuccessful for ^*n*^Bu(Me)_2_Si(*E*-CF=CFCF_3_) (a.4), (Me)_2_PhSi(*E*-CF=CFCF_3_) (a.5), (Me)_2_Si(*E*-CF=CFCF_3_)_2_ (a.7) and (^i^Pr)_2_Si(*E*-CF=CFCF_3_)_2_ (a.8) according to multinuclear NMR spectroscopy.

## Experimental

### Materials and methods

All reagents and solvents were purchased from Sigma-Aldrich (purity 97–98%) and used without purification. Non-chlorinated solvents were dried over sodium wire for at least 24 h prior to use. *Z*-HFC-1225ye was kindly donated by Mexichem Fluor. NMR spectra were recorded at 20 °C on a Bruker Avance III 400 MHz spectrometer operating at 400.00, 100.61, 376.46, and 79 MHz for ^1^H, ^13^C, ^19^F, and ^29^Si respectively using CDCl_3_ as solvent. Chemical shift values are quoted relative to TMS and CFCl_3_ in parts per million (ppm) on the *δ* scale and coupling constant (*J*) values are reported in Hz. Elemental analysis was conducted by the University of Manchester's School of Chemistry Micro-Analytical service. Single crystal was grown by slow evaporation of a chloroform solution and X-ray structures were obtained using SuperNova diffractometers using Mo Kα radiation (*λ* = 0.71073 Å). All the raw data frames were reduced and corrections were applied for Lorentz, polarisation and absorption using the multi-scan methods with CrysAlisPro.^12^

### Computational methods

The X-ray structural data were solved by direct methods, with full-matrix least-squares refinement of *F*^2^ using: Olex2,^13^ Shelx^14^ and Shelxtl^15^ programs. Mercury^16^ was used to generate the graphical representations. The geometry of Ph_2_Si(*E*-CF=CFCF_3_)_2_ (a.9) was optimised using hybrid Density Functional Theory (DFT) at the B3LYP/6-31G(d,p) level;^17,18^ using the GAMESS software^19^ to calculate the bond lengths of (Ph)_2_Si(*E*-CF=CFCF_3_)_2_ (a.9) in gas phase and the Mulliken charges for Me_2_Si(*E*-CF=CFCF_3_)_2_ (a.7), Ph_2_Si(*E*-CF=CFCF_3_)_2_ (a.9), PhSi(*E*-CF=CFCF_3_)_3_ (a.10), and Si(*E*-CF=CFCF_3_)_4_ (a.11). The electronic structure of (a.1, a.2, a.4, a.5, a.6, a.7, a.8, a.9) compounds and reaction energetics for nucleophilic attack was obtained with B3-LYP/TZVPP^20^ using TURBOMOLE V7.3 2018 suite of quantum chemical programs.^21^

### Synthesis of silicon–perfluoropropenyl compounds 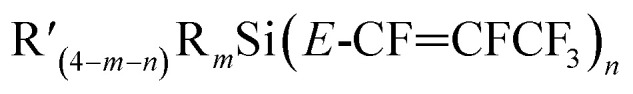


Was prepared with same procedure described in^10^ but on different scales (see ESI, Scheme S1†). In a three-necked round-bottom flask under a positive pressure of nitrogen cooled to between −75 to −80 °C were placed dry diethyl ether (150 mL) and one equivalent of *Z*-HFC-1225ye. One equivalent of ^*n*^BuLi (2.5 M solution in hexanes) was added slowly so as to maintain the temperature below −78 °C. The solution was stirred for 1 h to ensure formation of perfluoropropenyl lithium. In the next step, a solution of the appropriate silicon-halide was added slowly. The mixture was left to stir and warm slowly to room temperature overnight. Hexane (25 mL) was added to the reaction mixture and the resulting solution was filtered through a pad of Celite, and solvent was removed using a rotary evaporator.

### Reactions between silicon–perfluoropropenyl compounds 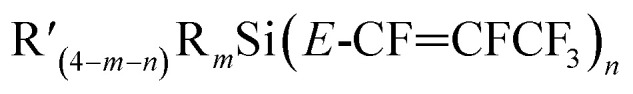 and nucleophilic sources

A solution of dry THF (150 mL) and 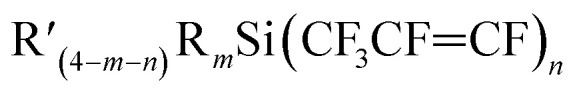 was placed in a three-necked round-bottom flask under a positive pressure of nitrogen. The solution was cooled to −30 °C, and then RLi (solution in hexanes) was added slowly. The mixture was slowly warmed to room temperature and left stirring for 24 hours. The reaction was worked up with hexanes (10 mL), followed by filtration through Celite, and solvent was removed using a rotary evaporator (see ESI, Scheme S2†).

## Conclusions

Derived from (HFC-1225 ye), eleven new and stable silicon–perfluoropropenyl compounds have been successfully prepared, and fully characterised by multinuclear NMR spectroscopy. The compounds formed are generally liquids at room temperature, except Ph_2_Si(*E*-CF=CFCF_3_)_2_ (a.9) which was solid and structural confirmation was obtained by X-ray diffraction studies.

The investigation of silicon–perfluoropropenyl compounds was extended to study the substitution reactions using a wide range of organolithium nucleophilic sources: ^*n*^BuLi, ^*t*^BuLi, MeLi, and PhLi, leading to twenty-six new compounds. Two types of products were identified: one where carbolithiation had occurred at C_1_ and one at C_2_, leading to two isomers of the 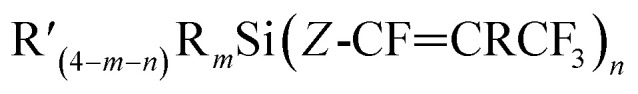 and 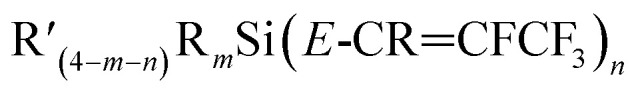 formula, respectively. The outcomes of these reactions were rationalised based on steric arguments. Bulky groups around the silicon centre or in the incoming nucleophile (*e.g.*^*n*^BuLi *vs.*^*t*^BuLi) led to a greater proportion of the *Z*-isomer. Due to uneven charges on the carbons of the pentafluoropropene group, where C_2_ attached to F_*trans*_ has higher positive charge than C_1_ attached to F_*gem*_, the nucleophilic attack preferred C_2_–F_*trans*_ to generate *Z*-isomer. The calculated reaction energetics between silicon–perfluoropropenyl compounds and organolithium reagents, confirmed that the *Z*-isomer is energetically more favoured product.

## Author contributions

L. Alluhaibi: conceptualization, investigation, methodology, writing – original draft, and writing – review & editing; A. Brisdon: supervision; S. Klejna: investigation, visualization, writing – review & editing; A. Muneer: formal analysis.

## Conflicts of interest

The authors declare there are no conflicts of interest.

## Supplementary Material

RA-013-D3RA01353G-s001

RA-013-D3RA01353G-s002
