# Supplementary material for: Synthesis and characterisation of new silicon–perfluoropropenyl compounds
Source: RSC Adv. 2023 May 3;13(20):13547–55. doi: 10.1039/d3ra01353g (PMC10154947; doi:10.1039/d3ra01353g)
Supplement: RA-013-D3RA01353G-s001 [file RA-013-D3RA01353G-s001.pdf]

Electronic Supplementary Material (ESI) for RSC Advances.
This journal is © The Royal Society of Chemistry 2023

Supplementary Data

Synthesis and Characterisation of New Silicon-Perfluoropropenyl Compounds

Lulu Alluhaibi,*[a] Alan Brisdon,[b] Sylwia Klejna[a] and Abeer Muneer[b]
[a].Academic Centre for Materials and Nanotechnology, AGH University of Science and Technology, 30-055 Kraków, Poland.
[b].School of Chemistry, The University of Manchester, Manchester M13 9PL, UK

Contents

General methods 2

1. Synthetic Reactions 2

1-1. General Synthesis Method of $R_{4-n}Si(E\text{-}CF{=}CFCF_3)_n$ (G.P1) 2
1-1-1. Synthesis of $(Et)_3Si(E\text{-}CF{=}CFCF_3)$ (a.1) 3
1-1-2. Synthesis of $^nBu_3Si(E\text{-}CF{=}CFCF_3)$ (a.2) 3
1-1-3. Synthesis of $ClCH_2(Me)_2Si(E\text{-}CF{=}CFCF_3)$ (a.3) 3
1-1-4. Synthesis of $^nBu(Me)_2Si(E\text{-}CF{=}CFCF_3)$ (a.4) 3
1-1-5. Synthesis of $(Me)_2PhSi(E\text{-}CF{=}CFCF_3)$ (a.5) 4
1-1-6. Synthesis of $(Ph)_2MeSi(E\text{-}CF{=}CFCF_3)$ (a.6) 4
1-1-7. Synthesis of $(Me)_2Si(E\text{-}CF{=}CFCF_3)_2$ (a.7) 4
1-1-8. Synthesis of $(^iPr)_2Si(E\text{-}CF{=}CFCF_3)_2$ (a.8) 4
1-1-9. Synthesis of $(Ph)_2Si(E\text{-}CF{=}CFCF_3)_2$ (a.9) 4
1-1-10. Synthesis of $PhSi(E\text{-}CF{=}CFCF_3)_3$ (a.10) 5
1-1-11. Synthesis of $Si(E\text{-}CF{=}CFCF_3)_4$ (a.11) 5

2. $R_{4-n}Si(E\text{-}CF{=}CFCF_3)_n$ Reactions 5

2-1. General Substitution Reactions (G.P2) 5
2-1-1. Reaction between $Et_3Si(E\text{-}CF{=}CFCF_3)$ (a.1) and ^nBuLi 5
2-1-2. Reaction between $Et_3Si(E\text{-}CF{=}CFCF_3)$ (a.1) and ^tBuLi 6
2-1-3. Reaction between $Et_3Si(E\text{-}CF{=}CFCF_3)$ (a.1) and MeLi 6
2-1-4. Reaction between $Et_3Si(E\text{-}CF{=}CFCF_3)$ (a.1) and PhLi 6
2-1-5. Reaction between $^nBu_3Si(E\text{-}CF{=}CFCF_3)$ (a.2) and ^nBuLi 7
2-1-6. Reaction between $^nBu_3Si(E\text{-}CF{=}CFCF_3)$ (a.2) and ^tBuLi 7
2-1-7. Reaction between $^nBu_3Si(E\text{-}CF{=}CFCF_3)$ (a.2) and MeLi 7
2-1-8. Reaction between $^nBu_3Si(E\text{-}CF{=}CFCF_3)$ (a.2) and PhLi 8
2-1-9. Reaction between $^nBu(Me)_2Si(E\text{-}CF{=}CFCF_3)$ (a.4) and ^nBuLi 8
2-1-10. Reaction between $^nBu(Me)_2Si(E\text{-}CF{=}CFCF_3)$ (a.4) and ^tBuLi 9
2-1-11. Reaction between $^nBu(Me)_2Si(E\text{-}CF{=}CFCF_3)$ (a.4) and MeLi 9
2-1-12. Reaction between $Me_2PhSi(E\text{-}CF{=}CFCF_3)$ (a.5) and ^nBuLi 9
2-1-13. Reaction between $Me_2PhSi(CF{=}CFCF_3)$ (a.5) and ^tBuLi 10
2-1-14. Reaction between $Me_2PhSi(E\text{-}CF{=}CFCF_3)$ (a.5) and MeLi 10
2-1-15. Reaction between $Ph_2MeSi(E\text{-}CF{=}CFCF_3)$ (a.6) and ^nBuLi 10
2-1-16. Reaction between $Ph_2MeSi(E\text{-}CF{=}CFCF_3)$ (a.6) and ^tBuLi 11

2-1-17. Reaction between $Ph_2MeSi(E\text{-}CF{=}CFCF_3)$ (3.6) and MeLi 11
2-1-18. Reaction between $Ph_2MeSi(E\text{-}CF{=}CFCF_3)$ (a.6) and PhLi 11
2-1-19. Reaction between $Me_2Si(E\text{-}CF{=}CFCF_3)_2$ (a.7) and ^{n}BuLi 11
2-1-20. Reaction between $Me_2Si(E\text{-}CF{=}CFCF_3)_2$ (a.7) and ^{t}BuLi 12
2-1-21. Reaction between $Me_2Si(E\text{-}CF{=}CFCF_3)_2$ (a.7) and MeLi 12
2-1-22. Reaction between $^{i}Pr_2Si(E\text{-}CF{=}CFCF_3)_2$ (a.8) and ^{n}BuLi..12
2-1-23. Reaction between $^{i}Pr_2Si(E\text{-}CF{=}CFCF_3)_2$ (a.8) and ^{t}BuLi 13
2-1-24. Reaction between $^{i}Pr_2Si(E\text{-}CF{=}CFCF_3)_2$ (a.8) and MeLi 13
2-1-25. Reaction between $Ph_2Si(E\text{-}CF{=}CFCF_3)_2$ (a.9) and ^{n}BuLi..13
2-1-26. Reaction between $Ph_2Si(E\text{-}CF{=}CFCF_3)_2$ (a.9) and PhLi 13

3. DFT calculations 14

3.1 Calculated Mulliken Charges 14
3.1.1 $(Me)_2Si(E\text{-}CF{=}CFCF_3)_2$ (a.5) 14
3.1.2 $(Ph)_2Si(E\text{-}CF{=}CFCF_3)_2$ (a.9) 16
3.1.3 $(Ph)Si(E\text{-}CF{=}CFCF3)_3$ (a.10) 18

3.2 Electronic structure and DFT reaction energetics for substitution reaction with nuclephilic sources 21

4. Crystallographic data 23

5.References 25

General methods

1. Synthetic Reactions

1-1. General Synthesis Method of $R_{4-n}Si(E\text{-}CF{=}CFCF_3)_n$ (G.P1)

In all the synthetic reactions, $LiCF{=}CFCF_3$ was prepared following the same procedure described below but on different scales. In a three-necked round-bottom flask under a positive pressure of nitrogen cooled to between −75 to −80 °C were placed dry diethyl ether (150 mL) and one equivalent of *Z*-HFC-1225ye. One equivalent of ^{n}BuLi (2.5 M solution in hexanes) was added slowly so as to maintain the temperature below −78 °C. The solution was stirred for 1 h to ensure formation of perfluoropropenyl lithium. In the next step, a solution of the appropriate silicon-halide was added slowly. The mixture was left to stir and warm slowly to room temperature overnight. Hexane (25 mL) was added to the reaction mixture and the resulting solution was filtered through a pad of celite, and solvent was removed using a rotary evaporator.

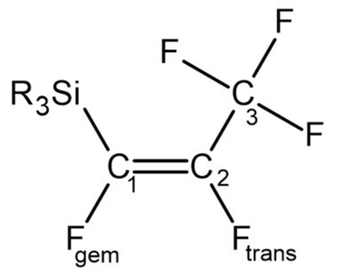

Figure 1 Skeleton of general structure of $R_{4-n}Si(E\text{-}CF{=}CFCF_3)_n$

1-1-1. Synthesis of $(Et)_3Si(E\text{-}CF{=}CFCF_3)$ (a.1)

Z-HFC-1225ye (4.7 mL, 48.0 mmol), ^nBuLi (2.5 M, 19.0 mL, 48.0 mmol), and $(Et)_3SiCl$ (5.0 mL, 32.0 mmol). The product was isolated as a yellow liquid after work up using (G.P1). Yield (4.0 g, 16.0 mmol, 50 %). Anal.Calcd for $C_9H_{15}F_5Si$: C, 43.89; H, 6.14 %. Found: C, 43.20; H, 5.95 %. $^{19}F\{^1H\}$ NMR (376 MHz, $CDCl_3$, 291 K) δ ppm: −68.13 (d.d, 3F, $^3J(CF_3\,F_{trans})$ = 13.8 Hz, $^4J(CF_3\,F_{gem})$ = 6.5 Hz, CF_3), −137.16 (q.d, 1F, $^3J(F_{gem}\,F_{trans})$ = 13.0 Hz, $^4J(F_{gem}\,CF_3)$ = 6.5 Hz, 1F, F_{gem}), −141.20 (q.d, $^3J(F_{trans}\,CF_3)$ = 13.8 Hz, $^3J(F_{trans}\,F_{gem})$ = 13.5 Hz, F_{trans}). ^1H NMR (400 MHz, $CDCl_3$, 291 K) δ ppm: 0.7 (q, 6H, $^3J(CH_2\,CH_3)$ = 7.9 Hz, CH_2), 0.92 (t, 9H, $^3J(CH_3\,CH_2)$ = 7.8 Hz, CH_3). $^{13}C\{^1H\}$ NMR (100 MHz, $CDCl_3$, 298 K) δ ppm: 0.05 (s, CH_2), 4.16 (s, CH_3), 117.00 (q.d.d, $^1J(C_3\,3F)$ = 270.6 Hz, $^2J(C_3\,F_{trans})$ = 37.0 Hz, $^3J(C_3\,F_{gem})$ = 10.3 Hz, C_3), 144.00 (d.q.d, $^1J(C_2\,F_{trans})$ = 270.4 Hz, $^2J(C_2\,3F)$ = 39.6 Hz, $^2J(C_2\,F_{gem})$ = 20.3 Hz, C_2), 155.20 (d.q.d, $^1J(C_1\,F_{gem})$ = 287.9 Hz, $^3J(C_1\,3F)$ = 4.7 Hz, $^2J(C_1\,F_{trans})$ = 1.7 Hz, C_1). $^{29}Si\{^1H\}$ NMR (79 MHz, $CDCl_3$, 298 K) δ ppm: 6.98 (d, $^1J(Si\,F_{gem})$ = 23.5 Hz). ν_{max}/ cm^{-1} 1240 (C-Si, w), 1360, 1410, 1460 (C-F, str), 1700 (C=C, m), 2870, 2910, 2955 (C-H, str).

1-1-2. Synthesis of $^nBu_3Si(E\text{-}CF{=}CFCF_3)$ (a.2)

Z-HFC-1225ye (4.7 mL, 45.0 mmol), ^nBuLi (2.5 M, 21.6 mL, 45.0 mmol), and $^nBu_3SiCl$ (8.0 mL, 30.0 mmol). The product was isolated as a yellow liquid after purification by column chromatography on silica with (1:1) diethyl ether : hexane eluent. Yield (5.0 g, 15.0 mmol, 50 %). Anal.Calcd for $C_{15}H_{27}F_5Si$: C, 54.52; H, 8.24 %. Found: C, 54.06; H, 8.20 %. $^{19}F\{^1H\}$ NMR (376 MHz, $CDCl_3$, 291 K) δ ppm: −67.91 (d.d, 3F, $^4J(CF_3\,F_{trans})$ = 13.7 Hz, $^4J(CF_3\,F_{gem})$ = 6.4 Hz, CF_3), −136.57 (q.d, 1F, $^3J(F_{gem}\,F_{trans})$ = 11.9 Hz, $^4J(F_{gem}\,CF_3)$ = 6.2 Hz, F_{gem}), −141.33 (d.q, 1F, $^3J(F_{trans}\,CF_3)$ = 13.8 Hz, $^3J(F_{trans}\,F_{gem})$ = 11.8 Hz, F_{trans}). ^1H NMR (400 MHz, $CDCl_3$, 291 K) δ ppm: 0.43 (t, 6H, $^3J(CH_2\,CH_3)$ = 6.0 Hz, CH_2), 0.82 (t, 9H, $^3J(CH_3\,CH_2)$ = 7.0 Hz, CH_3), 1.23 (m, 12H, CH_2CH_2). $^{13}C\{^1H\}$ NMR (100 MHz, $CDCl_3$, 298 K) δ ppm: 13.78 (s, Si-CH_2), 15.46 (s, CH_3), 25.52 (s, CH_2), 26.66 (s, CH_2), 119.40 (m, C_3), 145.80 (m, C_2), 158.40 (m, C_1). $^{29}Si\{^1H\}$ NMR (79 MHz, $CDCl_3$, 298 K) δ ppm: 3.00 (d, $^1J(Si\,F_{gem})$ = 23.6 Hz). ν_{max}/cm^{-1} 1295 (C-Si, m), 1365, 1460, 1475(C-F, str), 2870, 2920, 2950 (C-H, str).

1-1-3. Synthesis of $ClCH_2(Me)_2Si(E\text{-}CF{=}CFCF_3)$ (a.3)

Z-HFC-1225ye (3.0 mL, 30.0 mmol), ^nBuLi (2.5 M, 12.0 mL, 30 mmol), and $ClCH_2(CH_3)_2SiCl$ (2.6 mL, 20.0 mmol). The product was isolated as a yellow liquid after work up using (G.P1). Yield (3.0 g, 12.6 mmol, 63 %). Anal.Calcd for $C_6H_8ClF_5Si$: C, 30.19; H, 3.38; Cl, 14.87 %. Found: C, 30.06; H, 3.15; Cl, 14.18 %. $^{19}F\{^1H\}$ NMR (376 MHz, $CDCl_3$, 291K) δ ppm: −67.83 (d.d, 3F, $^3J(CF_3\,F_{trans})$ = 13.3 Hz, $^4J(CF_3\,F_{gem})$ = 6.2 Hz, CF_3), −139.14 (m, 1F, F_{trans}), −139.02 (m, 1F, F_{gem}). ^1H NMR (400 MHz, $CDCl_3$, 291K) δ ppm: 0.21(s, 3H, CH_3), 2.75 (s, 2H, CH_2). $^{13}C\{^1H\}$ NMR (100 MHz, $CDCl_3$, 298 K) δ ppm: −5.41 (s, CH_3), 27.32 (s, CH_2), 119.00 (q.d.d, $^1J(C_3\,3F)$ = 270.3 Hz, $^2J(C_3\,F_{trans})$ = 36.6 Hz, $^3J(C_3\,F_{gem})$ = 10.2 Hz, C_3), 146.40 (d.d.q,$^1J(C_2\,F_{trans})$ = 253.8 Hz, $^2J(C_2\,3F)$ = 39.7 Hz, $^2J(C_2\,F_{gem})$ = 20.8 Hz, C_2), 155.95 (d.q,$^1J(C_1\,F_{gem})$ = 287.2 Hz, $^3J(C_1\,3F)$ = 4.6 Hz, C_1). $^{29}Si\{^1H\}$ NMR (79 MHz, $CDCl_3$, 298 K) δ ppm: −1.50 (d, $^1J(Si\,F_{gem})$ = 27.5 Hz). ν_{max}/cm^{-1} 1260 (C-Si, m), 1360 (C-F, str), 1665 (C=C, w), 2965 (C-H, str).

1-1-4. Synthesis of $^nBu(Me)_2Si(E\text{-}CF{=}CFCF_3)$ (a.4)

Z-HFC-1225ye (1.8 mL, 18.0 mmol), ^nBuLi (2.5 M, 7.2 mL, 18.0 mmol), and $^nBu(Me)_2SiCl$ (2.0 mL, 12.0 mmol). The product was isolated as a yellow liquid after work up using (G.P1). Yield (2.0 g, 8.0 mmol, 66 %). Anal.Calcd for $C_9H_{15}F_5Si$: C, 43.89; H, 6.14 %. Found: C, 43.55; H, 6.02 %. $^{19}F\{^1H\}$ NMR (376 MHz, $CDCl_3$, 291K) δ ppm: −67.62 (d.d, 3F, $^3J(CF_3\,F_{trans})$ = 13.7 Hz, $^4J(CF_3\,F_{gem})$ = 6.5 Hz, CF_3), −137.26 (q.d, 1F, $^4J(F_{gem}\,3F)$ = 6.8 Hz, $^3J(F_{gem}\,F_{trans})$ = 12.2 Hz, F_{gem}), −141.89 (q.d, 1F, $^3J(F_{trans}\,3F)$ = 13.2 Hz, $^3J(F_{trans}\,F_{gem})$ = 12.8 Hz, F_{trans}). ^1H NMR (400 MHz, $CDCl_3$, 291 K) δ ppm: 0.23 (s, 6H, CH_3-Si), 0.72 (t, 2H, $^3J(CH_2\,CH_3)$ = 6.5 Hz, CH_2), 0.86 (q, 3H, $^3J(CH_3\,CH_2)$ = 6.8 Hz, CH_3), 1.31 (m, 4H, CH_2CH_2). $^{13}C\{^1H\}$ NMR (100 MHz, $CDCl_3$, 298 K) δ ppm: −4.24 (s, CH_3-Si), 13.27 (s, CH_2), 13.66 (s, CH_3), 25.37 (s, CH_2), 26.10 (s, CH_2), 119.40 (q.d.d, $^1J(C_3\,3F)$ = 270.0 Hz, $^2J(C_3\,F_{trans})$ = 36.8 Hz, $^3J(C_3\,F_{gem})$ = 10.4 Hz, C_3), 145.60 (d.q.d, $^1J(C_2\,F_{trans})$ = 270.8 Hz, $^2J(C_2\,3F)$ = 39.8 Hz, $^2J(C_2\,F_{gem})$ = 20.0 Hz, C_2), 158.50 (d.q.d, $^1J(C_1\,F_{gem})$ = 287.5 Hz, $^3J(C_1\,3F)$ = 4.8 Hz, $^2J(C_1\,F_{trans})$ = 1.8 Hz, C_1). $^{29}Si\{^1H\}$ NMR (79 MHz, $CDCl_3$, 298 K) δ ppm: 1.05 (d, $^1J(Si\,F_{gem})$ = 26.4 Hz). ν_{max}/cm^{-1} 1295 (C-Si, m), 1380, 1465 (C-F, str), 1660 (C=C, w), 2960, 2925, 2870 (C-H, str).

1-1-5. Synthesis of $(Me)_2PhSi(E\text{-}CF{=}CFCF_3)$ (a.5)

Z-HFC-1225ye (1.6 mL, 16.0 mmol), ^nBuLi (2.5 M, 6.4 mL, 16.0 mmol), and $(Me)_2PhSiCl$ (1.6 mL, 10.0 mmol). The product was isolated as a yellow liquid after work up using (G.P1). Yield (1.5 g, 5.6 mmol, 56 %). Anal.Calcd for $C_{11}H_{11}F_5Si$: C, 49.61; H, 4.17 %. Found: C, 49.31; H, 3.90 %. $^{19}F\{^1H\}$ NMR (376 MHz, $CDCl_3$) δ ppm: −67.36 (d.d, 3F, $^4J(CF_3\ F_{trans})$ = 13.2 Hz, $^3J(CF_3\ F_{gem})$ = 6.3 Hz, CF_3), −136.30 (q.d, 1F, $^3J(F_{gem}\ F_{trans})$ = 12.9 Hz, $^3J\ (F_{trans}\ CF_3)$ = 6.2 Hz, F_{gem}), −140.89 (q.d, 1F, $^3J(F_{trans}\ CF_3)$ = 13.2 Hz, $^3J(F_{trans}\ F_{gem})$ = 12.9 Hz, F_{trans}). ^1H NMR (400 MHz, $CDCl_3$, 291 K) δ ppm: 0.46 (s, 6H, CH_3-Si), 7.30-7.43 (m, 5H, Ph). $^{13}C\{^1H\}$ NMR (100 MHz, $CDCl_3$, 298 K) δ ppm: −4.39 (s, CH_3-Si), 118.70 (q.d.d, $^1J(C_3\ 3F)$ = 270.0 Hz, $^2J(C_3\ F_{trans})$ = 36.5 Hz, $^3J(C_3\ F_{gem})$ = 10.4 Hz, C_3), 127.52 (s, Ph), 129.63 (s, Ph), 132.78 (s, Ph), 132.97 (s, Ph), 145.45 (d.q.d, $^1J(C_2\ F_{trans})$ = 271.6 Hz, $^2J(C_2\ 3F)$ = 40.7 Hz, $^2J(C_2\ F_{gem})$ = 19.9 Hz, C_2), 157.80 (d.q, $^1J(C_1\ F_{gem})$ = 286.9 Hz, $^3J(C_1\ 3F)$ = 4.5 Hz, C_1). $^{29}Si\{^1H\}$ NMR (79 MHz, $CDCl_3$, 298 K) δ ppm: −6.40 (d, $^1J(Si\ F_{gem})$ = 28.7 Hz). ν_{max}/cm^{-1} 1260 (C-Si, m), 1360, 1430 (C-F, str), 1660 (C=C, w), 3070 (C-H, m).

1-1-6. Synthesis of $(Ph)_2MeSi(E\text{-}CF{=}CFCF_3)$ (a.6)

Z-HFC-1225ye (1.5 mL, 14.0 mmol), ^nBuLi (2.5 M, 5.6 mL, 14.0 mmol), and $(Ph)_2MeSiCl$ (1.8 mL, 9.0 mmol). The product was isolated as a yellow liquid after work up using (G.P1). Yield (1.5 g, 4.5 mmol, 50 %). Anal.Calcd for $C_{16}H_{13}F_5Si$: C, 58.52; H, 3.99 %. Found: C, 58.59; H, 4.07 %. $^{19}F\{^1H\}$ NMR (376 MHz, $CDCl_3$, 291 K) δ ppm: −67.06 (d.d, 3F, $^3J(CF_3\ F_{trans})$ = 13.7 Hz, $^4J(CF_3\ F_{gem})$ = 6.2 Hz, CF_3), −134.12 (q.d, 1F, $^3J(F_{gem}\ F_{trans})$ = 12.2 Hz, $^4J(F_{gem}\ CF_3)$ = 6.1 Hz, F_{gem}), −138.22 (q.d, 1F, $^3J(F_{trans}\ CF_3)$ = 13.0 Hz, $^3J(F_{trans}\ F_{gem})$ = 12.5 Hz, F_{trans}). ^1H NMR (400 MHz, $CDCl_3$, 291 K) δ ppm: 1.18 (s, 3H, CH_3-Si), 7.34-7.48 (m, 10H, Ph). $^{13}C\{^1H\}$ NMR (100 MHz, $CDCl_3$, 298 K) δ ppm: −3.82 (s, CH_3-Si), 119.00 (q.d.d, $^1J(C_3\ 3F)$ = 270.4 Hz, $^2J(C_3\ F_{trans})$ = 36.6 Hz, $^3J(C_3\ F_{gem})$ = 9.9 Hz, C_3), 128.27 (s, Ph), 130.58 (s, Ph), 131.33 (s, Ph), 134.83 (s, Ph), 147.00 (d.q.d, $^1J(C_2\ F_{trans})$ = 273.7 Hz, $^2J(C_2\ 3F)$ = 40.5 Hz, $^2J(C_2\ F_{gem})$ = 20.7 Hz, C_2), 155.80 (d.q, $^1J(C_1\ F_{gem})$ = 286.1 Hz, $^3J(C_1\ 3F)$ = 4.4 Hz, C_1). $^{29}Si\{^1H\}$ NMR (79 MHz, $CDCl_3$, 298 K) δ ppm: −12.28 (d, $^1J(Si\ F_{gem})$ = 28.8 Hz). ν_{max}/cm^{-1} 1260 (C-Si, m), 1350, 1430 (C-F, str), 1600 (C=C, w), 3070 (C-H, m).

1-1-7. Synthesis of $(Me)_2Si(E\text{-}CF{=}CFCF_3)_2$ (a.7)

Z-HFC- 225ye (4.7 mL, 12.0 mmol), ^nBuLi (2.5 M, 4.8 mL, 12.0 mmol), and $(Me)_2SiCl_2$ (0.5 mL, 4.0 mmol). The product was isolated as a yellow liquid after purification by column chromatography on silica with (1:1) diethyl ether : hexane eluent. Yield (0.9 g, 2.8 mmol, 70 %). $^{19}F\{^1H\}$ NMR (376 MHz, $CDCl_3$, 291 K) δ ppm: −68.83 (d.d, 6F, $^3J(CF_3\ F_{trans})$ = 13.2 Hz, $^4J(CF_3\ F_{gem})$ = 5.4 Hz, CF_3), −137.75 (q.d, 2F, $^4J(F_{gem}\ CF_3)$ = 5.8 Hz, $^3J(F_{gem}\ F_{trans})$ = 12.7 Hz, F_{gem}), −140.36 (m, 2F, F_{trans}). ^1H NMR (400 MHz, $CDCl_3$, 291 K) δ ppm: 0.63 (s, 6H, CH_3). $^{13}C\{^1H\}$ NMR (100 MHz, $CDCl_3$, 298 K) δ ppm: 10.75 (s, CH_3), 118.00 (q.d.d, $^1J(C_3\ 3F)$ = 271.1 Hz, $^2J(C_3\ F_{trans})$ = 37.2 Hz, $^3J(C_3\ F_{gem})$ = 9.8 Hz, C_3), 145.00 (m, C_2), 152.00 (d.m, $^1J(C_1\ F_{gem})$ = 286.1 Hz, C_1). $^{29}Si\{^1H\}$ NMR (79 MHz, $CDCl_3$, 298 K) δ ppm: −21.00 (m). ν_{max}/cm^{-1} 1260 (C-Si, m), 1360 (C-F, str), 1670 (C=C, w), 2960 (C-H, str).

1-1-8. Synthesis of $(^iPr)_2Si(E\text{-}CF{=}CFCF_3)_2$ (a.8)

Z-HFC-1225ye (6.6 mL, 50.0 mmol), ^nBuLi (2.5 M, 20.0 mL, 50.0 mmol), and $(^iPr)_2SiCl_2$ (2.8 mL, 16.0 mmol). The product was isolated as a yellow liquid after purification by column chromatography on silica with (1:1) diethyl ether : hexane eluent. Yield (2.0 g, 5.0 mmol, 31 %). $^{19}F\{^1H\}$ NMR (376 MHz, $CDCl_3$, 291 K) δ ppm: −68.97 (d.d, 6F, $^3J(CF_3\ F_{trans})$ = 14.1 Hz, $^4J(CF_3\ F_{gem})$ = 5.8 Hz, CF_3), −136.95 (q.d, 2F, $^4J(F_{gem}\ CF_3)$ = 4.8 Hz, $^3J(F_{gem}\ F_{trans})$ = 12.7 Hz, F_{gem}), −138.54 (m, 2F, F_{trans}). ^1H NMR (400 MHz, $CDCl_3$, 291 K) δ ppm: 1.07 (d, 12H, $^3J(CH_3\ CH)$ = 7.5 Hz, CH_3), 1.45 (septet, 2H, $^3J(CH\ CH_3)$ = 7.4 Hz, CH). $^{13}C\{^1H\}$ NMR (100 MHz, $CDCl_3$, 298 K) δ ppm: 10.75 (s, CH-Si), 15.43 (s, CH_3), 118.70 (q.d.d, $^1J(C_3\ 3F)$ = 270.5 Hz, $^2J(C_3\ F_{trans})$ = 36.8 Hz, $^3J(C_3\ F_{gem})$ = 9.7 Hz, C_3), 147.55 (d.q.d, $^1J(C_2\ F_{trans})$ = 274.4 Hz, $^2J(C_2\ 3F)$ = 41.0 Hz, $^2J(C_2\ F_{gem})$ = 19.2 Hz, C_2), 152.70 (m, overlap, C_1). $^{29}Si\{^1H\}$ NMR (79 MHz, $CDCl_3$, 298 K) δ ppm: −1.57 (m). ν_{max}/cm^{-1} 2870, 2950 (C-H, str), 1650 (C=C, w), 1250 (C-Si, m), 1360, 1390, 1460 (C-F, str).

1-1-9. Synthesis of $(Ph)_2Si(E\text{-}CF{=}CFCF_3)_2$ (a.9)

Z-HFC-1225ye (4.5 mL, 45.0 mmol), ^nBuLi (2.5 M, 18.0 mL, 45.0 mmol), Ph_2SiCl_2 (3.0 mL, 15.0 mmol). The product was isolated as a light brown solid after work up and purification by column chromatography on silica with (1:1) diethyl ether: hexane eluent. Yield (1.9 g, 4.3 mmol, 28 %) M.P (98-100 °C). Anal.Calcd for $C_{18}H_{10}F_{10}Si$: C, 48.64; H, 2.27 %.

Found: C, 48.23; H, 2.24 %. $^{19}F\{^1H\}$ NMR (376 MHz, $CDCl_3$, 291 K) δ ppm: −67.95 (d.d.d, 6F, $^3J(CF_3\ F_{trans})$ = 13.0 Hz, $^4J(CF_3\ F_{gem})$ = 4.5 Hz, $J(CF_3\ F_{external})$ = 3.9 Hz, CF_3), −133.95 (q.d, 2F, $^4J(F_{gem}\ CF_3)$ = 5.5 Hz, $^3J(F_{gem}\ F_{trans})$ = 13.1 Hz, F_{gem}), −136.94 (m, 2F, F_{trans}). ^1H NMR (400 MHz, $CDCl_3$, 291 K) δ ppm: 7.39-7.41 (m, 10H, Ph). $^{13}C\{^1H\}$ NMR (100 MHz, $CDCl_3$, 298 K) δ ppm: 118.50 (q.d.d, $^1J(C_3\ 3F)$ = 271.6 Hz, $^2J(C_3\ F_{trans})$ = 36.2 Hz, $^3J(C_3\ F_{gem})$ = 9.4 Hz, C_3), 125.50 (s, Ph), 128.58 (s, Ph), 131.78 (s, Ph), 135.49 (s, Ph), 147.80 (d.q.d, $^1J(C_2\ F_{trans})$ = 277.8 Hz, $^3J(C_2\ 3F)$ = 40.7 Hz, $^2J(C_2\ F_{gem})$ = 20.1 Hz, C_2), 150.80 (d, $^1J(C_1\ F_{gem})$ = 284.1 Hz, C_1). $^{29}Si\{^1H\}$ NMR (79 MHz, $CDCl_3$, 298 K) δ ppm: −23.20 (m). ν_{max}/ cm^{-1} 3070 (C-H, str), 1665 (C=C, w), 1490, 1430 (C-F, str), 1200 (C-Si, m).

1-1-10. Synthesis of PhSi(*E*-CF=CFCF$_3$)$_3$ (a.10)

Z-HFC-1225ye (2.7 mL, 27.0 mmol), ^nBuLi (2.5 M, 10.8 mL, 27.0 mmol), and $PhSiCl_3$ (0.9 mL, 6.0 mmol). The product was isolated as a brown liquid after work up using (G.P1). Yield (2.0 g, 4.0 mmol, 66 %). Anal.Calcd for $C_{15}H_5F_{15}Si$: C, 36.17; H, 1.01 %. Found: C, 36.14; H, 1.01 %. $^{19}F\{^1H\}$ NMR (376 MHz, $CDCl_3$, 291 K) δ ppm: −69.31 (broad.d, 9F, $^3J(CF_3\ F_{trans})$ = 13.4 Hz, CF_3), −131.04 (d.q, 3F, $^4J(F_{gem}\ CF_3)$ = 5.9 Hz, $^3J(F_{gem}\ F_{trans})$ = 13.5 Hz, F_{gem}), −141.08 (m, 3F, F_{trans}). ^1H NMR (400 MHz, $CDCl_3$, 291 K) δ ppm: 7.40-7.51 (m, 5H, Ph,). $^{13}C\{^1H\}$ NMR (100 MHz, $CDCl_3$, 298 K) δ ppm: 118.50 (q.d.d, $^1J(C_3\ 3F)$ = 271.2 Hz, $^2J(C_3\ F_{trans})$ = 36.6 Hz, $^3J(C_3\ F_{gem})$ = 9.0 Hz, C_3), 120.95 (s, Ph), 128.74 (s, Ph), 132.73 (s, Ph), 134.84 (s, Ph), 148.50 (d.q.d, $^1J(C_2\ F_{trans})$ = 281.4 Hz, $^2J(C_2\ 3F)$ = 40.9 Hz, $^2J(C_2\ F_{gem})$ = 19.6 Hz, C_2), 147.00 (d, $^1J(C_1\ F_{gem})$ = 283.0 Hz, C_1). $^{29}Si\{^1H\}$ NMR (79 MHz, $CDCl_3$, 298 K) δ ppm: −28.00 (m). ν_{max}/cm^{-1} 1380, 1430 (C-F, str), 1245 (C-Si, m), 1670 (C=C, w).

1-1-11. Synthesis of Si(*E*-CF=CFCF$_3$)$_4$ (a.11)

Z-HFC-1225ye (6.0 mL, 57.0 mmol), ^nBuLi (2.5 M, 22.8 mL, 57.0 mmol), and $SiCl_4$ (1.0 mL, 9.5 mmol), the product was analysed in solution because it could not be isolated from the solvent. $^{19}F\{^1H\}$ NMR (376 MHz, $CDCl_3$, 291 K) δ ppm: −70.05 (d.m, 12F, $^3J\ (CF_3\ F_{trans})$ = 16.1 Hz, CF_3), −127.32 (q.d, 4F, $^4J(F_{gem}\ CF_3)$ = 6.5 Hz, $^3J(F_{gem}\ F_{trans})$ = 13.0 Hz, F_{gem}), −145.45 (m, 4F, F_{trans}). $^{13}C\{^1H\}$ NMR (100 MHz, $CDCl_3$, 298 K) δ ppm: 118.50 (q, $^1J(C_3\ 3F)$ = 270.0 Hz, C_3), 148.20 (m, C_2), 137.80 (d.q, $^1J(C_1\ F_{gem})$ = 263.2 Hz, $^3J(C_1\ 3F)$ = 5.6 Hz, C_1). $^{29}Si\{^1H\}$ NMR (79 MHz, $CDCl_3$, 298 K) δ ppm: −67.37 (m). ν_{max}/cm^{-1} 1295 (C-Si, m), 1360, 1465 (C-F, str), 1670 (C=C, w).

2. R$_{4-n}$Si(*E*-CF=CFCF$_3$)$_n$ Reactions

2-1. General Substitution Reactions (G.P2)

A solution of dry THF (150 mL) and $R_{4-n}Si(CF_3CF=CF)_n$ was placed in a three-necked round-bottom flask under a positive pressure of nitrogen. The solution was cooled to −30 °C, and then RLi (solution in hexanes) was added slowly. The mixture was slowly warmed to room temperature and left stirring for 24 hours. The reaction was worked up with hexanes (10 mL), followed by filtration through celite, and solvent was removed using a rotary evaporator.

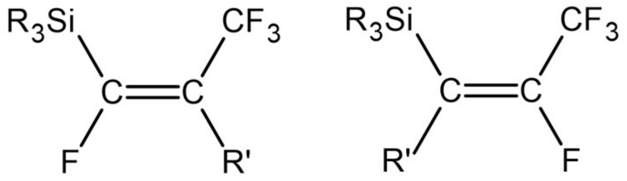

R$_3$Si(*Z*-CF=CR'CF$_3$) R$_3$Si(*E*-CR'=CFCF$_3$)

Figure 2 Skeleton of general structure of *E* and *Z* isomers ((R= Me, Et, ^nBu, ^iPr, Ph, and R`= ^nBu, ^tBu, Me, Ph)

2-1-1. Reaction between Et$_3$Si(*E*-CF=CFCF$_3$) (a.1) and ^{n}BuLi

Et_3Si(*E*-CF=CFCF$_3$) (a.1) (1.0 g, 4.0 mmol) was dissolved in dry THF (150 mL), ^nBuLi (2.5 M, 3.2 mL, 8.0 mmol) was added slowly. The product was isolated as a black liquid after work up using (G.P2). Yield (0.8 g, 2.8 mmol, 70 %); ratio *Z*:*E* [Et_3Si(*Z*-CF=C^{n}BuCF$_3$) (12*Z*) 88 : Et_3Si(*E*-C^{n}Bu=CFCF$_3$) (12*E*) 12]. Anal.Calcd for $C_{13}H_{24}F_4Si$: C, 54.90; H, 8.51 %. Found: C, 54.55; H, 8.23 %. $^{19}F\{^1H\}$ NMR (376 MHz, $CDCl_3$, 291 K) δ ppm: Et_3Si(*Z*-CF=C^{n}BuCF$_3$) −60.28 (d, 3F, $^4J(CF_3\ F_{gem})$ = 8.6 Hz, CF_3), −99.31 (q, 1F, $^4J(F\ CF_3)$ = 8.2 Hz, F_{gem}) and Et_3Si(*E*-C^{n}Bu=CFCF$_3$) −67.34 (d, 3F, $^3J(CF_3\ F_{trans})$ = 10.6 Hz,

CF_3), −106.75 (q, 1F, ^3J(F_{trans} CF_3) = 10.9 Hz, F_{trans}). ^{1}H NMR (400 MHz, $CDCl_3$, 291 K) δ ppm: Et_3Si(*Z*-CF=C^{n}BuCF_3) and Et_3Si(*E*-C^{n}Bu=CFCF_3) 0.45 (q, 6H, ^3J(CH_2 CH_3) = 8.2 Hz, Si-CH_2), 0.49 (q, 6H, ^3J(CH_2 CH_3) = 7.9 Hz, Si-CH_2), 0.68 (t, 9H, ^3J(CH_3 CH_2) = 7.8 Hz, CH_3), 0.77 (t, 9H, ^3J(CH_3 CH_2) = 7.4 Hz, CH_3), 0.80 (t, 3H, ^3J(CH_3 CH_2) = 7.8 Hz, CH_3), 0.81 (t, 3H, ^3J(CH_3 CH_2) = 7.6 Hz, CH_3), 1.25-1.43 (m, 12H, -CH_2CH_2-), 2.14 (t, 2H, ^3J(CH_2 CH_2) = 7.3 Hz, CH_2-C=), 2.86 (t, 2H, ^3J(CH_2 CH_2) = 7.9 Hz, CH_2-C=). $^{13}C\{^1H\}$ NMR (100 MHz, $CDCl_3$, 298 K) δ ppm: Et_3Si(*Z*-CF=C^{n}BuCF_3) and Et_3Si(*E*-C^{n}Bu=CFCF_3): 1.82 (s, Si-CH_2), 2.61 (s, Si-CH_2), 6.05 (s, CH_3), 6.31 (s, CH_3), 10.10 (s, =C-CH_2), 12.68 (s, =C-CH_2), 21.86 (s, CH_3), 21.68 (s, CH_3), 25.30 (s, -CH_2CH_2-), 25.93 (s, CH_2CH_2), 30.00 (s, -CH_2CH_2-), Et_3Si(*Z*-CF=C^{n}BuCF_3) 124.50 (q.d, ^1J(C_3 3F) = 272.7 Hz, ^3J(C_3 F_{gem}) = 24.3 Hz, C_3), 127.30 (q.d, ^3J(C_2 3F) = 28.7 Hz, ^2J(C_2 F_{gem}) = 18.6 Hz, C_2), 171.60 (d.q, ^1J(C_1 F_{gem}) = 288.0 Hz, ^3J(C_1 3F) = 6.6 Hz); Et_3Si(*E*-C^{n}Bu=CFCF_3), 118.00 (q.d, ^1J(C_3 3F) = 272.7 Hz, ^2J(C_3 F_{trans}) = 50.7 Hz), 119.75 (d, ^2J(C_1 F_{trans}) = 8.7 Hz, C_1), 147.00 (d.q, ^1J(C_2 F_{trans}) = 267.6 Hz, ^2J(C_2 3F) = 39.5 Hz, C_2). $^{29}Si\{^1H\}$ NMR (79 MHz, $CDCl_3$, 298 K) δ ppm: Et_3Si(*Z*-CF=C^{n}BuCF_3) 6.20 (d, ^2J(Si F_{gem}) = 31.2 Hz); Et_3Si(*E*-C^{n}Bu=CFCF_3), 4.96 (d, ^3J(Si F_{trans}) = 9.4 Hz). ν_{max}/ cm^{-1} 1300 (CF_3, srt), 1460, 1360 (C-F, m), 2850, 2920, 2960(C-H, w).

2-1-2. Reaction between Et_3Si(*E*-CF=CFCF_3) (a.1) and ^{t}BuLi

Et_3Si(*E*-CF=CFCF_3) (a.1) (1.0 g, 4.0 mmol) was dissolved in dry THF (150 mL), ^{t}BuLi (1.7 M, 7.7 mL, 8.0 mmol) was added slowly. The product was isolated as a black liquid after work up using (G.P2). Yield (0.5 g, 1.7 mmol, 42 %). Anal.Calcd for $C_{13}H_{24}F_4Si$: C, 54.90; H, 8.51 %. Found: C, 54.89; H, 8.03 %. $^{19}F\{^1H\}$ NMR (376 MHz, $CDCl_3$, 291 K) δ ppm −55.85 (d, 3F, ^4J(CF_3 F_{gem}) = 7.4 Hz, CF_3), −87.06 (q, 1F, ^4J(F_{gem} CF_3) = 7.1 Hz, F_{gem}). ^{1}H NMR (400 MHz, $CDCl_3$, 291 K) δ ppm: 0.66 (q, 6H, ^3J(CH_3 CH_2) = 7.1 Hz, Si-CH_2), 0.89 (t, 9H, ^3J(CH_3 CH_2) = 7.7 Hz, CH_3), 1.21 (s, 9H, $(CH_3)_3$-C). $^{13}C\{^1H\}$ NMR (100 MHz, $CDCl_3$, 298 K) δ ppm: 2.29 (s, Si-CH_2), 6.23 (s, CH_3), 28.90 (s, =C-$\underline{C}$-$(CH_3)_3$) 28.94 (s, C-($\underline{C}H_3)_3$), 124.50 (q.d, ^1J(C_3 3F) = 273.0 Hz, ^3J(C_3 F_{gem}) = 27.9 Hz, C_3), 132.92 (q.d, ^2J(C_2 3F) = 24.8 Hz, ^2J(C_2 F_{gem}) = 12.8 Hz), 173.60 (d.q, ^1J(C_1 F_{gem}) = 293.0 Hz, ^3J(C_1 3F) = 9.5 Hz). $^{29}Si\{^1H\}$ NMR (79 MHz, $CDCl_3$, 298 K) δ ppm: 8.70 (d, ^2J(Si F_{gem}) = 32.8 Hz). ν_{max}/ cm^{-1} 1230 (C-Si, m), 1320, 1365 (C-F, str), 1600 (C=C, w), 2870, 2920 (C-H, w).

2-1-3. Reaction between Et_3Si(*E*-CF=CFCF_3) (a.1) and MeLi

Et_3Si(*E*-CF=CFCF_3) (a.1) (1.0 g, 4.0 mmol) was dissolved in dry diethyl ether (150 mL), MeLi (1.6 M, 12.5 mL, 20.0 mmol) was added slowly. The product was isolated as a black liquid after work up using (G.P2). Yield (0.7 g, 2.8 mmol, 70 %); ratio *Z*:*E* [Et_3Si(*Z*-CF=CMeCF_3) (14*Z*) 79 : Et_3Si(*E*-CMe=CFCF_3) (14*E*) 21]. Anal.Calcd for $C_{10}H_{18}F_4Si$: C, 49.56; H, 7.49 %. Found: C, 49.73; H, 7.12 %. $^{19}F\{^1H\}$ NMR (376 MHz, $CDCl_3$, 291 K) δ ppm: Et_3Si(*Z*-CF=CMeCF_3) −62.50 (d, 3F, ^4J(CF_3 F_{gem}) = 8.6 Hz, CF_3), −98.47 (q, 1F, ^4J(F_{gem} CF_3) = 8.7 Hz, F_{gem}), and Et_3Si(*E*-CMe=CFCF_3) −67.07 (d, 3F, ^3J(CF_3 F_{trans}) = 10.3 Hz, CF_3), −105.47 (q, 1F, ^3J(F_{trans} CF_3) = 10.3 Hz, F_{trans}). ^{1}H NMR (400 MHz, $CDCl_3$, 291 K) δ ppm: Et_3Si(*Z*-CF=CMeCF_3), and Et_3Si(*E*-CMe=CFCF_3): 0.68 (q, 6H, ^3J(CH_2 CH_3) = 7.9 Hz, Si-CH_2), 0.70 (q, 6H, ^3J(CH_2 CH_3) = 7.3 Hz, Si-CH_2), 0.81 (t, 9H, ^3J(CH_3 CH_2) = 7.8 Hz, CH_3), 0.88 (t, 9H, ^3J(CH_3 CH_2) = 7.6 Hz, CH_3), 1.77 (s, 3H, =C-CH_3), 1.79 (s, 3H, =C-CH_3). $^{13}C\{^1H\}$ NMR (100 MHz, $CDCl_3$, 298 K) δ ppm: Et_3Si(*Z*-CF=CMeCF_3), and Et_3Si(*E*-CMe=CFCF_3) 1.75 (s, Si-CH_2), 2.35 (s, Si-CH_2), 5.35 (s, CH_3), 5.78 (s, CH_3), 6.1 (s, =C($\underline{C}H_3)_3$), 6.22 (s, =C($\underline{C}H_3)_3$); Et_3Si(*Z*-CF=CMeCF_3) 125.00 (q.d, ^1J(C_3 3F) = 275.7 Hz, ^4J(C_3 F_{gem}) = 25.2 Hz, C_3), 122.30 (q.d, ^2J(C_2 3F) = 30.4 Hz, ^2J(C_2 F_{gem}) = 19.1 Hz, C_2), 171.00 (d.q, ^1J(C_1 F_{gem}) = 287.9 Hz, ^3J(C_1 3F) = 6.6 Hz, C_1), Et_3Si(*E*-CMe=CFCF_3): 126.50 (m, C_3), 122.46 (d, ^2J(C_1 F_{trans}) = 4.3 Hz, C_1), 119.90 (m, C_2). $^{29}Si\{^1H\}$ NMR (79 MHz, $CDCl_3$, 298 K) δ ppm: Et_3Si(*Z*-CF=CMeCF_3) 6.15 (d, ^2J(Si F_{gem}) = 30.7 Hz); Et_3Si(*E*-CMe=CFCF_3) 8.95 (d, ^2J(Si F_{trans}) = 6.8 Hz). ν_{max}/cm^{-1} 1260 (Si-C, m), 1335, 1380 (C-F,str), 1680 (C=C,w), 2960 (C-H,w).

2-1-4. Reaction between Et_3Si(*E*-CF=CFCF_3) (a.1) and PhLi

(Et)$_3$Si(*E*-CF=CFCF_3) (a.1) (1.0 g, 4.0 mmol) was dissolved in dry diethyl ether (150 mL), PhLi (2.0 M, 10.0 mL, 20.0 mmol) was added slowly. The product was isolated as a yellow liquid after purification by column chromatography on silica with a hexane eluent. Yield (0.9 g, 3.0 mmol, 75 %); ratio *Z*:*E* [Et_3Si(*Z*-CF=CPhCF_3) (15*Z*) 77 : Et_3Si(*E*-CPh=CFCF_3) (15*E*) 23]. Anal.Calcd for $C_{15}H_{20}F_4Si$: C, 59.19; H, 6.63 %. Found: C, 59.63; H, 6.91 %. $^{19}F\{^1H\}$NMR (376 MHz, $CDCl_3$, 291 K) δ ppm: Et_3Si(*Z*-CF=CPhCF_3) −58.61 (d, 3F, ^4J(CF_3 F_{gem}) = 9.0 Hz, CF_3), −93.33 (q, 1F, ^4J(F_{gem} CF_3) = 9.2 Hz, F_{gem}), and Et_3Si(*E*-CPh=CFCF_3) −67.77 (d, 3F, ^3J(CF_3 F_{trans}) = 10.6 Hz, CF_3), −100.27 (q, 1F, ^4J(F_{trans} CF_3) = 10.5 Hz, F_{trans}). ^{1}H NMR (400 MHz, $CDCl_3$, 291 K) δ ppm: Et_3Si(*Z*-CF=CPhCF_3), and Et_3Si(*E*-CPh=CFCF_3): 0.52 (q, 6H, ^3J(CH_2 CH_3) = 7.1 Hz, Si-CH_2), 0.77 (q, 6H, ^3J(CH_2 CH_3) = 7.8 Hz, Si-CH_2), 0.82 (t, 9H, ^3J(CH_3 CH_2) = 7.6 Hz, CH_3), 0.96 (t, 9H,

$^{3}J(CH_3\ CH_2)$ = 7.8 Hz, CH_3), 7.24-7.63 (m, 10H, Ph). $^{13}C\{^{1}H\}$ NMR (100 MHz, $CDCl_3$, 298 K) δ ppm: Et_3Si(*Z*-CF=CPhCF$_3$), and Et_3Si(*E*-CPh=CFCF$_3$): 2.88 (s, CH_3), 3.98 (s, CH_3), 7.23 (s, Si-CH_2), 7.45 (s, Si-CH_2), 126.68 (s, Ph), 127.13 (s, Ph), 127.69 (s, Ph),128.74 (s, Ph), 128.34 (s, Ph), 128.63 (s, Ph), 134.23 (s, Ph), 129.45 (s, Ph), Et_3Si(*Z*-CF=CPhCF$_3$): 124.00 (q.d, $^{1}J(C_3\ 3F)$ = 270.9 Hz, $^{3}J(C_3\ F_{gem})$ = 20.3 Hz), 136.20 (d, $^{2}J(C_2\ F_{gem})$ = 10.3 Hz), 124.00 (d.q, $^{1}J(C_1\ F_{gem})$ = 270.9 Hz, $^{3}J(C_1\ 3F)$ = 20.3 Hz), Et_3Si(*E*-CPh=CFCF$_3$) 123.90 (m, C_3), 147.50 (m, C_2), 129.94 (m, C_1). $^{29}Si\{^{1}H\}$ NMR (79 MHz, $CDCl_3$, 298 K) δ ppm: Et_3Si(*Z*-CF=CPhCF$_3$) 7.90 (d, $^{2}J(Si\ F_{gem})$ = 30.3 Hz); Et_3Si(*E*-CPh=CFCF$_3$) 5.73 (d, $^{3}J(Si\ F_{trans})$ = 5.7 Hz). ν_{max}/ cm^{-1} 1410 (C-F, str), 1570 (C=C, w), 2960 (C-H, w), 3365 (C-H in Ph, m).

2-1-5. Reaction between $^{n}Bu_3Si$(*E*-CF=CFCF$_3$) (a.2) and ^{n}BuLi

$^{n}Bu_3Si$(*E*-CF=CFCF$_3$) (a.2) (1.3 g, 4.0 mmol) was dissolved in dry THF (150 mL), ^{n}BuLi (2.5 M, 3.2 mL, 8.0 mmol) was added slowly, the mixture was heated to reflux for 6 h. After work up using (G.P2), the product was isolated as a yellow liquid. Yield (0.5 g, 1.4 mmol, 46 %); ratio *Z*:*E* [$^{n}Bu_3Si$(*Z*-CF=C^{n}BuCF$_3$) (16*Z*) 88 : $^{n}Bu_3Si$(*E*-C^{n}Bu=CFCF$_3$) (16*E*) 12]. Anal.Calcd for $C_{19}H_{36}F_4Si$: C, 61.91; H, 9.85 %. Found: C, 61.72; H, 9.68 %. $^{19}F\{^{1}H\}$ NMR (376 MHz, $CDCl_3$, 291 K) δ ppm: $^{n}Bu_3Si$(*Z*-CF=C^{n}BuCF$_3$) −60.18 (d, 3F, $^{4}J(CF_3\ F_{gem})$ = 8.4 Hz, CF_3), −98.95 (q, 1F, $^{4}J(F_{gem}\ CF_3)$ = 8.5 Hz, F_{gem}) and $^{n}Bu_3Si$(*E*-C^{n}Bu=CFCF$_3$) −67.22 (d, 3F, $^{3}J(CF_3\ F_{trans})$ = 10.6 Hz, CF_3), −106.90 (q, 1F, $^{4}J(F_{trans}\ CF_3)$ = 10.9 Hz, F_{trans}). ^{1}H NMR (400 MHz, $CDCl_3$, 291 K) δ ppm: $^{n}Bu_3Si$(*Z*-CF=C^{n}BuCF$_3$) and $^{n}Bu_3Si$(*E*-C^{n}Bu=CFCF$_3$) 0.42 (m, 6H, CH_2-Si), 0.53 (m, 6H, CH_2-Si), 0.81-0.94 (m, 24H -CH_3), 1.23-1.55 (m, 32H, -CH_2CH_2-), 2.50-2.81 (m, 4H, CH_2-C=). $^{13}C\{^{1}H\}$ NMR (100 MHz, $CDCl_3$, 298 K) δ ppm: $^{n}Bu_3Si$(*Z*-CF=C^{n}BuCF$_3$) and $^{n}Bu_3Si$(*E*-C^{n}Bu=CFCF$_3$): 11.30 (s, Si-CH_2), 11.67 (s, Si-CH_2), 12.62 (s, =C-$\underline{C}H_2$-), 12.91(s, =C-$\underline{C}H_2$-), 13.11 (s, Si-C_3H_6-$\underline{C}H_3$), 13.23 (s, Si-C_3H_6-$\underline{C}H_3$), 13.93 (s, =C-C_3H_6-$\underline{C}H_3$), 14.27 (s, =C-C_3H_6-$\underline{C}H_3$), 24.30-24.90 (overlab, -$\underline{C}H_2\underline{C}H_2$-), 25.35 (s, Si-$C_2H_4$-$\underline{C}H_2$-$CH_3$), 25.85 (s, Si-$C_2H_4$-$\underline{C}H_2$-$CH_3$), 26.01 (s, Si-$CH_2$-$\underline{C}H_2$), 26.31 (s, Si-$CH_2$-$\underline{C}H_2$), $^{n}Bu_3Si$(*Z*-CF=C^{n}BuCF$_3$) 124.50 (q.d, $^{1}J(C_3\ 3F)$ = 271.4 Hz, $^{3}J(C_3\ F_{gem})$ = 24.1 Hz), 126.90 (q.d, $^{2}J(C_2\ 3F)$ = 28.8 Hz, $^{3}J(C_2\ F_{gem})$ = 18.3 Hz), 172.0 (d.q, $^{1}J(C_1\ F_{gem})$ = 288.2 Hz, $^{3}J(C_1\ 3F)$ = 6.5 Hz); $^{n}Bu_3Si$(*E*-C^{n}Bu=CFCF$_3$) 118.0 (q.d, $^{1}J(C_3\ 3F)$ = 272.2 Hz, $^{2}J(C_3\ F_{gem})$ = 50.5 Hz), 120.40 (m ,C_1), 147.0 (m, C_2). $^{29}Si\{^{1}H\}$ NMR (79 MHz, $CDCl_3$, 298 K) δ ppm: $^{n}Bu_3Si$(*Z*-CF=C^{n}BuCF$_3$) 2.10 (d, $^{3}J(Si\ F_{gem})$ = 31.7 Hz); $^{n}Bu_3Si$(*E*-C^{n}Bu=CFCF$_3$) 0.93 (d, $^{3}J(Si\ F_{trans})$ = 9.3 Hz). ν_{max}/ cm^{-1} 1260 (Si-C, m), 1375, 1465 (C-F, str), 1595 (C=C,w), 2950, 2920, 2870, 2860 (C-H, m).

2-1-6. Reaction between $^{n}Bu_3Si$(*E*-CF=CFCF$_3$) (a.2) and ^{t}BuLi

$^{n}Bu_3Si$(*E*-CF=CFCF$_3$) (a.2) (1.3 g, 4.0 mmol) was dissolved in dry THF (150 mL), ^{t}BuLi (1.7 M, 4.7 mL, 8.0 mmol) was added slowly. The product was isolated as a brown liquid after work up using (G.P2). Yield (0.6 g, 1.6 mmol, 53 %). Anal.Calcd for $C_{19}H_{36}F_4Si$: C, 61.91; H, 9.85 %. Found: C, 61.88; H, 9.68 %. $^{19}F\{^{1}H\}$ NMR (376 MHz, $CDCl_3$, 291 K) δ ppm: −55.55 (d, 3F, $^{4}J(CF_3\ F_{gem})$ = 7.1 Hz, CF_3), −86.66 (q, 1F, $^{4}J(F_{gem}\ CF_3)$ = 7.2 Hz, F_{gem}). ^{1}H NMR (400 MHz, $CDCl_3$, 291 K) δ ppm: 0.44 (t, 6H, $^{3}J(CH_2\ CH_2)$ = 9.7 Hz, Si-CH_2). 0.81 (t, 9H, $^{3}J(CH_3\ CH_2)$ = 9.3 Hz, CH_3), 1.24 (m, 12H, CH_2CH_2), 1.20 (s, 9H, C-$(CH_3)_3$). $^{13}C\{^{1}H\}$ NMR (100 MHz, $CDCl_3$, 298 K) δ ppm: 12.02 (s, Si-CH_2), 13.64 (s, $\underline{C}$-$(CH_3)_3$), 13.76 (s, Si-C_3H_6-$\underline{C}H_3$), 15.46 (s, C-$(\underline{C}H_3)_3$), 25.86 (s, Si-CH_2-$\underline{C}H_2$), 26.67 (s, CH_2-$\underline{C}H_3$), 125.00 (q.d, $^{1}J(C_3\ 3F)$ = 270.9 Hz, $^{3}J(C_3\ F_{gem})$ = 24.1 Hz, 3F, C_3), 126.90 (q.d, $^{2}J(C_2\ 3F)$ = 27.5 Hz, $^{2}J(C_2\ 3F)$ = 18.5 Hz, C_2), 173.17 (d.q, $^{1}J(C_1\ F_{gem})$ = 288.8 Hz, $^{3}J(C_1\ 3F)$ = 6.4 Hz). $^{29}Si\{^{1}H\}$ NMR (79 MHz, $CDCl_3$, 298 K) δ ppm: 2.15 (d, $^{2}J(Si\ F_{gem})$ = 31.1 Hz). ν_{max}/ cm^{-1} 1230 (Si-C, m), 1305, 1465 (C-F, str), 1595 (C=C, w), 2955, 2920, 2870, 2860, (C-H, m).

2-1-7. Reaction between $^{n}Bu_3Si$(*E*-CF=CFCF$_3$) (a.2) and MeLi

$^{n}Bu_3Si$(*E*-CF=CFCF$_3$) (a.2) (1.0 g, 3.0 mmol) was dissolved in dry THF (150 mL), MeLi (1.6 M, 9.4 mL, 15.0 mmol) was added slowly. The product was isolated as a brown liquid after work up using (G.P2). Yield (0.2 g, 0.6 mmol, 20 %); ratio *Z*:*E* [$^{n}Bu_3Si$(*Z*-CF=CMeCF$_3$) (18*Z*) 83 : $^{n}Bu_3Si$(*E*-CMe=CFCF$_3$) (18*E*) 17]. Anal.Calcd for $C_{16}H_{30}F_4Si$: C, 58.86; H, 9.27 %. Found: C, 58.38; H, 9.00 %. $^{19}F\{^{1}H\}$ NMR (376 MHz, $CDCl_3$, 291 K) δ ppm: $^{n}Bu_3Si$(*Z*-CF=CMeCF$_3$) −62.42 (d, 3F, $^{4}J(CF_3\ F_{gem})$ = 8.4 Hz, CF_3), −98.11 (q, 1F, $^{4}J(F_{gem}\ CF_3)$ = 8.5 Hz, F_{gem}), and $^{n}Bu_3Si$(*E*-CMe=CFCF$_3$) −67.06 (d, 3F, $^{3}J(CF_3\ F_{trans})$ = 10.6 Hz, CF_3), −105.64 (q, 1F, $^{3}J(F_{trans}\ CF_3)$ = 10.5 Hz, F_{trans}). ^{1}H NMR (400 MHz, $CDCl_3$, 291 K) δ ppm: $^{n}Bu_3Si$(*Z*-CF=CMeCF$_3$) and $^{n}Bu_3Si$(*E*-CMe=CFCF$_3$) 0.40 (m, 6H, Si-CH_2), 0.43 (m, 6H, Si-CH_2), 0.81(m, 9H, CH_3), 0.85 (m, 9H, CH_3), 1.24 (m, 12H, CH_2CH_2), 1.26 (m, 12H, CH_2CH_2), 3.33 (s, 3H, CH_3), 3.36 (s, 3H, CH_3). $^{13}C\{^{1}H\}$ NMR (100 MHz, $CDCl_3$, 298 K) δ ppm: $^{n}Bu_3Si$(*Z*-CF=CMeCF$_3$) and $^{n}Bu_3Si$(*E*-CMe=CFCF$_3$): 11.30 (s, C=C-$\underline{C}H_3$), 12.07 (s, C=C-$\underline{C}H_3$), 13.53 (s, Si-$\underline{C}H_2$), 13.75 (s, Si-$\underline{C}H_2$), 14.75 (s, $\underline{C}H_3$-CH_2), 15.41 (s, $\underline{C}H_3$-CH_2), 25.52 (s, Si-CH_2-$\underline{C}H_2$-), 26.18 (s, Si-CH_2-$\underline{C}H_2$-), 26.68 (s,

CH_3-$\underline{C}H_2$-CH_2), 26.79 (s, CH_3-$\underline{C}H_2$-CH_2), 26.79 (s, CH_3-$\underline{C}H_2$-CH_2), $^nBu_3Si$(*Z*-CF=CMeCF_3) 122.80 (m, C_2), 125.00 (q.d, ^1J(C_3 3F) = 270.6 Hz, ^3J(C_3 F_{gem}) = 23.9 Hz, C_3), 172.00 (d.q, ^1J(C_1 F_{gem}) = 287.9 Hz , ^3J(C_1 3F) = 6.6 Hz, C_1). $^nBu_3Si$(*E*-CMe=CFCF_3): 115.00 (m, C_3), 116.60 (d.q, ^3J(C_1 3F) = 3.5 Hz, ^2J(C_1 F_{trans}) = 8.4 Hz, C_1), 147.00 (d.q, ^1J(C_2 F_{trans}) = 266.7 Hz, ^2J(C_2 3F) = 39.8 Hz, C_2). $^{29}Si\{^1H\}$ NMR (79 MHz, $CDCl_3$, 298 K) δ ppm: $^nBu_3Si$(*Z*-CF=CMeCF_3) 2.00 (d, ^2J(Si F_{gem}) = 31.0 Hz); $^nBu_3Si$(*E*-CMe=CFCF_3) 0.90 (d, ^3J(Si F_{trans}) = 8.7 Hz). ν_{max}/ cm^{-1} 1260 (Si-C, m), 1375, 1465 (C-F, str), 1590 (C=C, w), 2955, 290, 2870, (C-H, m).

2-1-8. Reaction between $^nBu_3Si$(*E*-CF=CFCF_3) (a.2) and PhLi

$^nBu_3Si$(*E*-CF=CFCF_3) (a.2) (1.0 g, 3.0 mmol) was dissolved in dry THF (150 mL), PhLi (2.0 M, 7.5 mL, 15.0 mmol) was added slowly. The product was isolated as a brown liquid after work up using (G.P2). Yield (0.4 g, 1.0 mmol, 33 %); ratio *Z*:*E* [$^nBu_3Si$(*Z*-CF=CPhCF_3) (19*Z*) 69 : $^nBu_3Si$(*E*-CPh=CFCF_3) (19*E*) 31]. Anal.Calcd for $C_{21}H_{32}F_4Si$: C, 64.91; H, 8.31 %. Found: C, 64.86; H, 8.26 %. $^{19}F\{^1H\}$ NMR (376 MHz, $CDCl_3$, 291 K) δ ppm: $^nBu_3Si$(*Z*-CF=CPhCF_3) −58.51 (d, 3F, ^4J(CF_3 F_{gem}) = 9.1 Hz, CF_3), −93.02 (q, 1F, ^4J(F_{gem} CF_3) = 9.0 Hz, F_{gem}), and $^nBu_3Si$(*E*-CPh=CFCF_3) −67.67 (d, 3F, ^3J(CF_3 F_{trans}) = 10.8 Hz, CF_3), −100.34 (q, 1F, ^3J(F_{trans} CF_3) = 10.8 Hz, F_{trans}). ^1H NMR (400 MHz, $CDCl_3$, 291 K) δ ppm: $^nBu_3Si$(*Z*-CF=CPhCF_3) and $^nBu_3Si$(*E*-CPh=CFCF_3) 0.70 (t, 6H, ^3J(CH_2 CH_2) = 6.3 Hz, Si-CH_2), 0.79 (t, 6H, ^3J(CH_2 CH_2) = 6.5 Hz, Si-CH_2), 0.88 (m, 9H, -CH_3), 0.91 (m, 9H, -CH_3), 1.24 (m, 12H, -CH_2CH_2-), 1.63 (m, 12H, -CH_2CH_2-), 7.25-7.45 (m, 10H, Ph). $^{13}C\{^1H\}$ NMR (100 MHz, $CDCl_3$, 298 K) δ ppm: $^nBu_3Si$(*Z*-CF=CPhCF_3) and $^nBu_3Si$(*E*-CPh=CFCF_3) 11.44 (s, Si-$\underline{C}H_2$), 12.21 (s, Si-$\underline{C}H_2$), 13.62 (s, CH_3), 13.79 (s, CH_3), 25.83 (s, Si-CH_2-$\underline{C}H_2$-CH_2), 26.09 (s, Si-CH_2-$\underline{C}H_2$-CH_2-), 26.51 (s, CH_3-$\underline{C}H_2$-), 26.83 (s, CH_3-$\underline{C}H_2$-), 126.66 (s, Ph), 127.17 (s, Ph), 127.63 (s, Ph), 128.30 (s, Ph), 128.62 (s, Ph), 129.58 (s, Ph), 134.12 (s, Ph), 138.23 (s, Ph), $^nBu_3Si$(*Z*-CF=CPhCF_3) 124.00 (q.d, ^1J(C_3 3F) = 271.8 Hz, ^3J(C_3 F_{gem}) = 21.4 Hz, C_3), 124.6 (m, C_2), 173.00 (d.q, ^1J(C_1 F_{gem}) = 294.9 Hz, ^3J(C_1 3F) = 6.0 Hz, C_1). $^nBu_3Si$(*E*-CPh=CFCF_3): 117.20 (m, C_3), 136.20 (d, ^2J(C_2 F_{trans}) = 9.7 Hz), C_2), 147.50 (d.q, ^1J(C_1 F_{trans}) = 270.0 Hz, ^3J(C_1 3F) = 40.2 Hz, C_1). $^{29}Si\{^1H\}$ NMR (79 MHz, $CDCl_3$, 298 K) δ ppm: $^nBu_3Si$(*Z*-CF=CPhCF_3) 3.71 (d, ^2J(Si F_{gem}) = 30.6 Hz); $^nBu_3Si$(*E*-CPh=CFCF_3) −0.01 (d, ^3J(Si F_{trans}) = 5.4 Hz). ν_{max}/ cm^{-1} 1290 (Si-C, m), 1345, 1475 (C-F, str), 3060, 3030 (C-H in Ph, m), 2925 (C-H, m), 1690 (C=C, w).

2-1-9. Reaction between $^nBu(Me)_2Si$(*E*-CF=CFCF_3) (a.4) and ^nBuLi

$^nBu(Me)_2Si$(*E*-CF=CFCF_3) (3.4) (1.0 g, 4.0 mmol) was dissolved in dry THF (150 mL), ^nBuLi (2.5 M, 3.2 mL, 8.0 mmol) was added slowly. The product was isolated as a brown liquid after work up using (G.P2). Yield (0.6 g, 2.0 mmol, 50 %); ratio *Z*:*E* [$^nBu(Me)_2Si$(*Z*-CF=C$^nBu$$CF_3$) (20*Z*) 57 : $^nBu(Me)_2Si$(*E*-C^nBu=CFCF_3) (20*E*) 43]. Anal.Calcd for $C_{13}H_{24}F_4Si$: C, 54.90; H, 8.51 %. Found: C, 54.54; H, 8.47 %. $^{19}F\{^1H\}$ NMR (376 MHz, $CDCl_3$, 291 K) δ ppm: $^nBu(Me)_2Si$(*Z*-CF=C$^nBu$$CF_3$) −59.74 (d, 3F, ^4J(CF_3 F_{gem}) = 8.5 Hz, CF_3), −100.36 (q, 1F, ^4J(F_{gem} CF_3) = 8.6 Hz, F_{gem}) and $^nBu(Me)_2Si$(*E*-C^nBu=CFCF_3) −66.88 (d, 3F, ^3J(CF_3 F_{gem}) = 10.5 Hz, CF_3), −108.49 (q, 1F, ^3J(F_{gem} CF_3) = 10.4 Hz, F_{gem}). ^1H NMR (400 MHz, $CDCl_3$, 291 K) δ ppm: $^nBu(Me)_2Si$(*Z*-CF=C$^nBu$$CF_3$) and $^nBu(Me)_2Si$(*E*-C^nBu=CFCF_3): −0.15 (s, 6H, Si-CH_3), −0.11 (s, 6H, Si-CH_3), 0.01 (t, 2H, ^3J(CH_2 CH_2) = 6.6 Hz, Si-CH_2), 0.03 (t, 2H, ^3J(CH_2 CH_2) = 6.3 Hz, Si-CH_2), 0.32 (t, 3H, ^3J(CH_3 CH_2) = 7.0 Hz, SiC_3H_6-$C\underline{H_3}$), 0.52 (t, 3H, ^3J(CH_3 CH_2) = 6.9 Hz, SiC_3H_6-$C\underline{H_3}$), 0.73 (m, 3H, =CC_3H_6-$\underline{CH_3}$), 0.81 (m, 3H, =CC_3H_6-$\underline{CH_3}$), 1.14 (m, 4H, CH_2CH_2), 1.18 (m, 4H, CH_2CH_2), 1.27 (m, 4H, CH_2CH_2), 1.27 (m, 4H, CH_2CH_2), 2.14 (t, 2H, ^3J(CH_2 CH_2) = 7.3 Hz, CH_2-C=), 2.86 (t, 2H, ^3J(CH_2 CH_2) = 7.9 Hz, CH_2-C=). $^{13}C\{^1H\}$ NMR (100 MHz, $CDCl_3$, 298 K) δ ppm: $^nBu(Me)_2Si$(*Z*-CF=C$^nBu$$CF_3$) and $^nBu(Me)_2Si$(*E*-C^nBu=CFCF_3): −3.67 (s, Si-CH_3), −2.32 (s, Si-CH_3), 0.12 (s, Si-CH_2), 0.72 (s, Si-CH_2), 13.34 (s, =C-$\underline{C}H_2$), 13.37 (s, =C-$\underline{C}H_2$), 22.30 (s,CH_2-$\underline{C}H_3$), 22.40 (s,CH_2-$\underline{C}H_3$), 25.77 (s,CH_2-$\underline{C}H_3$), 26.19 (s,CH_2-$\underline{C}H_3$), 25.34 (s, CH_2-$\underline{C}H_2$-CH_2), 25.48 (s, CH_2-$\underline{C}H_2$-CH_2-), 26.04 (s, CH_2-$\underline{C}H_2$-CH_2-), 26.16 (s, CH_2-$\underline{C}H_2$-CH_2-), 27.61 (CH_2-$\underline{C}H_2$-CH_3), 27.48 (CH_2-$\underline{C}H_2$-CH_3), 30.56 (CH_2-$\underline{C}H_2$-CH_3), 31.33 (CH_2-$\underline{C}H_2$-CH_3), $^nBu(Me)_2Si$(*Z*-CF=C$^nBu$$CF_3$) 125.20 (q.d, ^1J(C_3 3F) = 272.7 Hz, ^3J(C_3 F_{gem}) = 23.1 Hz, C_3), 126.90 (q.d, ^2J(C_2 3F) = 28.7 Hz, ^2J(C_2 F_{gem}) = 18.3 Hz, C_2), 173.40 (d.q, ^1J(C_1 F_{gem}) = 288.0 Hz, ^3J(C_1 3F) = 6.0 Hz); $^nBu(Me)_2Si$(*E*-C^nBu=CFCF_3) 118.73 (q.d, ^1J(C_3 3F) = 271.8 Hz, ^1J(C_3 F_{trans}) = 48.2 Hz, C_3), 122.25 (d, ^2J(C_1 F_{trans}) = 8.7 Hz, C_1), 147.27 (d.q, ^1J(C_2 F_{trans}) = 287.9 Hz, ^2J(C_2 3F) = 40.9 Hz, C_2). $^{29}Si\{^1H\}$ NMR (79 MHz, $CDCl_3$, 298 K) δ ppm: $^nBu(Me)_2Si$(*Z*-CF=C$^nBu$$CF_3$) 3.71 (d, ^2J(Si F_{gem}) = 30.6 Hz); $^nBu(Me)_2Si$(*E*-C^nBu=CFCF_3) −0.01 (d, ^3J(Si F_{trans}) = 5.4 Hz). ν_{max}/ cm^{-1} 1295 (Si-C, m), 1315, 1465 (C-F, str), 1635 (C=C, w), 2955, 2920, 2870, 2860 (C-H, m).

2-1-10. Reaction between $^{n}Bu(Me)_2Si$(*E*-CF=CFCF$_3$) (a.4) and $^{t}BuLi$

$^{n}Bu(Me)_2Si$(*E*-CF=CFCF$_3$) (a.4) (1.0 g, 4.0 mmol) was dissolved in dry THF (150 mL), $^{t}BuLi$ (1.7 M, 4.7 mL, 8.0 mmol) was added slowly. After work up using (G.P2), the product was isolated as a brown liquid. Yield (0.5 g, 1.8 mmol, 45 %). Anal.Calcd for $C_{13}H_{24}F_4Si$: C, 54.90; H, 8.51 %. Found: C, 54.95; H, 8.94 %. $^{19}F\{^1H\}$ NMR (376 MHz, CDCl$_3$, 291 K) δ ppm: −55.20 (d, 3F, ^4J(CF$_3$ F) = 7.3 Hz, CF$_3$), −88.24 (q, 1F, ^4J(F CF$_3$) = 7.1 Hz, F$_{gem}$). ^1H NMR (400 MHz, CDCl$_3$, 291 K) δ ppm: −0.04 (s, 6H, Si-CH$_3$), 0.13 (t, 2H, ^3J(CH$_2$ CH$_2$) = 1.4 Hz, Si-CH$_2$), 0.82 (m, 4H, CH$_2$CH$_2$), 1.20 (s, 9H, C-CH$_3$). $^{13}C\{^1H\}$ NMR (100 MHz, CDCl$_3$, 298 K) δ ppm: −3.75 (s, Si-CH$_3$), 12.66 (s, Si-CH$_2$), 13.85 (s, =C-$\underline{C}$-CH$_3$), 24.84 (s, Si-C$_3$H$_6$-$\underline{C}$H$_3$), 25.25 (s, C-$\underline{C}$H$_3$), 28.95 (s, Si-CH$_2$-$\underline{C}$H$_2$), 33.87 (s, Si-C$_2$H$_4$-$\underline{C}$H$_2$-CH$_3$), 124.55 (q.d, ^1J(C$_3$ 3F) = 273.5 Hz, ^3J(C$_3$ F$_{gem}$) = 28.4 Hz, 3F, C$_3$), 132.01 (q.d, ^2J(C$_2$ 3F) = 24.8 Hz, ^2J(C$_2$ 3F) = 13.0 Hz, C$_2$), 174.98 (d.q, ^1J(C$_1$ F$_{gem}$) = 292.7 Hz, ^3J(C$_1$ 3F) = 9.2 Hz). $^{29}Si\{^1H\}$ NMR (79 MHz, CDCl$_3$, 298 K) δ ppm: −1.85 (d, ^2J(Si F$_{gem}$) = 37.2 Hz). ν_{max}/ cm^{-1} 1260 (Si-C, m), 1375, 1460 (C-F, str), 1600 (C=C, w) 2955, 2920, 2870, 2580 (C-H, m).

2-1-11. Reaction between $^{n}Bu(Me)_2Si$(*E*-CF=CFCF$_3$) (a.4) and MeLi

$^{n}Bu(Me)_2Si$(*E*-CF=CFCF$_3$) (a.4) (1.0 g, 4.0 mmol) was dissolved in dry THF (150 mL), MeLi (1.6 M, 12.5 mL, 20.0 mmol). After work up using (G.P2), the product was isolated as a brown liquid. Yield (0.4 g, 1.6 mmol, 40 %); ratio *Z*:*E* [$^{n}Bu(Me)_2Si$(*Z*-CF=CMeCF$_3$) (22*Z*) 40 : $^{n}Bu(Me)_2Si$(*E*-CMe=CFCF$_3$) (22*E*) 60]. Anal.Calcd for $C_{10}H_{18}F_4Si$: C, 49.56; H, 7.49 %. Found: C, 49.30; H, 7.44 %. $^{19}F\{^1H\}$ NMR (376 MHz, CDCl$_3$, 291 K) δ ppm: $^{n}Bu(Me)_2Si$(*Z*-CF=CMeCF$_3$) −61.59 (d, 3F, ^4J(CF$_3$ F$_{gem}$) = 8.3 Hz, CF$_3$), −99.39 (q, 1F, ^4J(F CF$_3$) = 8.5 Hz, F$_{gem}$) and $^{n}Bu(Me)_2Si$(*E*-CMe=CFCF$_3$) −66.65 (d, 3F, ^3J(CF$_3$ F$_{trans}$) = 10.2 Hz, CF$_3$), −107.12 (q, 1F, ^3J(F CF$_3$) = 10.5 Hz, F$_{trans}$). ^1H NMR (400 MHz, CDCl$_3$, 291 K) δ ppm: $^{n}Bu(Me)_2Si$(*Z*-CF=CMeCF$_3$) and $^{n}Bu(Me)_2Si$(*E*-CMe=CFCF$_3$) 0.10 (s, 6H, Si-CH$_3$), 0.14 (s, 6H, Si-CH$_3$), 0.43 (m, 2H, Si-CH$_2$), 0.63 (m, 2H, Si-CH$_2$), 0.81 (t, 3H, ^3J(CH$_3$ CH$_2$) = 7.2 Hz, CH$_3$), 0.82 (t, 3H, ^3J(CH$_3$ CH$_2$) = 7.1 Hz, CH$_3$), 1.19 (m, 4H, CH$_2$CH$_2$), 1.23 (m, 4H, CH$_2$CH$_2$), 1.76 (s, 3H, =C-CH$_3$), 1.75 (s, 3H, =C-CH$_3$). $^{13}C\{^1H\}$ NMR (100 MHz, CDCl$_3$, 298 K) δ ppm: $^{n}Bu(Me)_2Si$(*Z*-CF=CMeCF$_3$) and $^{n}Bu(Me)_2Si$(*E*-CMe=CFCF$_3$) −4.37 (s, Si-CH$_3$), −3.32 (s, Si-CH$_3$), 0.37 (s, =C-$\underline{C}$H$_3$), 0.74 (s, =C-$\underline{C}$H$_3$), 12.06 (s, Si-CH$_2$), 12.66 (s, Si-CH$_2$), 13.27 (s, Si-C$_3$H$_6$-$\underline{C}$H$_3$), 14.12 (s, Si-C$_3$H$_6$-$\underline{C}$H$_3$), 24.52 (s, Si-CH$_2$-$\underline{C}$H$_2$), 24.65 (s, Si-CH$_2$-$\underline{C}$H$_2$), 25.27 (s, Si-C$_2$H$_4$-$\underline{C}$H$_2$-CH$_3$), 25.38 (s, Si-C$_2$H$_4$-$\underline{C}$H$_2$-CH$_3$); $^{n}Bu(Me)_2Si$(*Z*-CF=CMeCF$_3$) 123.59 (q.d, ^1J(C$_3$ 3F) = 271.8 Hz, ^3J(C$_3$ F$_{gem}$) = 24.7 Hz, C$_3$), 121.45 (m, C$_2$), 171.93 (d.q, ^1J(C$_1$ F$_{gem}$) = 286.6 Hz, ^3J(C$_1$ 3F) = 6.6 Hz, C$_1$), $^{n}Bu(Me)_2Si$(*E*-CMe=CFCF$_3$) 117.93 (q.d, ^1J(C$_3$ 3F) = 274.5 Hz, ^2J(C$_3$ F$_{trans}$) = 50.1 Hz, C$_3$), 116.89 (m, C$_1$), 146.03 (d.q, ^1J(C$_2$ F$_{trans}$) = 227.3 Hz, ^2J(C$_2$ 3F) = 40.0 Hz, C$_2$). $^{29}Si\{^1H\}$ NMR (79 MHz, CDCl$_3$, 298 K) δ ppm: $^{n}Bu(Me)_2Si$(*Z*-CF=CMeCF$_3$) −21.95 (d, ^2J(Si F$_{gem}$) = 37.0 Hz); $^{n}Bu(Me)_2Si$(*E*-CMe=CFCF$_3$) 7.25 (d, ^3J(Si F$_{trans}$) = 5.6 Hz). ν_{max}/ cm^{-1} 1250 (Si-C, w), 1300, 1445 (C-F, str), 1600 (C=C,w), 2940, 2830 (C-H, m).

2-1-12. Reaction between Me_2PhSi(*E*-CF=CFCF$_3$) (a.5) and $^{n}BuLi$

Me_2PhSi(*E*-CF=CFCF$_3$) (a.5) (1.0 g, 4.0 mmol) was dissolved in dry THF (150 mL), $^{n}BuLi$ (2.5 M, 3.2 mL, 8.0 mmol) was added slowly. After work up using (G.P2), the product was isolated as a brown liquid. Yield (0.6 g, 2.9 mmol, 66 %); ratio *Z*:*E* [Me_2PhSi(*Z*-CF=C^{n}BuCF$_3$) (23*Z*) 55 : Me_2PhSi(*E*-C^{n}Bu=CFCF$_3$) (23*E*) 45]. Anal.Calcd for $C_{15}H_{20}F_4Si$: C, 59.19; H, 6.63 %. Found: C, 59.17; H, 6.87 %. $^{19}F\{^1H\}$ NMR (376 MHz, CDCl$_3$, 291 K) δ ppm: Me_2PhSi(*Z*-CF=C^{n}BuCF$_3$) −59.38 (d, 3F, ^4J(CF$_3$ F$_{gem}$) = 8.2 Hz, CF$_3$), −99.54 (q, 1F, ^4J(F$_{gem}$ CF$_3$) = 8.6 Hz, F$_{gem}$) and Me_2PhSi(*E*-C^{n}Bu=CFCF$_3$) −66.48 (d, 3F, ^3J(CF$_3$ F$_{trans}$) = 10.0 Hz, CF$_3$), −107.00 (q, 1F, ^3J(F$_{trans}$ CF$_3$) = 10.2 Hz, F$_{trans}$). ^1H NMR (400 MHz, CDCl$_3$, 291 K) δ ppm: 0.17 (s, 3H, Si-CH$_3$), 0.25 (s, 3H, Si-CH$_3$), 0.39 (t, 3H, ^3J(CH$_3$ CH$_2$) = 0.8 Hz, CH$_3$), 0.43 (t, 3H, ^3J(CH$_3$ CH$_2$) = 0.9 Hz, CH$_3$), 0.74 (m, 4H, CH$_2$CH$_2$), 0.83 (m, 4H, CH$_2$CH$_2$), 2.13 (t, 2H, ^3J(CH$_2$ CH$_3$) = 8.3 Hz, =C-CH$_2$), 2.39 (t, 2H, ^3J(CH$_2$ CH$_3$) = 8.5 Hz, =C-CH$_2$), 7.41 (m, 5H, Ph), 7.45 (m, 5H, Ph). $^{13}C\{^1H\}$ NMR (100 MHz, CDCl$_3$, 298 K) δ ppm: Me_2PhSi(*Z*-CF=C^{n}BuCF$_3$) and Me_2PhSi(*E*-C^{n}Bu=CFCF$_3$) −3.02 (s, Si-CH$_3$), −4.38 (s, Si-CH$_3$), 12.65 (s, =C-$\underline{C}$H$_2$), 12.78 (s, =C-$\underline{C}$H$_2$), 21.52 (s, =C-C$_3$H$_6$-$\underline{C}$H$_3$), 21.63 (s, =C-C$_3$H$_6$-$\underline{C}$H$_3$), 24.54 (s, =C-C$_2$H$_2$-$\underline{C}$H$_2$-), 24.69 (s, =C-C$_2$H$_2$-$\underline{C}$H$_2$-), 25.26 (s, =C-CH$_2$-$\underline{C}$H$_2$-), 25.41 (s, =C-CH$_2$-$\underline{C}$H$_2$-), 126.70 (s, Ph), 126.83 (s, Ph), 128.17 (s, Ph), 128.29 (s, Ph), 132.11 (s, Ph), 132.35 (s, Ph), 138.45 (s, Ph), 138.80 (s, Ph); Me_2PhSi(*Z*-CF=C^{n}BuCF$_3$): 124.34 (q.d, ^1J(C$_3$ 3F) = 270.3 Hz, ^3J(C$_3$ F$_{gem}$) = 24.9 Hz, C$_3$), 126.22 (m, C$_2$), 172.71 (d.q, ^1J(C$_1$ F$_{gem}$) = 288.5 Hz, ^3J(C$_1$ 3F) = 7.5 Hz, C$_1$), Me_2PhSi(*E*-C^{n}Bu=CFCF$_3$), 117.91 (q.d, ^1J(C$_3$ 3F) = 270.8 Hz, ^2J(C$_3$ F$_{trans}$) = 49.6 Hz, C$_3$), 121.61 (m, C$_1$), 146.46 (d.q, ^1J(C$_2$ F$_{trans}$) = 267.8 Hz, ^2J(C$_2$ 3F) = 39.4 Hz, C$_2$). $^{29}Si\{^1H\}$ NMR (79 MHz, CDCl$_3$,

298 K) δ ppm: Me_2PhSi(*Z*-CF=C^{n}BuCF$_3$) −7.70 (d, ^2J(Si F$_{gem}$) = 37.8 Hz); Me_2PhSi(*E*-C^{n}Bu=CFCF$_3$) −6.22 (d, ^3J(Si F$_{trans}$) = 11.5 Hz). ν_{max}/ cm^{-1} 1250 (Si-C, w), 1320, 1460 (C-F, str), 1640 (C=C, w), 2960, 2870 (C-H, m), 3010 (C-H in Ph, w).

2-1-13. Reaction between $Me_2PhSi(CF=CFCF_3)$ (a.5) and ^{t}BuLi

Me_2PhSi(*E*-CF=CFCF$_3$) (a.5) (1.0 g, 4.0 mmol) was dissolved in dry THF (150 mL), ^{t}BuLi (1.7 M, 4.7 mL, 8.0 mmol) was added slowly. After work up using (G.P2), the product was isolated as a brown liquid. Yield (0.3 g, 0.9 mmol, 33 %); ratio *Z*:*E* [Me_2PhSi(*Z*-CF=C^{t}BuCF$_3$) (24*Z*) 72 : Me_2PhSi(*E*-C^{t}Bu=CFCF$_3$) (24*E*) 28]. Anal.Calcd for $C_{15}H_{20}F_4Si$: C, 59.19; H, 6.63 %. Found: C, 59.00; H, 6.55 %. $^{19}F\{^1H\}$ NMR (376 MHz, $CDCl_3$, 291 K) δ ppm: Me_2PhSi(*Z*-CF=C^{t}BuCF$_3$) −59.17 (d, 3F, ^4J(CF$_3$ F$_{gem}$) = 8.2 Hz, CF$_3$), −99.55 (q, 1F, ^4J(F CF$_3$) = 9.0 Hz, F$_{gem}$) and Me_2PhSi(*E*-C^{t}Bu=CFCF$_3$) −66.27 (d, 3F, ^3J(CF$_3$ F$_{trans}$) = 10.3 Hz, CF$_3$), −106.72 (q, 1F, ^3J(F CF$_3$) = 10.9 Hz, F$_{trans}$). ^1H NMR (400 MHz, $CDCl_3$, 291 K) δ ppm: Me_2PhSi(*Z*-CF=C^{t}BuCF$_3$) and Me_2PhSi(*E*-C^{t}Bu=CFCF$_3$) 0.26 (s, 6H, Si-CH$_3$), 0.27 (s, 6H, Si-CH$_3$), 1.19 (s, 9H, C-CH$_3$), 1.20 (s, 9H, C-CH$_3$), 7.48 (s, 5H, Ph), 7.49 (s, 5H, Ph). $^{13}C\{^1H\}$ NMR (100 MHz, $CDCl_3$, 298 K) δ ppm: Me_2PhSi(*Z*-CF=C^{t}BuCF$_3$) and Me_2PhSi(*E*-C^{t}Bu=CFCF$_3$) −0.80 (s, Si-CH$_3$), −0.93 (s, Si-CH$_3$), 21.10 (s,=C-$\underline{C}$-CH$_3$), 22.26 (s,=C-$\underline{C}$-CH$_3$), 31.21 (s,=C-C-$\underline{C}$H$_3$), 33.94 (s,=C-C-$\underline{C}$H$_3$), 126.76 (s, Ph), 126.85 (s, Ph), 128.39 (s, Ph), 128.18 (s, Ph), 132.10 (s, Ph), 132.45 (s, Ph), 138.48 (s, Ph), 138.83 (s, Ph). Me_2PhSi(*Z*-CF=C^{t}BuCF$_3$) 122.33 (q.d, ^1J(C$_3$ 3F) = 285.7 Hz, ^3J(C$_3$ F$_{gem}$) = 22.1 Hz, C$_3$), 139.72 (m, C$_2$), 156.99 (d.q, ^1J(C$_1$ F$_{gem}$) = 208.6 Hz, ^3J(C$_1$ 3F) = 250.7 Hz, C$_1$), Me_2PhSi(*E*-C^{t}Bu=CFCF$_3$) 107.00 (q.d, ^1J(C$_3$ 3F) = 281.6 Hz, ^2J(C$_3$ F$_{trans}$) = 50.3 Hz, C$_3$), 119.00 (m, C$_1$), 145.35 (d.q, ^1J(C$_2$ F$_{trans}$) = 201.3 Hz, ^2J(C$_2$ 3F) = 49.0 Hz, C$_2$). $^{29}Si\{^1H\}$ NMR (79 MHz, $CDCl_3$, 298 K) δ ppm: Me_2PhSi(*Z*-CF=C^{t}BuCF$_3$) −2.45 (d, ^2J(Si F$_{gem}$) = 38.0 Hz); Me_2PhSi(*E*-C^{t}Bu=CFCF$_3$) −1.14 (d, ^3J(Si F$_{trans}$) = 6.6 Hz). ν_{max}/cm^{-1} 1255 (Si-C, w), 1320, 1430 (C-F, str), 1645 (C=C, w), 2960, 2870 (C-H, m).

2-1-14. Reaction between Me_2PhSi(*E*-CF=CFCF$_3$) (a.5) and MeLi

Me_2PhSi(*E*-CF=CFCF$_3$) (a.5) (1.0 g, 3.0 mmol) was dissolved in dry THF (150 mL), MeLi (1.6 M, 9.0 mL, 15.0 mmol) was added slowly. After work up using (G.P2), the product was isolated as a brown liquid. Yield (0.3 g, 1.1 mmol, 36 %); ratio *Z*:*E* [Me_2PhSi(*Z*-CF=CMeCF$_3$) (25*Z*) 72 : Me_2PhSi(*E*-CMe=CFCF$_3$) (25*E*) 28]. Anal.Calcd for $C_{12}H_{14}F_4Si$: C, 54.95; H, 5.38 %. Found: C, 54.63; H, 5.05 %. $^{19}F\{^1H\}$ NMR (376 MHz, $CDCl_3$, 291 K) δ ppm: Me_2PhSi(*Z*-CF=CMeCF$_3$) −60.17 (d, 3F, ^4J(CF$_3$ F) = 8.2 Hz, CF$_3$), −98.95 (q, 1F, ^4J(F CF$_3$) = 8.7 Hz, F$_{gem}$) and Me_2PhSi(*E*-CMe=CFCF$_3$) −67.20 (d, 3F, ^3J(CF$_3$ F) = 10.5 Hz, CF$_3$), −106.91 (q, 1F, ^3J(F CF$_3$) = 10.9 Hz, F$_{trans}$). ^1H NMR (400 MHz, $CDCl_3$, 291 K) δ ppm: Me_2PhSi(*Z*-CF=CMeCF$_3$) and Me_2PhSi(*E*-CMe=CFCF$_3$) 0.20 (s, 6H, Si-CH$_3$), 0.23 (s, 6H, Si-CH$_3$), 1.76 (s, 3H, =C-CH$_3$), 1.75 (s, 3H, =C-CH$_3$), 7.56 (m, 5H, Ph), 7.65 (m, 5H, Ph). $^{13}C\{^1H\}$ NMR (100 MHz, $CDCl_3$, 298 K) δ ppm: Me_2PhSi(*Z*-CF=CMeCF$_3$) and Me_2PhSi(*E*-CMe=CFCF$_3$) −4.28 (s, Si-CH$_3$), −0.88 (s, Si-CH$_3$), 12.92 (s,=C-$\underline{C}$H$_3$), 14.55 (s,=C-$\underline{C}$H$_3$), 126.80 (s, Ph), 127.02 (s, Ph), 127.35 (s, Ph), 127.26 (s, Ph), 128.16 (s, Ph), 128.37 (s, Ph), 137.00 (s, Ph), 138.91 (s, Ph), C$_1$, C$_2$ and C$_3$ in Me_2PhSi(*Z*-CF=CMeCF$_3$) and Me_2PhSi(*E*-CMe=CFCF$_3$) were not observed. $^{29}Si\{^1H\}$ NMR (79 MHz, $CDCl_3$, 298 K) δ ppm: Me_2PhSi(*Z*-CF=CMeCF$_3$) 6.10 (d, ^2J(Si F$_{gem}$) = 37.4 Hz); Me_2PhSi(*E*-CMe=CFCF$_3$) 1.88 (d, ^3J(Si F$_{trans}$) = 5.6 Hz). ν_{max}/ cm^{-1} 1295 (Si-C, w), 1375, 1465 (C-F, str), 1650 (C=C, w), 2955, 2920, 2870, 2855 (C-H, m).

2-1-15. Reaction between Ph_2MeSi(*E*-CF=CFCF$_3$) (a.6) and ^{n}BuLi

Ph_2MeSi(*E*-CF=CFCF$_3$) (a.6) (1.3 g, 4.0 mmol) was dissolved in dry THF (150 mL), ^{n}BuLi (2.5 M, 3.2 mL, 8.0 mmol) was added slowly. After work up using (G.P2), the product was isolated as a brown liquid. Yield (0.8 g, 2.2 mmol, 73 %); ratio *Z*:*E* [Ph_2MeSi(*Z*-CF=C^{n}BuCF$_3$) (26*Z*) 70 : Ph_2MeSi(*E*-C^{n}Bu=CFCF$_3$) (26*E*) 30]. Anal.Calcd for $C_{20}H_{22}F_4Si$: C, 65.55; H, 6.06 %. Found: C, 65.93; H, 6.21 %. $^{19}F\{^1H\}$ NMR (376 MHz, $CDCl_3$, 291 K) δ ppm: Ph_2MeSi(*Z*-CF=C^{n}BuCF$_3$) −59.31 (d, 3F, ^4J(CF$_3$ F$_{gem}$) = 8.3 Hz, CF$_3$), −97.03 (q, 1F, ^4J(F$_{gem}$ CF$_3$) = 8.5 Hz, F$_{gem}$) and Ph_2MeSi(*E*-C^{n}Bu=CFCF$_3$) −66.65 (d, 3F, ^3J(CF$_3$ F$_{trans}$) = 10.4 Hz, CF$_3$), −105.47 (q, 1F, ^3J (F$_{trans}$ CF$_3$) = 10.3 Hz, F$_{trans}$). ^1H NMR (400 MHz, $CDCl_3$, 291 K) δ ppm: Ph_2MeSi(*Z*-CF=C^{n}BuCF$_3$) and Ph_2MeSi(*E*-C^{n}Bu=CFCF$_3$): 0.46 (s, 3H, Si-CH$_3$), 0.49 (s, 3H, Si-CH$_3$), 0.58 (t, 3H, ^3J(CH$_3$ CH$_2$) = 6.8 Hz, CH$_3$), 0.84 (t, 3H, ^3J(CH$_3$ CH$_2$) = 7.2 Hz, CH$_3$), 1.28 (m, 4H, CH$_2$CH$_2$), 1.43 (m, 4H, CH$_2$CH$_2$), 3.32 (t, 2H, ^3J(CH$_2$ CH$_2$) = 6.7 Hz, =C-CH$_2$), 3.50 (t, 2H, ^3J(CH$_2$ CH$_2$) = 6.5 Hz, =C-CH$_2$), 7.30-7.75 (m, 20H, Ph). $^{13}C\{^1H\}$ NMR (100 MHz, $CDCl_3$, 298 K) δ ppm: Ph_2MeSi(*Z*-CF=C^{n}BuCF$_3$) and Ph_2MeSi(*E*-C^{n}Bu=CFCF$_3$): −3.88 (s, Si-CH$_3$), −3.89 (s, Si-CH$_3$), 13.92 (s, =C-$\underline{C}$H$_2$), 14.54 (s, =C-$\underline{C}$H$_2$), 23.03 (s, Si-C$_3$H$_6$-$\underline{C}$H$_3$), 23.19 (s, Si-C$_3$H$_6$-$\underline{C}$H$_3$), 26.66 (s, Si-CH$_2\underline{C}$H$_2$-C$_2$H$_5$), 27.22 (s, Si-CH$_2\underline{C}$H$_2$-C$_2$H$_5$), 28.95 (s, Si-C$_2$H$_4\underline{C}$H$_2$-CH$_3$), 29.07 (s, Si-C$_2$H$_4\underline{C}$H$_2$-CH$_3$), 128.35 (s, Ph), 128.53 (s, Ph), 129.61 (s, Ph), 130.51 (s, Ph), 133.73 (s, Ph), 134.58 (s, Ph), 135.03 (s, Ph), 135.35 (s, Ph), Ph_2MeSi(*Z*-CF=C^{n}BuCF$_3$) 125.48 (q.d, ^1J(C$_3$ 3F) = 271.4 Hz,

^3J(C_3 F_{gem}) = 22.8 Hz, C_3), 139.31 (m, C_2), 170.98 (d.q, ^1J(C_1 F_{gem}) = 285.0 Hz, ^3J(C_1 3F) = 6.0 Hz, C_1), Ph_2MeSi(*E*-C^nBu=CFCF_3) 119.12 (q.d, ^1J(C_3 3F) = 275.6 Hz, ^2J(C_3 F_{trans}) = 49.3 Hz, C_3), 138.11 (m, C_1), 149.70 (d.q, ^1J(C_2 F_{trans}) = 269.6 Hz, ^2J(C_2 3F) = 40.5 Hz, C_2). ^{29}Si{^{1}H} NMR (79 MHz, $CDCl_3$, 298 K) δ ppm: Ph_2MeSi(*Z*-CF=C^nBuCF_3) −12.60 (d, ^2J(Si F_{gem}) = 38.0 Hz); Ph_2MeSi(*E*-C^nBu=CFCF_3): −9.34 (d, ^3J(Si F_{trans}) = 12.9 Hz). ν_{max}/ cm^{-1} 1260 (Si-C, m), 1320, 1430 (C-F, str), 1635 (C=C, w), 2960, 2930, 2870 (C-H, m), 3070 (C-H in Ph, w).

2-1-16. Reaction between Ph_2MeSi(*E*-CF=CFCF_3) (a.6) and ^{t}BuLi

Ph_2MeSi(*E*-CF=CFCF_3) (a.6) (1.3 g, 4.0 mmol) was dissolved in dry THF (150 mL), ^{t}BuLi (1.7 M, 4.7 mL, 8.0 mmol) was added slowly. After work up using (G.P2), the product was isolated as a yellow liquid. Yield (0.5 g, 1.4 mmol, 46 %). Anal.Calcd for $C_{20}H_{22}F_4Si$: C, 65.55; H, 6.06 %. Found: C, 65.54; H, 6.54 %. ^{19}F{^{1}H} NMR (376 MHz, $CDCl_3$, 291 K) δ ppm: −54.77 (d, 3F, ^4J(CF_3 F_{gem}) = 6.7 Hz, CF_3), −84.52 (q, 1F, ^4J(F_{gem} CF_3) = 6.7 Hz, F_{gem}). ^{1}H NMR (400 MHz, $CDCl_3$, 291 K) δ ppm: 0.49 (s, 3H, Si-CH_3), 1.24 (s, 9H, C-CH_3), 7.30-7.42 (m, 10H, Ph). ^{13}C{^{1}H} NMR (100 MHz, $CDCl_3$, 298 K) δ ppm: −2.05 (s, Si-CH_3), 30.50 (s, =C-<u>C</u>-CH_3), 35.82 (s, =C-C-<u>C</u>H_3), 125.57 (q.d, ^1J(C_3 3F) = 274.3 Hz, ^3J(C_3 F_{gem}) = 26.8 Hz, 3F, C_3), 128.39 (s, Ph), 129.73 (s, Ph), 134.59 (s, Ph), 135.10 (s, Ph), 139.14 (q.d ,^2J(C_2 3F) = 25.3 Hz, ^2J(C_2 3F) = 12.8 Hz, C_2), 172.78 (d.q, ^1J(C_1 F_{gem}) = 290.2 Hz, ^3J(C_1 3F) = 8.7 Hz, C_1). ^{29}Si{^{1}H} NMR (79 MHz, $CDCl_3$, 298 K) δ ppm: −10.03 (d, ^2J(Si F_{gem}) = 37.5 Hz). ν_{max}/ cm^{-1} 1255 (Si-C, m), 1360, 1425(C-F, str), 1600 (C=C, w), 2965 (C-H, w), 3075 (C-H in Ph, w).

2-1-17. Reaction between Ph_2MeSi(*E*-CF=CFCF_3) (3.6) and MeLi

Ph_2MeSi(*E*-CF=CFCF_3) (a.6) (1.0 g, 3.0 mmol) was dissolved in dry THF (150 mL), MeLi (1.6 M, 9 mL, 15 mmol) was added slowly. After work up using (G.P2), the product was isolated as a brown liquid. Yield (0.6 g, 1.9 mmol, 63 %); ratio *Z*:*E* [Ph_2MeSi(*Z*-CF=CMeCF_3) (28*Z*) 52 : Ph_2MeSi(*E*-CMe=CFCF_3) (28*E*) 48]. Anal.Calcd for $C_{17}H_{16}F_4Si$: C, 62.94; H, 4.98 %. Found: C, 62.55; H, 4.63 %. ^{19}F{^{1}H} NMR (376 MHz, $CDCl_3$, 291 K) δ ppm: Ph_2MeSi(*Z*-CF=CMeCF_3) −61.46 (d, 3F, ^4J(CF_3 F_{gem}) = 7.9 Hz, CF_3), −96.13 (q, 1F, ^4J(F CF_3) = 7.7 Hz, F_{gem}) and Ph_2MeSi(*E*-CMe=CFCF_3) −66.37 (d, 3F, ^3J(CF_3 F_{trans}) = 10.4 Hz, CF_3), −103.41 (q, 1F, ^3J(F_{trans} CF_3) = 10.7 Hz, F_{trans}). ^{1}H NMR (400 MHz, $CDCl_3$, 291 K) δ ppm: Ph_2MeSi(*Z*-CF=CMeCF_3) and Ph_2MeSi(*E*-CMe=CFCF_3): 0.18 (s, 3H, Si-CH_3), 0.25 (s, 3H, Si-CH_3), 0.47 (s, 3H, =C-CH_3), 0.49 (s, 3H, =C-CH_3), 7.30-7.60 (m, 20H, Ph). ^{13}C{^{1}H} NMR (100 MHz, $CDCl_3$, 298 K) δ ppm: Ph_2MeSi(*Z*-CF=CMeCF_3) and Ph_2MeSi(*E*-CMe=CFCF_3): −0.91(s, Si-CH_3), −0.27 (s, Si-CH_3), 1.25 (s, =C-<u>C</u>H_3), 1.84 (s, =C-<u>C</u>H_3), 128.54 (s, Ph), 129.00 (s, Ph), 130.11 (s, Ph), 130.18 (s, Ph), 131.46 (s, Ph), 131.95 (s, Ph), 139.94 (s, Ph), 140.55 (s, Ph), Ph_2MeSi(*Z*-CF=CMeCF_3) 126.35 (q.d, ^1J(C_3 3F) = 213.0 Hz, ^3J(C_3 F_{gem}) = 21.9 Hz, C_3), 141.84 (m, C_2), 171.82 (d.q, ^1J(C_1 F_{gem}) = 171.8 Hz, ^3J(C_1 3F) = 7.0 Hz, C_1), Ph_2MeSi(*E*-CMe=CFCF_3): 120.60 (q.d, ^1J(C_3 3F) = 275.6 Hz, ^2J(C_3 F_{trans}) = 49.3 Hz, C_3), 135.65 (m, C_1), 149.70 (d.q, ^1J(C_2 F_{trans}) = 278.3 Hz, ^2J(C_2 3F) = 47.3 Hz, C_2). ^{29}Si{^{1}H} NMR (79 MHz, $CDCl_3$, 298K) δ ppm: Ph_2MeSi(*Z*-CF=CMeCF_3) −21.19 (d, ^2J(Si F_{gem}) = 37.3 Hz); Ph_2MeSi(*E*-CMe=CFCF_3): −10.99 (d, ^3J(Si F_{trans}) = 5.3 Hz). ν_{max}/ cm^{-1} 1250 (Si-C, m), 1365, 1435 (C-F, str), 1600 (C=C, w), 3070 (C-H in Ph, w), 2960 (C-H, w).

2-1-18. Reaction between Ph_2MeSi(*E*-CF=CFCF_3) (a.6) and PhLi

Ph_2MeSi(CF=CFCF_3) (a.6) (1.0 g, 3.0 mmol) was dissolved in dry THF (150 mL), PhLi (2.0 M, 7.5 mL, 15.0 mmol) was added slowly. After work up using (G.P2), the product was isolated as a brown liquid. Yield (0.7 g, 1.8 mmol, 60 %). Anal.Calcd for $C_{22}H_{18}F_4Si$: C, 68.37; H, 4.70 %. Found: C, 68.73; H, 4.90 %. ^{19}F{^{1}H} NMR (376 MHz, $CDCl_3$, 291 K) δ ppm: −61.13 (d, 3F, ^4J(CF_3 F_{gem}) = 9.6 Hz, CF_3), −79.91 (q, 1F, ^4J(F_{gem} CF_3) = 9.5 Hz, F_{gem}). ^{1}H NMR (400 MHz, $CDCl_3$, 291 K) δ ppm: 1.18 (s, 3H, CH_3), 7.39-7.41 (m, 15H, Ph). ^{13}C{^{1}H} NMR (100 MHz, $CDCl_3$, 298 K) δ ppm: −0.85 (s, Si-CH_3), 127.26 (s, Ph), 127.35 (s, Ph), 127.51 (s, Ph), 127.84 (s, Ph), 128.29 (s, Ph), 128.71 (s, Ph), 129.75 (s, Ph), 130.09 (s, Ph), C_1, C_2 and C_3 were not observed. ^{29}Si{^{1}H} NMR (79 MHz, $CDCl_3$, 298 K) δ ppm: −22.29 (d, ^2J(Si F_{gem}) = 36.3 Hz). ν_{max}/cm^{-1} 1270 (Si-C,w), 1305, 1440 (C-F, str), 1650 (C=C, w), 2990 (C-H, w), 3010, 3020 (C-H in Ph, m).

2-1-19. Reaction between Me_2Si(*E*-CF=CFCF_3)$_2$ (a.7) and ^{n}BuLi

Me_2Si(*E*-CF=CFCF_3)$_2$ (a.7) (1.2 g, 4.0 mmol) was dissolved in dry THF (150 mL), ^{n}BuLi (2.5 M, 6.4 mL, 16.0 mmol) was added slowly. After work up using (G.P2), the product was isolated as a brown liquid. Yield (0.4 g, 1.0 mol, 33 %); ratio *Z*:*E* [Me_2Si(*Z*-CF=C^nBuCF_3)$_2$ (30*Z*) 50 : Me_2Si(*E*-C^nBu=CFCF_3)$_2$ (30*E*) 50]. ^{19}F{^{1}H} NMR (376 MHz, $CDCl_3$, 291 K) δ ppm: Me_2Si(*Z*-CF=C^nBuCF_3)$_2$ −59.71 (d, 6F, ^4J(CF_3 F_{gem}) = 8.4 Hz, CF_3), −100.32 (q, 2F, ^4J(F_{gem} CF_3) = 8.5 Hz, F_{gem}) and

$Me_2Si(E\text{-}C^nBu{=}CFCF_3)_2$ −66.79 (d, 6F, $^3J(CF_3\ F_{trans})$ = 10.5 Hz, CF_3), −108.51 (q, 2F, $^3J(F_{trans}\ CF_3)$ = 10.0 Hz, F_{trans}). ^1H NMR (400 MHz, $CDCl_3$, 291 K) δ ppm: $Me_2Si(Z\text{-}CF{=}C^nBuCF_3)_2$ and $Me_2Si(E\text{-}C^nBu{=}CFCF_3)_2$ 0.11 (s, 6H, Si-CH_3), 0.14 (s, 6H, Si-CH_3), 0.18 (t, 6H, $^3J(CH_3\ CH_2)$ = 7.2 Hz, CH_3) 0.91 (t, 6H, $^3J(CH_3\ CH_2)$ = 7.4 Hz, CH_3), 1.25 (m, 8H, CH_2CH_2), 1.47 (m, 8H, CH_2CH_2), 2.15 (m, 4H, =C-CH_2), 2.20 (m, 4H, =C-CH_2). $^{13}C\{^1H\}$ NMR (100 MHz, $CDCl_3$, 298 K) δ ppm: $Me_2Si(Z\text{-}CF{=}C^nBuCF_3)_2$ and $Me_2Si(E\text{-}C^nBu{=}CFCF_3)_2$ −4.39 (s, Si-CH_3), −2.99 (s, Si-CH_3), 12.56 (s, =C-$\underline{C}H_2$), 12.78 (s, =C-$\underline{C}H_2$), 24.68 (s, =C-C_2H_2-$\underline{C}H_2$-CH_3), 24.96 (s, =C-C_2H_2-$\underline{C}H_2$-CH_3), 25.25 (s, =C-CH_2-$\underline{C}H_2$-C_2H_5), 25.40 (s, =C-CH_2-$\underline{C}H_2$-C_2H_5), $Me_2Si(Z\text{-}CF{=}C^nBuCF_3)_2$: 124.23 (q.d, $^1J(C_3\ 3F)$ = 266.8 Hz, $^3J(C_3\ F_{gem})$ = 26.0 Hz, C_3), 126.13 (m, C_2), 172.81 (d.q $^1J(C_1\ F_{gem})$ = 286.9 Hz, $^3J(C_1\ 3F)$ = 7.4 Hz, C_1); $Me_2Si(E\text{-}C^nBu{=}CFCF_3)_2$: 117.90 (q.d, $^1J(C_3\ 3F)$ = 271.9 Hz, $^2J(C_3\ F_{trans})$ = 50.0 Hz, C_3), 121.57 (m, C_1), 146.37 (d.q, $^1J(C_2\ F_{trans})$= 265.3 Hz, $^2J(C_2\ 3F)$ = 39.8 Hz, C_2). $^{29}Si\{^1H\}$ NMR (79 MHz, $CDCl_3$, 298 K) δ ppm: $Me_2Si(E\text{-}C^nBu{=}CFCF_3)_2$: −0.57 (m); $Me_2Si(Z\text{-}CF{=}C^nBuCF_3)_2$: −21.93 (m). ν_{max}/cm^{-1} 1250 (Si-C,w), 1380, 1460 (C-F, str), 1650 (C=C, w), 2970, 2930 (C-H, m).

2-1-20. Reaction between $Me_2Si(E\text{-}CF{=}CFCF_3)_2$ (a.7) and ^tBuLi

$Me_2Si(E\text{-}CF{=}CFCF_3)_2$ (a.7) (1.2 g, 4.0 mmol) was dissolved in dry THF (150 mL), ^tBuLi (1.7 M, 9.4 mL, 16.0 mmol) was added slowly. After work up using (G.P2), the product was isolated as a brown liquid. Yield (0.6 g, 1.5 mmol, 50 %). $^{19}F\{^1H\}$ NMR (376 MHz, $CDCl_3$, 291 K) δ ppm: −55.18 (d, 6F, $^4J(CF_3\ F_{gem})$ = 7.2 Hz, CF_3), −88.23 (q, 2F, $^4J(F_{gem}\ CF_3)$ = 7.5 Hz, F_{gem}). ^1H NMR (400 MHz, $CDCl_3$, 298 K) δ ppm: −0.13 (s, 6H, Si-CH_3), 1.07 (s, 18H, C-CH_3). $^{13}C\{^1H\}$ NMR (100 MHz, $CDCl_3$, 298 K) δ ppm: −3.74 (s, Si-CH_3), 12.69 (s, $\underline{C}$-CH_3), 28.89 (s, C-$\underline{C}H_3$), 124.48 (q.d, $^1J(C_3\ 3F)$ = 272.5 Hz, $^3J(C_3\ F_{gem})$ = 27.9 Hz, C_3), 131 (q.d, $^2J(C_2\ 3F)$ = 24.7 Hz, $^2J(C_2\ F_{gem})$ = 12.9 Hz, C_2), 174.78 (d.q $^1J(C_1\ F_{gem})$ = 293.8 Hz, $^3J(C_1\ 3F)$ = 9.3 Hz, C_1). $^{29}Si\{^1H\}$ NMR (79 MHz, $CDCl_3$, 298 K) δ ppm: 1.80 (m). ν_{max}/cm^{-1} 2960 (C-H, w), 1665 (C=C, w), 1365, 1465 (C-F, str), 1260 (Si-C, w).

2-1-21. Reaction between $Me_2Si(E\text{-}CF{=}CFCF_3)_2$ (a.7) and MeLi

$Me_2Si(E\text{-}CF{=}CFCF_3)_2$ (a.7) (1.5 g, 3.0 mmol) was dissolved in dry THF (150 mL), MeLi (1.6 M, 18.0 mL, 30 mmol) was added slowly. After work up using (G.P2), the product was isolated as yellow liquid. Yield (0.5 g, 1.5 mmol, 50 %); ratio *Z*:*E* [$Me_2Si(Z\text{-}CF{=}CMeCF_3)_2$ (32Z) 40 : $Me_2Si(E\text{-}CMe{=}CFCF_3)_2$ (32*E*) 60]. $^{19}F\{^1H\}$ NMR (376 MHz, $CDCl_3$, 291 K) δ ppm: $Me_2Si(Z\text{-}CF{=}CMeCF_3)_2$ −62.05 (d, 6F, $^4J(CF_3\ F_{gem})$ = 8.6 Hz, CF_3), −99.44 (q, 2F, $^4J(F_{gem}\ CF_3)$ = 8.5 Hz, F_{gem}) and $Me_2Si(E\text{-}CMe{=}CFCF_3)_2$ −66.75 (d, 2F, $^3J(CF_3\ F_{trans})$ = 10.3 Hz, CF_3), −107.19 (q, 1F, $^3J(F_{trans}\ CF_3)$ = 10.4 Hz, F_{trans}). ^1H NMR (400 MHz, $CDCl_3$, 291 K) δ ppm: $Me_2Si(Z\text{-}CF{=}CMeCF_3)_2$ and $Me_2Si(E\text{-}CMe{=}CFCF_3)_2$: 0.10 (s, 6H, Si-$CH_3$), 0.14 (s, 6H, Si-$CH_3$), 1.74 (s, 6H, =C-$CH_3$), 1.88 (s, 6H, =C-$CH_3$). $^{13}C\{^1H\}$ NMR (100 MHz, $CDCl_3$, 298 K) δ ppm: $Me_2Si(Z\text{-}CF{=}CMeCF_3)_2$ and $Me_2Si(E\text{-}CMe{=}CFCF_3)_2$: −0.38 (s, Si-$CH_3$), −0.44 (s, Si-$CH_3$), 0.37 (s, =C-$CH_3$), 0.71 (s, =C-$CH_3$); $Me_2Si(E\text{-}CMe{=}CFCF_3)_2$: 114.98 (q.d, $^1J(C_3\ 3F)$ = 22.3 Hz, $^2J(C_3\ F_{trans})$ = 49.4 Hz, C_3), 116.92 (m, C_1), 146.05 (d.q, $^1J(C_2\ F_{trans})$ = 228.5 Hz, $^2J(C_2\ 3F)$= 38.0 Hz C_2); and C_2, C_2,C_3 in $Me_2Si(Z\text{-}CF{=}CMeCF_3)_2$ were not observed. $^{29}Si\{^1H\}$ NMR (79 MHz, $CDCl_3$, 298 K) δ ppm: $Me_2Si(Z\text{-}CF{=}CMeCF_3)_2$ −22.76 (m); $Me_2Si(E\text{-}CMe{=}CFCF_3)_2$: −21.94 (m). $\nu_{max}/\ cm^{-1}$ 1265 (Si-C, w), 1345, 1455 (C-F, str), 1660 (C=C, w), 2980 (C-H, m).

2-1-22. Reaction between $^iPr_2Si(E\text{-}CF{=}CFCF_3)_2$ (a.8) and ^nBuLi

$^iPr_2Si(E\text{-}CF{=}CFCF_3)_2$ (a.8) (1.6 g, 4.0 mmol) was dissolved in dry THF (150 mL), ^nBuLi (2.5 M, 6.4 mL, 16.0 mmol). After work up using (G.P2), the product was isolated as a yellow liquid. Yield (0.4 g, 0.8 mmol, 40 %). $^{19}F\{^1H\}$ NMR (376 MHz, $CDCl_3$, 291 K) δ ppm: −61.98 (d, 6F, $^4J(CF_3\ F_{gem})$ = 8.6 Hz, CF_3), −101.26 (q, 2F, $^4J(F\ CF_3)$ = 8.4 Hz, F_{gem}). ^1H NMR (400 MHz, $CDCl_3$, 291 K) δ ppm: 0.89 (t, 6H, $^3J(CH_3\ CH_2)$ = 7.5 Hz, CH_3), 1.02 (d, 12H, $^3J(CH_3\ CH)$ = 7.5 Hz, Si-CH-C$\underline{H}_3$), 2.25 (m, 2H, Si-CH), 1.25 (m, 8H, CH_2-CH_2), 3.62 (t, 4H, $^3J(CH_2\ CH_2)$ = 6.2 Hz, =C-CH_2). $^{13}C\{^1H\}$ NMR (100 MHz, $CDCl_3$, 298 K) δ ppm: 11.46 (s, Si-CH), 13.58 (s, =C-CH_2), 14.32 (s, =C-C_3H_6-$\underline{C}H_3$), 17.15 (s, Si-CH-C$\underline{H}_3$), 22.56 (s, =C-$CH_2\underline{C}H_2$-C_2H_5), 26.86 (s, =C-$C_2H_4\underline{C}H_2$-CH_3), 124.63 (q.d, $^1J(C_3\ 3F)$ = 272.2 Hz, $^3J(C_3\ F_{gem})$ = 24.6 Hz, C_3), 128.58 (m, C_2), 167.90 (d.q, $^1J(C_1\ F_{gem})$ = 286.7 Hz, $^3J(C_1\ 3F)$ = 7.6 Hz, C_1). $^{29}Si\{^1H\}$ NMR (79 MHz, $CDCl_3$, 298 K) δ ppm: −3.27 (m). $\nu_{max}/\ cm^{-1}$ 1245 (Si-C, m), 1340, 1460 (C-F, str), 1600 (C=C, w), 2950, 2865 (C-H, m).

2-1-23. Reaction between $^{i}Pr_2Si$(*E*-CF=CFCF$_3$)$_2$ (a.8) and ^{t}BuLi

$^{i}Pr_2Si(CF=CFCF_3)_2$ (a.8) (1.6 g, 4.0 mmol) was dissolved in dry THF (150 mL), ^{t}BuLi (1.7 M, 9.4 mL, 16.0 mmol) was added slowly. After work up using (G.P2), the product was isolated as a yellow liquid. Yield (0.5 g, 1.0 mmol, 50 %). $^{19}F\{^1H\}$ NMR (376 MHz, $CDCl_3$, 291 K) δ ppm: −56.61 (d, 6F, $^4J(CF_3\ F_{gem})$ = 6.5 Hz, CF_3), −89.0 (q, 2F, $^4J(F_{gem}\ CF_3)$ = 5.9 Hz, F_{gem}). ^{1}H NMR (400 MHz, $CDCl_3$, 291 K) δ ppm: 0.98 (d, 12H, $^3J(CH_3\ CH)$ = 6.4 Hz, CH_3), 1.22 (s, 18H, C-CH_3), 1.87 (m, 2H, CH). $^{13}C\{^1H\}$ NMR (100 MHz, $CDCl_3$, 298 K) δ ppm: 12.01(s, C̲-CH_3) 13.30 (s, Si-CH), 16.20 (s, CH-C̲H_3), 28.1 (s,C-CH_3), 123.50 (q.d, $^1J(C_3\ 3F)$ = 272.6 Hz, $^3J(C_3\ F_{gem})$ = 26.5 Hz, C_3), 134.78 (m, C_2), 153.75 (d.q, $^1J(C_1\ F_{gem})$ = 282.5 Hz, $^3J(C_1\ 3F)$ = 5.54 Hz, C_1). $^{29}Si\{^1H\}$ NMR (79 MHz, $CDCl_3$, 298 K) δ ppm: −12.80 (m). ν_{max}/ cm^{-1} 1200 (Si-C, w), 1360, 1465 (C-F, str), 1665 (C=C, w), 2950, 2870 (C-H, m).

2-1-24. Reaction between $^{i}Pr_2Si$(*E*-CF=CFCF$_3$)$_2$ (a.8) and MeLi

$^{i}Pr_2Si$(*E*-CF=CFCF$_3$)$_2$ (a.8) (1.0 g, 2.0 mmol) was dissolved in dry THF (150 mL), MeLi (1.6 M, 12.5 mL, 20.0 mmol) was added slowly. After work up using (G.P2), the product was isolated as a yellow liquid. Yield (0.3 g, 0.8 mmol, 40 %). $^{19}F\{^1H\}$ NMR (376 MHz, $CDCl_3$, 291 K) δ ppm: −67.53 (m, 6F, CF_3), −139.77 (m, 2F, F_{gem}). ^{1}H NMR (400 MHz, $CDCl_3$, 291 K) δ ppm: 1.07 (d, 12H, $^3J(CH_3\ CH)$ = 5.0 Hz, CH_3), 1.81 (m, 2H, Si-CH), 2.42 (s, 6H, =C-CH_3). $^{13}C\{^1H\}$ NMR (100 MHz, $CDCl_3$, 298 K) δ ppm: 13.36 (s, =C-C̲H_3) 16.00 (s, Si-CH), 16.19 (s, CH-C̲H_3), 99.81 (q.d, $^1J(C_3\ 3F)$ = 246.6 Hz, $^3J(C_3\ F_{gem})$ = 36.8 Hz, C_3), 102.77 (m, C_2), 161.25 (d $^1J(C_1\ F_{gem})$ = 217.2 Hz, C_1). $^{29}Si\{^1H\}$ NMR (79 MHz, $CDCl_3$, 298 K) δ ppm: −13.16 (m). ν_{max}/cm^{-1} 1260 (Si-C, w), 1360, 1465 (C-F, str), 1660 (C=C, w), 2945, 2870 (C-H, m).

2-1-25. Reaction between Ph_2Si(*E*-CF=CFCF$_3$)$_2$ (a.9) and ^{n}BuLi

Ph_2Si(*E*-CF=CFCF$_3$)$_2$ (a.9) (1.0 g, 2.0 mmol) was dissolved in dry THF (150 mL), ^{n}BuLi (2.5 M, 3.2 mL, 8.0 mmol) was added slowly. After work up using (G.P2), the product was isolated as a yellow liquid. Yield (0.8 g, 1.5 mmol, 75 %); ratio *Z*:*E* [Ph_2Si(*Z*-CF=C^{n}BuCF$_3$)$_2$ (36*Z*) 77 : Ph_2Si(*E*-C^{n}Bu=CFCF$_3$)$_2$ (36*E*) 23]. Anal.Calcd for $C_{26}H_{28}F_8Si$: C, 59.98; H, 5.42 %. Found: C, 59.92; H, 5.22 %. $^{19}F\{^1H\}$ NMR (376 MHz, $CDCl_3$, 291 K) δ ppm: Ph_2Si(*Z*-CF=C^{n}BuCF$_3$)$_2$ −59.93 (d, 3F, $^4J(CF_3\ F_{gem})$ = 7.7 Hz, CF_3), −97.61 (q, 1F, $^4J(F_{gem}\ CF_3)$ = 8.0 Hz, F_{gem}) and Ph_2Si(*E*-C^{n}Bu=CFCF$_3$)$_2$ −67.02 (d, 3F, $^3J(CF_3\ F_{trans})$ = 10.5 Hz, CF_3), −105.09 (q, 1F, $^3J(F_{trans}\ CF_3)$ = 10.8 Hz, F_{trans}). ^{1}H NMR (400 MHz, $CDCl_3$, 291 K) δ ppm: Ph_2Si(*Z*-CF=C^{n}BuCF$_3$)$_2$ and Ph_2Si(*E*-C^{n}Bu=CFCF$_3$)$_2$: 0.79 (t, 6H, $^3J(CH_3\ CH_2)$ = 7.1 Hz, CH_3), 0.86 (t, 6H, $^3J(CH_3\ CH_2)$ = 7.3 Hz, CH_3), 1.30 (m, 8H, CH_2CH_2), 1.44 (m, 8H, CH_2CH_2), 2.42 (t, 4H, $^3J(CH_2\ CH_2)$ = 7.7 Hz, =C-CH_2), 2.49 (t, 4H, $^3J(CH_2\ CH_2)$ = 6.9 Hz, =C-CH_2), 7.32-7.63 (m, 20H, Ph). $^{13}C\{^1H\}$ NMR (100 MHz, $CDCl_3$, 298 K) δ ppm: Ph_2Si(*Z*-CF=C^{n}BuCF$_3$)$_2$ and Ph_2Si(*E*-C^{n}Bu=CFCF$_3$)$_2$: 11.28 (s, =C-C̲H_2), 11.82 (s, =C-C̲H_2), 12.51 (s,=C-C_3H_6-C̲H_3), 12.66 (s,=C-C_3H_6-C̲H_3), 24.76 (s, =C-C_4H_4-C̲H_2-CH_3), 25.46 (s, =C-C_4H_4-C̲H_2-CH_3), 28.37 (s, =C-CH_2-C̲H_2-C_2H_5), 29.82 (s, =C-CH_2-C̲H_2-C_2H_5), 126.63 (s, Ph), 126.69 (s, Ph), 126.78 (s, Ph), 126.86 (s, Ph), 128.55(s, Ph), 128.81 (s, Ph), 133.83 (s, Ph), 134.05 (s, Ph). Ph_2Si(*Z*-CF=C^{n}BuCF$_3$)$_2$: 123.80 (q.d, $^1J(C_3\ 3F)$ = 272.0 Hz, $^3J(C_3\ F_{gem})$ = 24.0 Hz, C_3), 146.73 (m, C_2), 169.14 (d.q $^1J(C_1\ F_{gem})$ = 286.0 Hz, $^3J(C_1\ 3F)$ = 6.8 Hz, C_1). Ph_2Si(*E*-C^{n}Bu=CFCF$_3$)$_2$: 117.79 (q.d, $^1J(C_3\ 3F)$ = 271.2 Hz, $^2J(C_3\ F_{trans})$ = 49.5 Hz, C_3), 141.57 (m, C_1), 178.53 (d, $^1J(C_2\ F_{trans})$ = 273 Hz, C_2). $^{29}Si\{^1H\}$ NMR (79 MHz, $CDCl_3$, 298 K) δ ppm: Ph_2Si(*Z*-CF=C^{n}BuCF$_3$)$_2$ −11.95 (m); Ph_2Si(*E*-C^{n}Bu=CFCF$_3$)$_2$: −9.25 (m). ν_{max}/ cm^{-1} 1230 (Si-C, w),1660 (C=C, w), 1360, 1430 (C-F, str), 2960 (C-H, m), 3050 (C-H in Ph, w).

2-1-26. Reaction between Ph_2Si(*E*-CF=CFCF$_3$)$_2$ (a.9) and PhLi

Ph_2Si(*E*-CF=CFCF$_3$)$_2$ (a.9) (1.0 g, 2.0 mmol) was dissolved in dry THF (150 mL), PhLi (2.0 M, 15.0 mL, 20.0 mmol) was added slowly. The product was isolated as a yellow liquid after work up and purified by column chromatography on silica with a chloroform eluent. Yield (0.3 g, 0.5 mmol, 25 %). Anal.Calcd for $C_{30}H_{20}F_8Si$: C, 64.27; H, 3.60 %. Found: C, 64.45; H, 3.08 %. $^{19}F\{^1H\}$ NMR (376 MHz, $CDCl_3$, 291 K) δ ppm: −57.84 (d, 6F, $^4J(CF_3\ F_{gem})$ = 8.4 Hz, CF_3), −90.35 (q, 2F, $^4J(F_{gem}\ CF_3)$ = 8.8 Hz, F_{gem}). ^{1}H NMR (400 MHz, $CDCl_3$, 291 K) δ ppm: 7.53-7.41 (m, 20H, Ph). $^{13}C\{^1H\}$ NMR (100 MHz, $CDCl_3$, 298 K) δ ppm: 123.33 (s, Ph), 124.34 (s, Ph), 126.11 (s, Ph), 12.20 (s, Ph), 127.15 (s, Ph), 127.70 (s, Ph), 129.24 (s, Ph), 129.63 (s, Ph), C_1, C_2, and C_3 were not observed. $^{29}Si\{^1H\}$ NMR (79 MHz, $CDCl_3$, 298 K) δ ppm: −21.80 (m). ν_{max}/cm^{-1} 3150 (C-H in Ph, w) 2985 (C-H, m), 1670 (C=C, w), 1345, 1425 (C-F, str), 1270 (Si-C, w).

3. DFT calculations

3.1 Calculated Mulliken Charges

Calculated Mulliken charges was optimised using hybrid Density Functional Theory (DFT) at the B3LYP/6-31G (d,p) level using the GAMESS software and Avogadro to present the geometric structure

3.1.1 $(Me)_2Si(\textit{E}\text{-}CF{=}CFCF_3)_2$ (a.5)

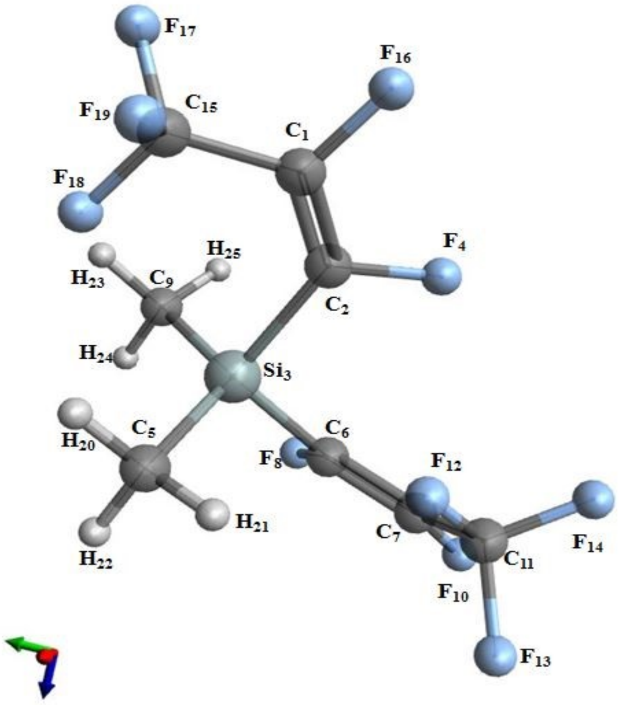

Figure 3 Avogadro representation of the geometric structure $(Me)_2Si(\textit{E}\text{-}CF{=}CFCF_3)_2$ at lowest energy state

Table 1 Total Mulliken and Lowdin Atomic Populations

Atom	Mull.POP.	Charge	Low.POP.	Charge
C 1	5.792422	0.207578	5.937545	0.062455
C 2	5.853106	0.146894	5.996687	0.003313
Si 3	13.547697	0.452303	13.554013	0.445987
F 4	9.275586	-0.275586	9.128182	-0.128182
C 5	6.505396	-0.505396	6.486134	-0.486134
C 6	5.842232	0.157768	5.991868	0.008132
C 7	5.776375	0.223625	5.929703	0.070297
F 8	9.280584	-0.280584	9.131134	-0.131134
C 9	6.492158	-0.492158	6.480313	-0.480313
F 10	9.265644	-0.265644	9.107236	-0.107236
C 11	5.206878	0.793122	5.643280	0.356720
F 12	9.253044	-0.253044	9.111325	-0.111325
F 13	9.254959	-0.254959	9.119764	-0.119764
F 14	9.247121	-0.247121	9.111663	-0.111663
C 15	5.210271	0.789729	5.640200	0.359800
F 16	9.264841	-0.264841	9.108460	-0.108460
F 17	9.249437	-0.249437	9.114282	-0.114282
F 18	9.256707	-0.256707	9.117453	-0.117453
F 19	9.249996	-0.249996	9.118715	-0.118715
H 20	0.862987	0.137013	0.862371	0.137629
H 21	0.859940	0.140060	0.862356	0.137644
H 22	0.868800	0.131200	0.863260	0.136740
H 23	0.865172	0.134828	0.862393	0.137607
H 24	0.861030	0.138970	0.862018	0.137982
H 25	0.857620	0.142380	0.859644	0.140356

Table 2 Bond order and valence analysis, (bond order threshold=0.050)

Atom Pair	Distance	Order	Atom Pair	Distance	Order	Atom Pair	Distance	Order
1 2	1.339	1.634	1 4	2.269	0.085	1 15	1.497	0.959
1 16	1.344	0.986	2 3	1.925	0.891	2 4	1.362	0.893
2 16	2.341	0.060	3 4	2.756	0.051	3 5	1.882	0.992
3 6	1.911	0.905	3 8	2.732	0.061	3 9	1.877	1.008
3 12	3.035	0.062	3 18	2.990	0.064	5 20	1.092	0.943
5 21	1.092	0.943	5 22	1.096	0.945	6 7	1.338	1.637
6 8	1.364	0.881	6 10	2.337	0.062	7 8	2.277	0.087
7 10	1.344	0.986	7 11	1.500	0.954	9 23	1.093	0.944
9 24	1.094	0.944	9 25	1.094	0.944	11 12	1.354	0.958
11 13	1.347	1.016	11 14	1.343	1.026	15 17	1.344	1.024

Table 3 Bond order and valence analysis, (bond order threshold=0.050)

		Coordinates (Bohr)		
**Atom**	**Atomic Charge**	**X**	**Y**	**Z**
C	6	-1.08472	-6.59514	-0.18943
C	6	-2.43374	-7.10155	1.890595
Si	14	-2.82939	-5.37283	5.06696
F	9	-3.82647	-9.26026	1.74896
C	6	0.242433	-4.78072	6.756848
C	6	-4.88194	-7.6391	6.989189
C	6	-4.62809	-10.0669	7.648893
F	9	-7.04609	-6.51266	7.819535
C	6	-4.76073	-2.4328	4.612198
F	9	-6.40251	-11.2523	9.024972
C	6	-2.4865	-11.8179	7.027626
F	9	-0.62038	-10.6208	5.751608
F	9	-1.47514	-12.7777	9.157442
F	9	-3.27057	-13.7717	5.610426
C	6	0.5703	-4.34622	-0.64625
F	9	-1.11335	-8.1458	-2.19968
F	9	-0.06497	-3.17398	-2.80711
F	9	0.344081	-2.65206	1.264491
F	9	3.024317	-4.98859	-0.81857
H	1	1.476159	-3.54505	5.657084
H	1	1.239755	-6.5497	7.124229
H	1	-0.14247	-3.86672	8.574159
H	1	-3.69296	-1.08667	3.46502
H	1	-5.20036	-1.55405	6.43155
H	1	-6.54301	-2.84117	3.649288

3.1.2 $(Ph)_2Si(E\text{-}CF{=}CFCF_3)_2$ (a.9)

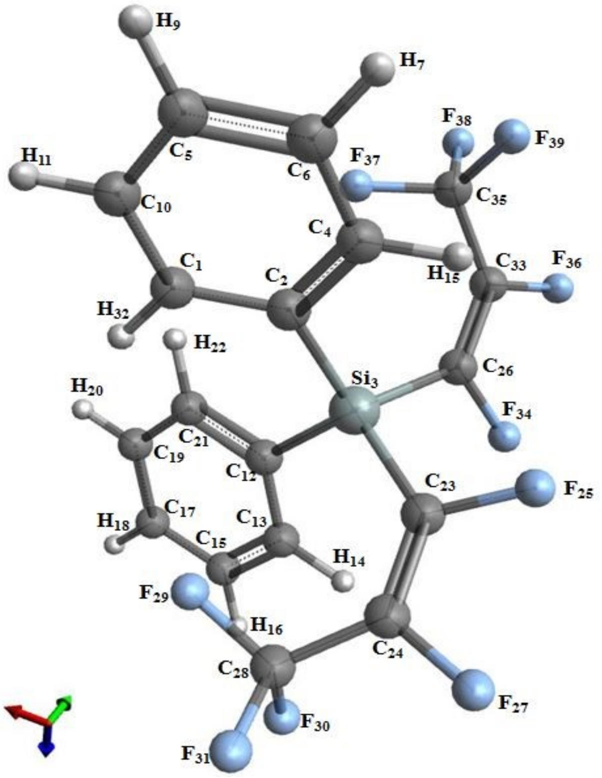

Figure 4 Avogadro representation of the geometric structure $(Ph)_2Si(CF{=}CFCF_3)_2$ at lowest energy state

Table 4 Total mulliken and lowdin atomic populations

Atoms	Mull.POP.	Charge	Low.POP.	Charge
C 1	6.142560	-0.142560	6.091330	-0.091330
C 2	6.216418	-0.216418	6.181938	-0.181938
Si 3	13.231213	0.768787	13.477300	0.522700
C 4	6.148941	-0.148941	6.089488	-0.089488
H 5	0.844632	0.155368	0.879434	0.120566
C 6	6.146228	-0.146228	6.111781	-0.111781
H 7	0.838527	0.161473	0.879590	0.120410
C 8	6.138140	-0.138140	6.089432	-0.089432
H 9	0.839509	0.160491	0.881086	0.118914
C 10	6.147856	-0.147856	6.112622	-0.112622
H 11	0.841153	0.158847	0.882273	0.117727
C 12	6.213223	-0.213223	6.178071	-0.178071
C 13	6.139245	-0.139245	6.086659	-0.086659
H 14	0.848762	0.151238	0.881655	0.118345
C 15	6.147688	-0.147688	6.110692	-0.110692
H 16	0.838270	0.161730	0.880429	0.119571
C 17	6.137896	-0.137896	6.090585	-0.090585
H 18	0.839160	0.160840	0.879810	0.120190
C 19	6.148528	-0.148528	6.111928	-0.111928
H 20	0.840626	0.159374	0.882927	0.117073
C 21	6.151413	-0.151413	6.093585	-0.093585
H 22	0.839001	0.160999	0.877163	0.122837
C 23	5.843937	0.156063	5.956401	0.043599
C 24	5.746694	0.253306	5.896504	0.103496
F 25	9.365694	-0.365694	9.178178	-0.178178
C 26	5.842656	0.157344	5.959056	0.040944
F 27	9.359016	-0.359016	9.159134	-0.159134
C 28	4.872348	1.127652	5.538094	0.461906
F 29	9.352952	-0.352952	9.146686	-0.146686
F 30	9.352668	-0.352668	9.159144	-0.159144
F 31	9.348166	-0.348166	9.149010	-0.149010
H 32	0.840764	0.159236	0.876057	0.123943

C 33	5.740315	0.259685	5.896184	0.103816
F 34	9.366531	-0.366531	9.179956	-0.179956
C 35	4.877176	1.122824	5.539345	0.460655
F 36	9.358906	-0.358906	9.158966	-0.158966
F 37	9.352082	-0.352082	9.148370	-0.148370
F 38	9.349168	-0.349168	9.150301	-0.150301
F 39	9.351939	-0.351939	9.158836	-0.158836

Table 5 Bond order and valence analysis, (bond order threshold=0.050)

Atom Pair	Distance	Order	Atom Pair	Distance	Order	Atom Pair	Distance	Order
1 2	1.395	1.432	1 6	2.769	0.087	1 10	1.386	1.444
1 32	1.075	0.953	2 3	1.874	0.961	2 4	1.398	1.406
2 8	2.795	0.090	3 12	1.876	0.964	3 23	1.925	0.883
3 26	1.921	0.888	3 34	2.693	0.057	4 5	1.077	0.954
4 6	1.383	1.467	4 10	2.766	0.089	6 7	1.075	0.959
6 8	1.386	1.437	8 9	1.076	0.959	8 10	1.384	1.452
10 11	1.076	0.960	12 13	1.396	1.415	12 17	2.796	0.090
12 21	1.397	1.419	13 14	1.075	0.954	13 15	1.384	1.458
13 19	2.767	0.088	15 16	1.075	0.959	15 17	1.385	1.443
15 21	2.767	0.088	17 18	1.076	0.959	17 19	1.385	1.446
19 20	1.075	0.959	19 21	1.385	1.453	21 22	1.076	0.953
23 24	1.317	1.780	23 25	1.340	0.800	24 27	1.323	0.871
24 28	1.497	1.001	26 33	1.317	1.775	26 34	1.341	0.796
28 29	1.320	0.888	28 30	1.319	0.915	28 31	1.316	0.929
33 35	1.499	1.001	33 36	1.324	0.869	35 37	1.320	0.894
35 38	1.316	0.929	35 39	1.319	0.916			

Table 6 Bond order and valence analysis, (bond order threshold=0.050)

		Coordinates (Bohr)		
Atom	Atomic Charge	X	Y	Z
C	6	1.635087	-8.65531	2.725867
C	6	-0.62486	-7.31582	2.934171
Si	14	-2.10928	-6.65697	6.081856
C	6	-1.88067	-6.63784	0.711919
H	1	-3.63663	-5.61362	0.797839
C	6	-0.89894	-7.24915	-1.63121
H	1	-1.89024	-6.70191	-3.3187
C	6	1.360707	-8.56432	-1.79829
H	1	2.127673	-9.04028	-3.61956
C	6	2.623894	-9.26993	0.380043
H	1	4.372106	-10.2995	0.258684
C	6	0.045194	-6.7761	8.893501
C	6	-0.80377	-7.60166	11.25075
H	1	-2.68928	-8.32936	11.46415
C	6	0.751927	-7.5132	13.35132
H	1	0.062006	-8.16621	15.14766
C	6	3.190224	-6.58602	13.13591
H	1	4.40116	-6.5187	14.76721
C	6	4.06391	-5.73978	10.81908
H	1	5.952229	-5.00893	10.64404
C	6	2.502593	-5.82856	8.720869
H	1	3.214229	-5.14672	6.943026
C	6	-4.98562	-8.86942	6.334551

C	6	-5.2589	-11.2645	6.952272
F	9	-7.10977	-7.74342	5.540268
C	6	-3.80564	-3.45302	6.282731
F	9	-7.49983	-12.3722	6.885881
C	6	-3.26089	-13.1114	7.724156
F	9	-0.97253	-12.204	7.321036
F	9	-3.43632	-13.703	10.13967
F	9	-3.49562	-15.2138	6.417907
H	1	2.636548	-9.23619	4.395148
C	6	-3.43232	-1.18455	5.329848
F	9	-5.74403	-3.59912	7.909404
C	6	-1.342	-0.30521	3.631008
F	9	-4.97509	0.705904	5.880063
F	9	0.602279	-1.86711	3.682402
F	9	-0.54317	1.930084	4.371364
F	9	-2.08879	-0.09174	1.261884

3.1.3 (Ph)Si(*E*-CF=CFCF3)$_3$ (a.10)

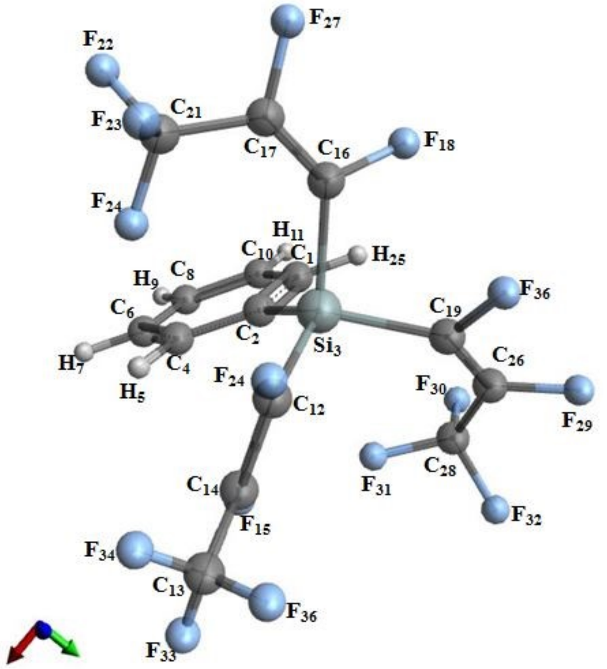

Figure 5 Avogadro representation of the geometric structure (Ph)Si(CF=CFCF$_3$)$_3$ at lowest energy state

Table 7 Total Mulliken and Lowdin Atomic Populations

Atom	Mull.POP.	Charge	Low.POP.	Charge
C 1	6.129935	-0.129935	6.080355	-0.080355
C 2	6.195701	-0.195701	6.182192	-0.182192
Si 3	13.292030	0.707970	13.469776	0.530224
C 4	6.151800	-0.151800	6.085093	-0.085093
H 5	0.842250	0.157750	0.879847	0.120153
C 6	6.145844	-0.145844	6.111514	-0.111514
H 7	0.834315	0.165685	0.877322	0.122678
C 8	6.133672	-0.133672	6.081233	-0.081233
H 9	0.833875	0.166125	0.878462	0.121538
C 10	6.148626	-0.148626	6.107627	-0.107627

H 11	0.834503	0.165497	0.881264	0.118736
C 12	5.842197	0.157803	5.961179	0.038821
C 13	4.833097	1.166903	5.538683	0.461317
C 14	5.774678	0.225322	5.892564	0.107436
F 15	9.368802	-0.368802	9.163523	-0.163523
C 16	5.845001	0.154999	5.967077	0.032923
C 17	5.724840	0.275160	5.878290	0.121710
F 18	9.364335	-0.364335	9.175569	-0.175569
C 19	5.847159	0.152841	5.973540	0.026460
F 20	9.351913	-0.351913	9.150483	-0.150483
C 21	4.868291	1.131709	5.538938	0.461062
C 22	9.360110	-0.360110	9.148117	-0.148117
F 23	9.341656	-0.341656	9.145425	-0.145425
F 24	9.347557	-0.347557	9.148009	-0.148009
H 25	0.839303	0.160697	0.875435	0.124565
C 26	5.712298	0.287702	5.876105	0.123895
F 27	9.367008	-0.367008	9.182639	-0.182639
C 28	4.877734	1.122266	5.544391	0.455609
F 29	9.351481	-0.351481	9.150176	-0.150176
F 30	9.342343	-0.342343	9.139296	-0.139296
F 31	9.347554	-0.347554	9.148728	-0.148728
F 32	9.350397	-0.350397	9.154970	-0.154970
F 33	9.354101	-0.354101	9.156832	-0.156832
F 34	9.338172	-0.338172	9.137778	-0.137778
F 35	9.357784	-0.357784	9.164957	-0.164957
F 36	9.349639	-0.349639	9.152612	-0.152612

Table 8 Bond order and valence analysis, (bond order threshold=0.050)

Atom Pair	Distance	Order	Atom Pair	Distance	Order	Atom Pair	Distance	Order
1 2	1.394	1.421	1 6	2.772	0.088	1 10	1.384	1.454
1 25	1.074	0.954	2 3	1.862	0.984	2 4	1.397	1.407
2 8	2.789	0.088	3 12	1.908	0.889	3 16	1.910	0.917
3 19	1.904	0.928	3 27	2.625	0.063	4 5	1.078	0.956
4 6	1.383	1.466	4 10	2.766	0.088	6 7	1.075	0.957
6 8	1.386	1.441	8 9	1.076	0.957	8 10	1.385	1.446
10 11	1.075	0.958	12 14	1.313	1.763	12 35	1.331	0.829
13 14	1.502	0.971	13 33	1.319	0.924	13 34	1.310	0.954
13 36	1.317	0.928	14 15	1.333	0.816	16 17	1.315	1.780
16 18	1.338	0.804	17 20	1.319	0.883	17 21	1.497	1.001
19 26	1.316	1.759	19 27	1.345	0.786	21 22	1.323	0.871
21 23	1.313	0.930	21 24	1.316	0.927	26 28	1.500	0.996
26 29	1.320	0.878	28 30	1.315	0.916	28 31	1.316	0.929
28 32	1.319	0.919						

Table 9 Bond order and valence analysis, (bond order threshold=0.050)

		Coordinates (Bohr)		
**Atom**	**Atomic Charge**	**X**	**Y**	**Z**
C	6	2.089678	-7.75113	2.546727
C	6	-0.50182	-7.40181	2.864409
Si	14	-1.96426	-6.59803	5.96218
C	6	-2.08099	-7.72108	0.773635
H	1	-4.09538	-7.48059	0.955351
C	6	-1.10065	-8.35166	-1.56543
H	1	-2.34211	-8.58317	-3.15698
C	6	1.480544	-8.69076	-1.84577
H	1	2.247319	-9.18845	-3.66102
C	6	3.0726	-8.39318	0.20906

H	1	5.075558	-8.65803	-0.00467
C	6	0.112269	-7.1662	8.85433
C	6	4.017879	-6.09365	11.5107
C	6	2.218841	-5.94081	9.31994
F	9	3.019707	-4.25364	7.630525
C	6	-5.01915	-8.46782	6.407626
C	6	-5.43466	-10.8795	6.841507
F	9	-7.08704	-7.03443	6.149849
C	6	-3.19662	-3.26509	6.526641
F	9	-7.74389	-11.7894	7.068407
C	6	-3.50624	-12.9355	7.090025
F	9	-1.2109	-12.0795	6.593519
F	9	-3.49839	-13.906	9.374477
F	9	-4.00206	-14.769	5.485005
H	1	3.36084	-7.51718	4.110343
C	6	-3.13604	-1.03801	5.422002
F	9	-4.50031	-3.33461	8.70712
C	6	-1.80129	-0.25113	3.047656
F	9	-4.35965	0.896014	6.413938
F	9	-0.09919	-1.91945	2.345471
F	9	-0.65247	1.922059	3.419289
F	9	-3.41126	0.050701	1.169098
F	9	6.276101	-6.77265	10.70233
F	9	3.275349	-7.72206	13.2198
F	9	-0.7414	-8.82812	10.53719
F	9	4.243909	-3.8598	12.58444

3.2 Electronic structure and DFT reaction energetics for substitution reaction with nuclephilic sources

Reaction energetics (ΔE) were calculated for substitution reaction in which *Z*-isomer (1) and *E*-isomer (2) are formed:

(1) R$_{4\text{-}n}$Si(*E*-CF=CFCF$_3$)$_n$ + R'Li → R$_3$Si(*Z*-CF=CR'CF$_3$)$_n$ + LiF

(2) R$_{4\text{-}n}$Si(*E*-CF=CFCF$_3$)$_n$ + R'Li → R$_3$Si(*E*-CR'=CFCF$_3$)$_n$ + LiF

ΔE was computed according to:

(3) $\Delta E = E^{DFT}(\text{products}) - E^{DFT}(\text{reactants})$

Table 10 Electronic structure: HOMO, LUMO, electrostatic potential map (EP) colour coded on the charge density (isovalue 0.01) showing electron rich/deficient (red/blue) regions for compounds (**a.1**, **a.2**, **a.4**, **a.5**, **a.6**, **a.7**, **a.8**, and **a.9**) and DFT energetics ΔE [kcal/mol] computed for substitution reaction (1) and (2).

Electronic structure of compound			Nucleophilic sources	Product	ΔE [kcal/mol]
HOMO	LUMO	EP			
(Et)$_3$Si(*E*-CF=CFCF$_3$) (**a.1**)					
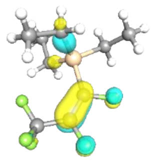	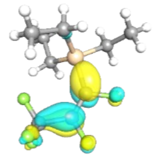	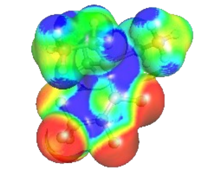	^{n}BuLi	(Et)$_3$Si(*Z*-CF=C^{n}BuCF$_3$) (**12*Z***)	-76.35
				(Et)$_3$Si(*E*-C^{n}Bu=CFCF$_3$) (**12*E***)	-70.27
			^{t}BuLi	(Et)$_3$Si(*Z*-CF=C^{t}BuCF$_3$) (**13*Z***)	-72.86
				(Et)$_3$Si(*E*-C^{t}Bu=CFCF$_3$)	-59.34
			MeLi	(Et)$_3$Si(Z-CF=CMeCF$_3$) (**14*Z***)	-73.55
				(Et)$_3$Si(*E*-CMe=CFCF$_3$) (**14*E***)	-67.31
			PhLi	(Et)$_3$Si(*Z*-CF=CPhCF$_3$) (**15*Z***)	-69.99
				(Et)$_3$Si(*E*-CPh=CFCF$_3$) (**15*E***)	-66.70
(^{n}Bu)$_3$Si(*E*-CF=CFCF$_3$) (**a.2**)					
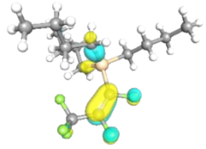	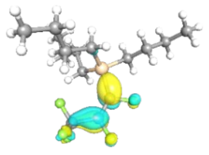	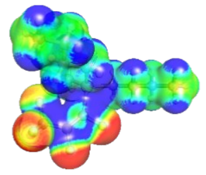	^{n}BuLi	(^{n}Bu)$_3$Si(*Z*-CF=C^{n}BuCF$_3$) (**16*Z***)	-76.26
				(^{n}Bu)$_3$Si(*E*-C^{n}Bu=CFCF$_3$) (**16*E***)	-70.13
			^{t}BuLi	(^{n}Bu)$_3$Si(*Z*-CF=C^{t}BuCF$_3$) (**17*Z***)	-72.80
				(^{n}Bu)$_3$Si(*E*-C^{t}Bu=CFCF$_3$)	-59.06
			MeLi	(^{n}Bu)$_3$Si(Z-CF=CMeCF$_3$) (**18*Z***)	-72.39
				(^{n}Bu)$_3$Si(*E*-CMe=CFCF$_3$) (**18*E***)	-67.19
			PhLi	(^{n}Bu)$_3$Si(*Z*-CF=CPhCF$_3$) (**19*Z***)	-69.99
				(^{n}Bu)$_3$Si(*E*-CPh=CFCF$_3$) (**19*E***)	-66.55
^{n}Bu(Me)$_2$Si(*E*-CF=CFCF$_3$) (**a.4**)					
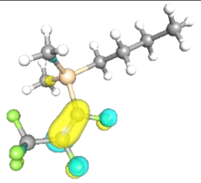	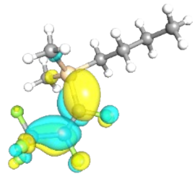	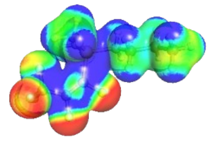	^{n}BuLi	^{n}Bu(Me)$_2$Si(*Z*-CF=C^{n}BuCF$_3$) (**20*Z***)	-75.51
				^{n}Bu(Me)$_2$Si(*E*-C^{n}Bu=CFCF$_3$) (**20*E***)	-69.84
			^{t}BuLi	^{n}Bu(Me)$_2$Si(*Z*-CF=C^{t}BuCF$_3$) (**21*Z***)	-72.35
				^{n}Bu(Me)$_2$Si(*E*-C^{t}Bu=CFCF$_3$)	-62.45
			MeLi	^{n}Bu(Me)$_2$Si(*Z*-CF=CMeCF$_3$) (**22*Z***)	-72.80
				^{n}Bu(Me)$_2$Si(*E*-CMe=CFCF$_3$) (**22*E***)	-68.13
			PhLi	^{n}Bu(Me)$_2$Si(*Z*-CF=CPhCF$_3$)	-69.15
				^{n}Bu(Me)$_2$Si(*E*-CPh=CFCF$_3$)	-67.07
(Me)$_2$PhSi(*E*-CF=CFCF$_3$) (**a.5**)					
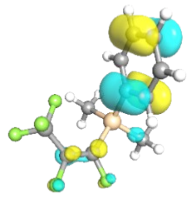	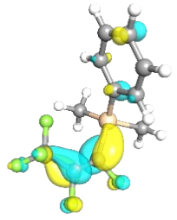	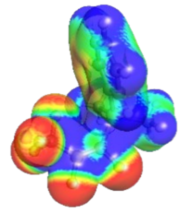	^{n}BuLi	Me$_2$PhSi(*Z*-CF=C^{n}BuCF$_3$) (**23*Z***)	-76.68
				Me$_2$PhSi(*E*-C^{n}Bu=CFCF$_3$) (**23*E***)	-71.58
			^{t}BuLi	(Me)$_2$PhSi(*Z*-CF=C^{t}BuCF$_3$) (**24*Z***)	-72.37
				(Me)$_2$PhSi(*E*-C^{t}Bu=CFCF$_3$) (**24*E***)	-63.14
			MeLi	(Me)$_2$PhSi(Z-CF=CMeCF$_3$) (**25*Z***)	-72.75
				(Me)$_2$PhSi(*E*-CMe=CFCF$_3$) (**25*E***)	-69.06
			PhLi	(Me)$_2$PhSi(Z-CF=CPhCF$_3$)	-69.10
				(Me)$_2$PhSi(*E*-CPh=CFCF$_3$)	-66.96
(Ph)$_2$MeSi(*E*-CF=CFCF$_3$) (**a.6**)					
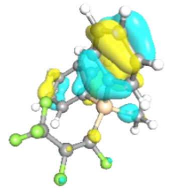	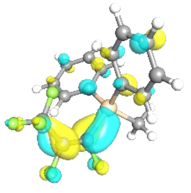	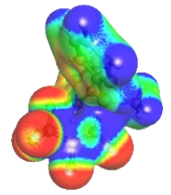	^{n}BuLi	(Ph)$_2$MeSi(*Z*-CF=C^{n}BuCF$_3$) (**26*Z***)	-76.63
				(Ph)$_2$MeSi(*E*-C^{n}Bu=CFCF$_3$) (**26*E***)	-72.30
			^{t}BuLi	(Ph)$_2$MeSi(*Z*-CF=C^{t}BuCF$_3$) (**27*Z***)	-72.24
				(Ph)$_2$MeSi(*E*-C^{t}Bu=CFCF$_3$)	-64.10
			MeLi	(Ph)$_2$MeSi(*Z*-CF=CMeCF$_3$) (**28*Z***)	-72.70
				(Ph)$_2$MeSi(*E*-CMe=CFCF$_3$) (**28*E***)	-69.89
			PhLi	(Ph)$_2$MeSi(*Z*-CF=CPhCF$_3$) (**29*Z***)	-68.93
				(Ph)$_2$MeSi(*E*-CPh=CFCF$_3$)	-66.86
(Me)$_2$Si(*E*-CF=CFCF$_3$)$_2$ (**a.7**)					
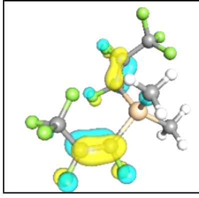	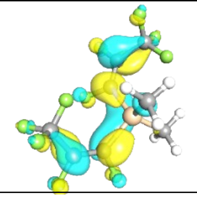	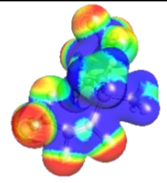	^{n}BuLi	(Me)$_2$Si(*Z*-CF=C^{n}BuCF$_3$)$_2$ (**30*Z***)	-77.56
				(Me)$_2$Si(*E*-C^{n}Bu=CFCF$_3$)$_2$ (**30*E***)	-73.24
			^{t}BuLi	(Me)$_2$Si(*Z*-CF=C^{t}BuCF$_3$)$_2$ (**31*Z***)	-81.69
				(Me)$_2$Si(*E*-C^{t}Bu=CFCF$_3$)$_2$	-64.25
			MeLi	(Me)$_2$Si(*Z*-CF=CMeCF$_3$)$_2$ (**32*Z***)	-73.58
				(Me)$_2$Si(*E*-CMe=CFCF$_3$)$_2$ (**32*E***)	-70.29
			PhLi	(Me)$_2$Si(*Z*-CF=CPhCF$_3$)$_2$	-69.82

				(Me)$_2$Si(*E*-CPh=CFCF$_3$)$_2$	-68.58
(^{i}Pr)$_2$Si(*E*-CF=CFCF$_3$)$_2$ (**a.8**)					
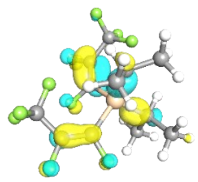	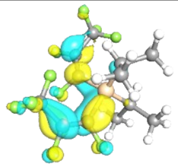	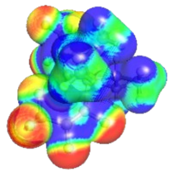	^{n}BuLi	(^{i}Pr)$_2$Si(*Z*-CF=C^{n}BuCF$_3$)$_2$ (**33*Z***)	-77.26
				(^{i}Pr)$_2$Si(*E*-C^{n}Bu=CFCF$_3$)$_2$	-69.34
			^{t}BuLi	(^{i}Pr)$_2$Si(*Z*-CF=C^{t}BuCF$_3$)$_2$ (**34*Z***)	-73.08
				(^{i}Pr)$_2$Si(*E*-C^{t}Bu=CFCF$_3$)$_2$	-57.94
			MeLi	(^{i}Pr)$_2$Si(*Z*-CF=CMeCF$_3$)$_2$ (**35*Z***)	-73.38
				(^{i}Pr)$_2$Si(*E*-CMe=CF CF$_3$)$_2$	-67.84
			PhLi	(^{i}Pr)$_2$Si(*Z*-CF=CPhCF$_3$)$_2$	-69.72
				(^{i}Pr)$_2$Si(*E*-CPh=CF CF$_3$)$_2$	-65.62
(Ph)$_2$Si(*E*-CF=CFCF$_3$)$_2$ (**a.9**)					
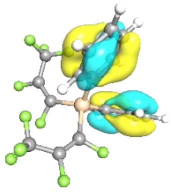	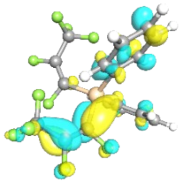	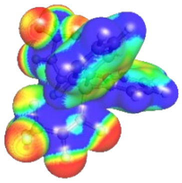	^{n}BuLi	(Ph)$_2$Si(*Z*-CF=C^{n}BuCF$_3$)$_2$ (**36*Z***)	-77.081
				(Ph)$_2$Si(*E*-C^{n}Bu=CFCF$_3$)$_2$ (**36*E***)	-72.876
			^{t}BuLi	(Ph)$_2$Si(*Z*-CF=C^{t}BuCF$_3$)$_2$	-72.736
				(Ph)$_2$Si(*E*-C^{t}Bu=CFCF$_3$)$_2$	-63.933
			MeLi	(Ph)$_2$Si(*Z*-CF=CMeCF$_3$)$_2$	-73.245
				(Ph)$_2$Si(*E*-CMe=CFCF$_3$)$_2$	-70.407
			PhLi	(Ph)$_2$Si(*Z*-CF=CPhCF$_3$)$_2$ (**37*Z***)	-69.534
				(Ph)$_2$Si(*E*-CPh=CFCF$_3$)$_2$	-67.947

4. Crystallographic data

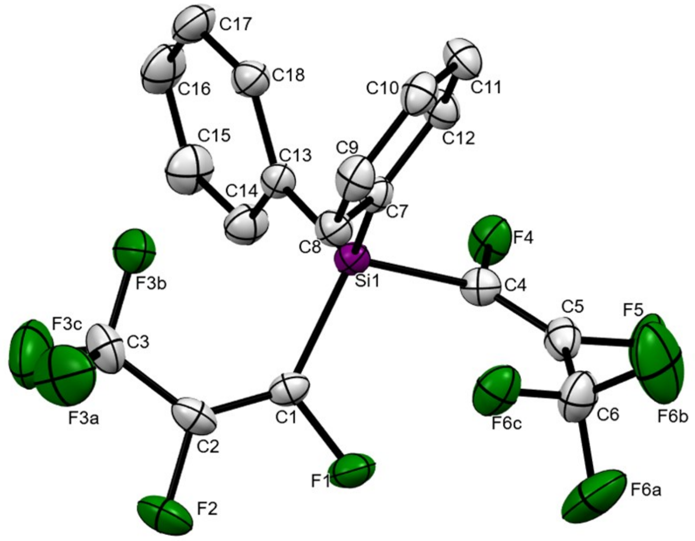

Figure 6 ORTEP representation of the structure of $(Ph)_2Si(CF{=}CFCF_3)_2$ (**a.9**), (hydrogen atoms omitted for clarity), thermal ellipsoids are shown at 50%

Table 11 Summary of crystallographic data of $(Ph)_2Si(CF{=}CFCF_3)_2$

Chemical formula	$C_{18}H_{10}F_{10}Si$
M_r	444.35
Crystal system, space group	Triclinic, *P*1
Temperature (K)	150
a, *b*, *c* (Å)	9.4952 (8), 10.2127 (10), 10.9000 (9)
a, b, g (°)	81.603 (8), 88.316 (7), 63.297 (9)
V ($Å^3$)	933.36 (16)
Z	2
Radiation type	Mo *K*a
m (mm^{-1})	0.22
Crystal size (mm)	0.45 × 0.25 × 0.12
Diffractometer	SuperNova, Single source at offset, Eos
Absorption correction	Multi-scan *CrysAlis PRO* 1.171.38.41 (Rigaku Oxford Diffraction, 2015) Empirical absorption correction using spherical harmonics, implemented in SCALE3 ABSPACK scaling algorithm.
T_{min}, T_{max}	0.580, 1.000
No. of measured, independent and observed [I ³ 2u(I)] reflections	7301, 3974, 3113
R_{int}	0.029
(sin q/l)$_{max}$ ($Å^{-1}$)	0.617
$R[F^2 > 2s(F^2)]$, $wR(F^2)$, S	0.048, 0.100, 1.05
No. of reflections	3668
No. of parameters	262
H-atom treatment	H-atom parameters constrained
Dρ$_{max}$, Dρ$_{min}$ (e $Å^{-3}$)	0.51, -0.38

Table 12 Geometric parameters (Å& º) of $(Ph)_2Si(CF{=}CFCF_3)_2$

Atoms	Bond length Å	Atoms	Bond length Å
Si1—C4	1.906 (2)	C11—C12	1.379 (3)
Si1—C1	1.895 (2)	C11—C10	1.385 (3)
Si1—C13	1.853 (2)	C12—C7	1.397 (3)
Si1—C7	1.850 (2)	C15—C14	1.380 (3)
F2—C2	1.342 (3)	C8—C9	1.383 (3)
F1—C1	1.362 (2)	C8—C7	1.393 (3)
F4—C4	1.365 (3)	C9—C10	1.377 (4)
C4—C5	1.319 (3)	C1—C2	1.323 (3)
F6c—C6	1.329 (3)	F6b—C6	1.314 (3)
F3b—C3	1.326 (3)	C13—C18	1.394 (3)
F5—C5	1.349 (3)	C13—C14	1.396 (4)
C5—C6	1.484 (4)	F6a—C6	1.334 (3)
F3a—C3	1.330 (3)	F3c—C3	1.319 (3)
C16—C15	1.374 (4)	C18—C17	1.388 (3)
C16—C17	1.373 (4)	C2—C3	1.491 (4)
**Atoms**	**Anglesº**	**Atoms**	**Anglesº**
C1—Si1—C4	104.52 (10)	C14—C13—C18	117.7 (2)
C13—Si1—C4	105.48 (10)	C17—C18—C13	120.3 (3)
C13—Si1—C1	108.99 (11)	C1—C2—F2	121.5 (2)
C7—Si1—C4	109.15 (10)	C3—C2—F2	110.2 (2)
C7—Si1—C1	113.14 (10)	C3—C2—C1	128.4 (2)
C7—Si1—C13	114.77 (10)	C9—C10—C11	120.1 (2)
F4—C4—Si1	113.03 (15)	C18—C17—C16	120.6 (2)
C5—C4—Si1	132.54 (19)	C12—C7—Si1	118.60 (17)
C5—C4—F4	114.4 (2)	C8—C7—Si1	123.48 (18)
F5—C5—C4	121.6 (2)	C8—C7—C12	117.9 (2)
C6—C5—C4	127.9 (2)	F3a—C3—F3b	107.4 (2)
C6—C5—F5	110.5 (2)	F3c—C3—F3b	108.0 (2)
C17—C16—C15	120.0 (2)	F3c—C3—F3a	107.2 (2)
C10—C11—C12	119.9 (2)	C2—C3—F3b	111.3 (2)
C7—C12—C11	121.1 (2)	C2—C3—F3a	110.9 (2)
C14—C15—C16	119.8 (3)	C2—C3—F3c	111.8 (2)
C7—C8—C9	121.1 (2)	C13—C14—C15	121.6 (2)
C10—C9—C8	119.9 (2)	C5—C6—F6c	112.3 (2)
F1—C1—Si1	114.56 (16)	F6b—C6—F6c	108.0 (2)
C2—C1—Si1	131.75 (18)	F6b—C6—C5	111.7 (2)
C2—C1—F1	113.7 (2)	F6a—C6—F6c	106.7 (2)
C18—C13—Si1	122.35 (19)	F6a—C6—C5	111.4 (2)
C14—C13—Si1	119.92 (17)	F6a—C6—F6b	106.5 (2)

5.References

[1] P.W. Betteridge, J.R. Carruthers, R.I. Cooper, K. Prout, D.J. Watkin, *J. Appl. Cryst.*, **36**, 2003, 1487–1493.

[2] O.V. Dolomanov, L.J. Bourhis, R.J. Gildea, J.A. Howard, H. Puschmann, *J. Appl. Cryst.,* **42**, 2009, 339–341.

[3] G.M. Sheldrick, *Acta Cryst.*, **64**, 2008,112–122.

[4] T.R. Schneider, G.M. Sheldrick, *Acta Cryst.*, **58**, 2002, 1772–1779.

[5] L.J. Farrugia, WinGX, *J. Appl. Cryst.*, **45**, 2012, 849–854.

[6] C.F. Macrea, P.R. Eddington, P. McCabe, E. Pidcock, G.P. Shields, R. Taylor, M. Towler, J. van de Streek, *J. Appl. Cryst.*, **39**, 2006, 453–457.

[7] A.L. Spek, *Acta Cryst. D*, **65**, 2009, 148–155.
